# Bacteria sense the antibiotic rifampicin through a widespread dual-promoter based alarm system

**DOI:** 10.1093/nar/gkaf1407

**Published:** 2026-01-14

**Authors:** Petra Sudzinová, Tamara Knežová Balgová, Marek Schwarz, Klára Juříková Mikesková, Karolína Hegrová, Dragana Vítovská, Priyanka Rawat, Šárka Bobková, Veronika Kočárková, Saran Natarajan, Debora Pospíšilová, Alena Křenková, Martin Hubálek, Petr Halada, Ivan Barvík, Tomáš Koval', Jan Dohnálek, Jana Wiedermannová, Hana Šanderová, Libor Krásný

**Affiliations:** Laboratory of Microbial Genetics and Gene Expression, Institute of Microbiology of the Czech Academy of Sciences, Vídeňská 1083, 142 20 Prague, Czech Republic; Laboratory of Microbial Genetics and Gene Expression, Institute of Microbiology of the Czech Academy of Sciences, Vídeňská 1083, 142 20 Prague, Czech Republic; Department of Genetics and Microbiology, Faculty of Science, Charles University, Viničná 5, 128 44 Prague, Czech Republic; Laboratory of Microbial Genetics and Gene Expression, Institute of Microbiology of the Czech Academy of Sciences, Vídeňská 1083, 142 20 Prague, Czech Republic; Laboratory of Bioinformatics, Institute of Microbiology of the Czech Academy of Sciences, Vídeňská 1083, 142 20 Prague, Czech Republic; Laboratory of Microbial Genetics and Gene Expression, Institute of Microbiology of the Czech Academy of Sciences, Vídeňská 1083, 142 20 Prague, Czech Republic; Laboratory of Microbial Genetics and Gene Expression, Institute of Microbiology of the Czech Academy of Sciences, Vídeňská 1083, 142 20 Prague, Czech Republic; Laboratory of Microbial Genetics and Gene Expression, Institute of Microbiology of the Czech Academy of Sciences, Vídeňská 1083, 142 20 Prague, Czech Republic; Laboratory of Microbial Genetics and Gene Expression, Institute of Microbiology of the Czech Academy of Sciences, Vídeňská 1083, 142 20 Prague, Czech Republic; Laboratory of Microbial Genetics and Gene Expression, Institute of Microbiology of the Czech Academy of Sciences, Vídeňská 1083, 142 20 Prague, Czech Republic; Laboratory of Microbial Genetics and Gene Expression, Institute of Microbiology of the Czech Academy of Sciences, Vídeňská 1083, 142 20 Prague, Czech Republic; Department of Genetics and Microbiology, Faculty of Science, Charles University, Viničná 5, 128 44 Prague, Czech Republic; Laboratory of Microbial Genetics and Gene Expression, Institute of Microbiology of the Czech Academy of Sciences, Vídeňská 1083, 142 20 Prague, Czech Republic; Laboratory of Microbial Genetics and Gene Expression, Institute of Microbiology of the Czech Academy of Sciences, Vídeňská 1083, 142 20 Prague, Czech Republic; Department of Mass Spectrometry of Biopolymers, Institute of Organic Chemistry and Biochemistry of the Czech Academy of Sciences, Flemingovo náměstí 542/2, 160 00 Prague, Czech Republic; Department of Mass Spectrometry of Biopolymers, Institute of Organic Chemistry and Biochemistry of the Czech Academy of Sciences, Flemingovo náměstí 542/2, 160 00 Prague, Czech Republic; Laboratory of Structural Biology and Cell Signaling, Institute of Microbiology of the Czech Academy of Sciences, Vídeňská 1083, 142 20 Prague, Czech Republic; Institute of Physics, Faculty of Mathematics and Physics, Charles University, Ke Karlovu 5, 121 16 Prague 2, Czech Republic; Laboratory of Structure and Function of Biomolecules, Institute of Biotechnology of the Czech Academy of Sciences, Průmyslová 595, 252 50 Vestec, Czech Republic; Laboratory of Structure and Function of Biomolecules, Institute of Biotechnology of the Czech Academy of Sciences, Průmyslová 595, 252 50 Vestec, Czech Republic; Laboratory of Microbial Genetics and Gene Expression, Institute of Microbiology of the Czech Academy of Sciences, Vídeňská 1083, 142 20 Prague, Czech Republic; Laboratory of Microbial Genetics and Gene Expression, Institute of Microbiology of the Czech Academy of Sciences, Vídeňská 1083, 142 20 Prague, Czech Republic; Laboratory of Microbial Genetics and Gene Expression, Institute of Microbiology of the Czech Academy of Sciences, Vídeňská 1083, 142 20 Prague, Czech Republic

## Abstract

Most antibiotics are natural compounds or their derivatives, and bacteria have evolved defensive mechanisms to resist them. Many of these mechanisms are still poorly understood or unknown. This study reveals that in *Bacillus subtilis*, the transcription factor HelD increases resistance to rifampicin by protecting its target, RNA polymerase (RNAP). This protection is mediated by the HelD N-terminal domain that penetrates into RNAP to the close vicinity of the rifampicin binding pocket. Importantly, the bacterium detects low rifampicin levels using a unique regulatory system involving two convergent promoters with finely tuned kinetic properties. In the absence of rifampicin, the stronger antisense promoter inhibits transcription from the sense promoter. In the presence of subinhibitory rifampicin concentration, the antisense promoter is more likely to encounter rifampicin-bound RNAP. This relieves the repression from the sense promoter, increasing its transcription by almost two orders of magnitude, boosting *helD* expression. A similar two-promoter arrangement also controls the *pps* gene, which encodes a rifampicin-modifying enzyme. These findings define a widespread bacterial response system sensitive to rifampicin, as this dual-promoter architecture is conserved across many bacterial species and found upstream of genes potentially involved in rifampicin resistance, such as those for hydrolases, transporters, and transferases.

## Graphical Abstract

**Graphical Abstract:**
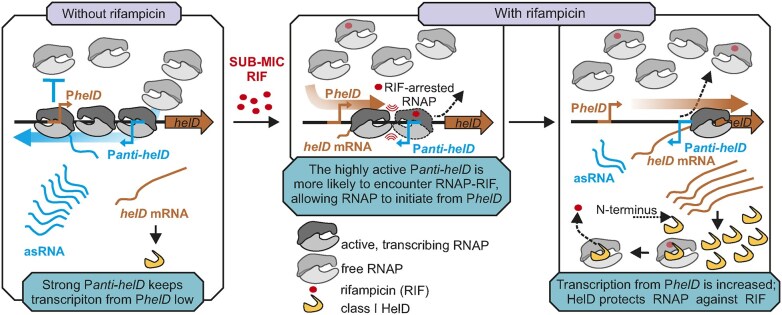

## Introduction

Increasing resistance of bacteria to antibiotics requires new strategies to fight bacterial infections. An important prerequisite for the development of these strategies is the identification and understanding of the mechanisms that bacteria naturally possess to defend themselves against antibiotics. These mechanisms do not rely on mutations but have evolved over long periods of time due to cohabitation of the same niches by antibiotic producers and sensitive species. These mechanisms allow the bacterium to sense low levels of the antibiotic and mount a defensive action (*e.g*. Tamae *et al.* [1]). Overexpression of respective genes may lead to high resistance.

Despite decades of research, the identification and understanding of these mechanisms is still understudied, as are mechanisms that control the expression of respective proteins. This is exemplified by HelD.

HelD is a transcription factor that binds to bacterial RNA polymerase (RNAP), simultaneously penetrating both the primary (DNA binding) and secondary (entry of NTPs to the active site) channels [2–5]. HelD resolves stalled complexes of RNAP on DNA, allowing subsequent rounds of transcription and preventing transcription-replication collisions, thereby helping maintain the chromosome integrity [6]. Recently, HelD was shown to dissociate RNAP complexes arrested on promoter DNA during the early stages of transcription by the action of the antibiotic rifampicin (RIF); even more importantly, HelD promotes the release of RIF from RNAP [7, 8]. RIF and its variants bind to RNAP at the bottom of the DNA/RNA exit channel and sterically prevent synthesis of RNA beyond 2–3 nucleotides (nt) [9, 10]. The protective property of HelD against RIF was described in Actinobacteria, and it was proposed that it is specific for this phylum due to a specific structural feature [8].

Structurally, at least two classes of HelD proteins exist: Class I (Firmicutes such as *Bacilli, Staphylococci*) that contain a relatively long N-terminal domain that penetrates deep into the secondary channel, reaching beyond the active site [3], and Class II (Actinobacteria such as *Mycobacteria, Streptomyces*) where the N-terminal domain is shorter than its counterpart in Class I HelD [2] (for a comparison of 3D structures of Class I and II HelD proteins see Supplementary Fig. S1). Importantly, Class II HelDs contain the primary channel loop (PCh loop, absent from Class I) that reaches close to the active site and is believed to distort the RIF binding pocket. Dissociation of RIF from RNAP was demonstrated with Class II HelDs (alternative name HelR), and it was proposed that Class I HelDs do not possess this ability [7].

To provide an unbiased view of the defense mechanisms against RIF encoded in the genome of a model Firmicute bacterium, *Bacillus subtilis*, and perhaps elucidate the potential involvement of Class I HelD in this process, including its expression control, we performed a screen for proteins upregulated in the presence of a sub-inhibitory concentration of RIF. HelD was identified by this screen as one of the most upregulated proteins. Subsequent experiments revealed a direct protective effect of *B. subtilis* HelD on transcription in the presence of RIF mediated by its N-terminal domain.

The most striking and counterintuitive discovery, however, was that expression of the *helD* gene was induced by RIF at the transcriptional level, regulated by an interplay between two convergent promoters, a regulatory setup that we identified also upstream of another RIF-induced gene, *pps*, encoding phosphotransferase. Similar two-promoter regulatory regions were then identified upstream of other genes (including *helD* and *pps*) in different species, demonstrating the evolutionary utility of this mechanism.

## Materials and methods

### DNA manipulations

Plasmids (and respective strains) are listed in Supplementary Table S1 and primers in Supplementary Table S2.

#### Cloning of variants of the upstream regions of helD/pps genes

Variants of *helD/pps* upstream regions were amplified from *Bsu* wt genomic DNA (BsB1, LK2711) with primers listed in Supplementary Table S2 by PCR [Expand High Fidelity PCR System (Roche, Cat No. 11 732 650 001)]. CORE constructs of P*helD*, P*anti-helD*, P*pps*, P*anti-pps*, P*helD* CORE+15, and chimeric constructs were created by annealing complementary oligonucleotides (with included respective overhangs to mimic open *EcoRI, HindIII* restriction sites). The annealing was done in water in a PCR cycler (BioRad, T100^TM^ Thermal Cycler) with a temperature gradient starting at 95°C and decreasing by 1°C every 1 min (70 cycles).

Mutagenesis of promoter constructs was done with primers carrying respective mutations and the Restriction Free Cloning system [11], according to the manufacturer’s instructions using Phusion polymerase (NEB).

pRLG770, a general promoter vector [12] was used to create promoter constructs used in transcriptions *in vitro*. Promoter fragments were inserted via *EcoRI* and *HindIII* restriction sites. For promoter activity experiments in *B. subtilis*, promoter–*lacZ* fusions were constructed using the integrative vector pDG3661 [13]. Promoter fragments were inserted via *EcoRI* and *HindIII* restriction sites. All constructs were verified by DNA sequencing.

#### Cloning of constructs for HelD overexpression and complementation strains

The *helD* coding region plus its 5′ and 3′ flanking regions containing the promoter and terminator were amplified [Expand High Fidelity PCR System (Roche, Cat No. 11 732 650 001)] from wt *Bsu* genomic DNA (LK2711, BsB1) with primers #3673 + #3628. Using the Restriction Free Cloning system [11], the fragment was cloned into pDG3661 to yield LK2931.

#### Cloning of mutagenized versions of HelD for in vivo experiments

DNA fragments encoding shortened HelD (HelDΔN) and HelD with mutations in the tip of the N-terminal domain (HelDmutTIP: D56A, D57A, E60A) were created using the Restriction Free Cloning system, using primers #4746 or #4747, and the plasmid carrying the native copy of *helD* (LK2931) was used as the template. The resulting pDG3661-based plasmids were named LK3745 (HelDΔN) and LK3771 (HelDmutTIP).

### Competent cell preparation and transformation

Competent cell preparation and transformations were done by standard protocols according to Hanahan [14] for *E. coli* and to Dubnau [15] for *B. subtilis*, and selection for appropriate antibiotics. Double cross-over recombinants at *amyE* of *B. subtilis* (pDG3661-based constructs; [13]) were selected for resistance to chloramphenicol (5 μg/ml) and sensitivity to spectinomycin (100 μg/ml) as described in [16].

### Strains and strains construction

All strains used in this study are listed in Supplementary Table S1.

#### Δ*helD and mutated helD strain preparation*

gDNA of Δ*helD* from the BKE library (BKE33450; [17]) was transformed into competent wt BsB1 cells (LK2711; selection for MLS), resulting in Δ*helD* (LK2840). Plasmid LK2931 was used to transform wt BsB1 cells (LK2711) to yield *helD* over-expression strain (*helD* OE; LK2934) or Δ*helD* (LK2840) to get *helD* complementation (*helD* COM; LK2935) strain, respectively. Plasmids LK3745 and LK3771 were used to transform Δ*helD* (LK2840), resulting in Δ*helD* complemented with HelDΔN (LK3772) or HelDmutTIP (LK3784).

#### RIF-resistant B. subtilis with a mutation in RNAP

Wt BsB1 cells (LK2711) were streaked on the LB agar plate and incubated at 37°C overnight. The next day, a single colony was inoculated into 10 ml of LB medium and incubated overnight at 37°C. Then, the strain was inoculated to an OD_600_ of 0.1 into 10 ml LB and let grow till it reached OD_600_ ∼ 1.0 and then an aliquot (200 μl) was plated onto an LB agar plate supplemented with 1 μg/ml rifampicin. After overnight growth, several colonies were selected for gDNA isolation, and the mutations were identified by sequencing. A strain (LK4387) bearing the C1444T mutation in *rpoB*, causing the H482Y mutation in β, was selected for further studies. This mutation is known to confer resistance to rifampicin [18].

#### Strains bearing promoter-lacZ fusions

Variants of *helD/pps* promoter regions in cloned pDG3661 plasmid were transformed into wt BsB1 (LK2711), Δ*helD* (LK2840), or RIF^R^ (LK4387). Also, an empty pDG3661 plasmid was transformed to create “promoterless”, background control (LK1933).

#### Strain-bearing RNAP-FLAG fusion

*B. subtilis* strain containing FLAG peptide on the C-terminus of the β' subunit was prepared by homology recombination. The construct for homology recombination contained (i) left arm - 523 bp of the 3′ part of the *rpoC* gene without stop codon (primers #3583+#3584), (ii) sequence encoding the FLAG-peptide (5′ GACTACAAAGACGATGACGACAAG 3′) + STOP codon (TAA) + natural terminator (primers #3624+#3586), (iii) sequence encoding a spectinomycin resistance cassette (primers #3587+#3588) and (iv) right arm - 544 bp downstream region of the *rpoC* gene terminator (primers #3589+#3590) and was inserted using Gibson assembly method into the pUC18 vector. The primers contained homologous overlaps, and the individual parts were synthesized by PCR using *B. subtilis* chromosomal DNA as the template (strain LK2711, BsB1) for (i), (ii), and (iv) parts or plasmid pDR110 ([19]; for the *spc* resistance cassette), respectively. pUC18 was digested with *BamHI* and *EcoRI* restriction enzymes. The construct was assembled by Gibson Assembly® Cloning Kit (New England Biolabs, E5510S) following the manufacturer’s protocol and subsequently transformed into *E. coli* DH5alpha competent cells. The resulting construct (LK2982) was verified by sequencing and then transformed into *B. subtilis* BsB1 strain (LK2711). The resulting *B. subtilis* RNAP-FLAG strain (LK3132) was analyzed by immunoprecipitation using ANTI-FLAG® M2 Affinity Gel (Sigma Aldrich, A2220).

#### Δ*pps and* Δ*helD*Δ*pps strain construction*

Δ*pps* gDNA from BKK library (BKK18830; [17]) was transformed into wt BsB1 (LK2711; selection for Kan), resulting in Δ*pps*; LK3449 or into Δ*helD* (LK2840), creating a double deletion strain Δ*helD*Δ*pps* (LK3451).

### Media, growth conditions, and antibiotics

Cells were grown in liquid LB at 37°C with vigorous shaking. For growth on solid media, 1.5% agar LB plates were used. Antibiotics were used at the following concentrations: ampicillin 100 μg/ml, chloramphenicol 5 μg/ml, MLS (erythromycin 0.5 μg/ml plus lincomycin 12.5 μg/ml), kanamycin 5 μg/ml, spectinomycin 100 μg/ml. Different rifampicin concentrations [working solutions (diluted in ethanol) were freshly prepared prior to every experiment or working solutions (diluted in DMSO) were kept and reused] were used as indicated, depending on the experiment and manufacturer.

**Figure 1.**
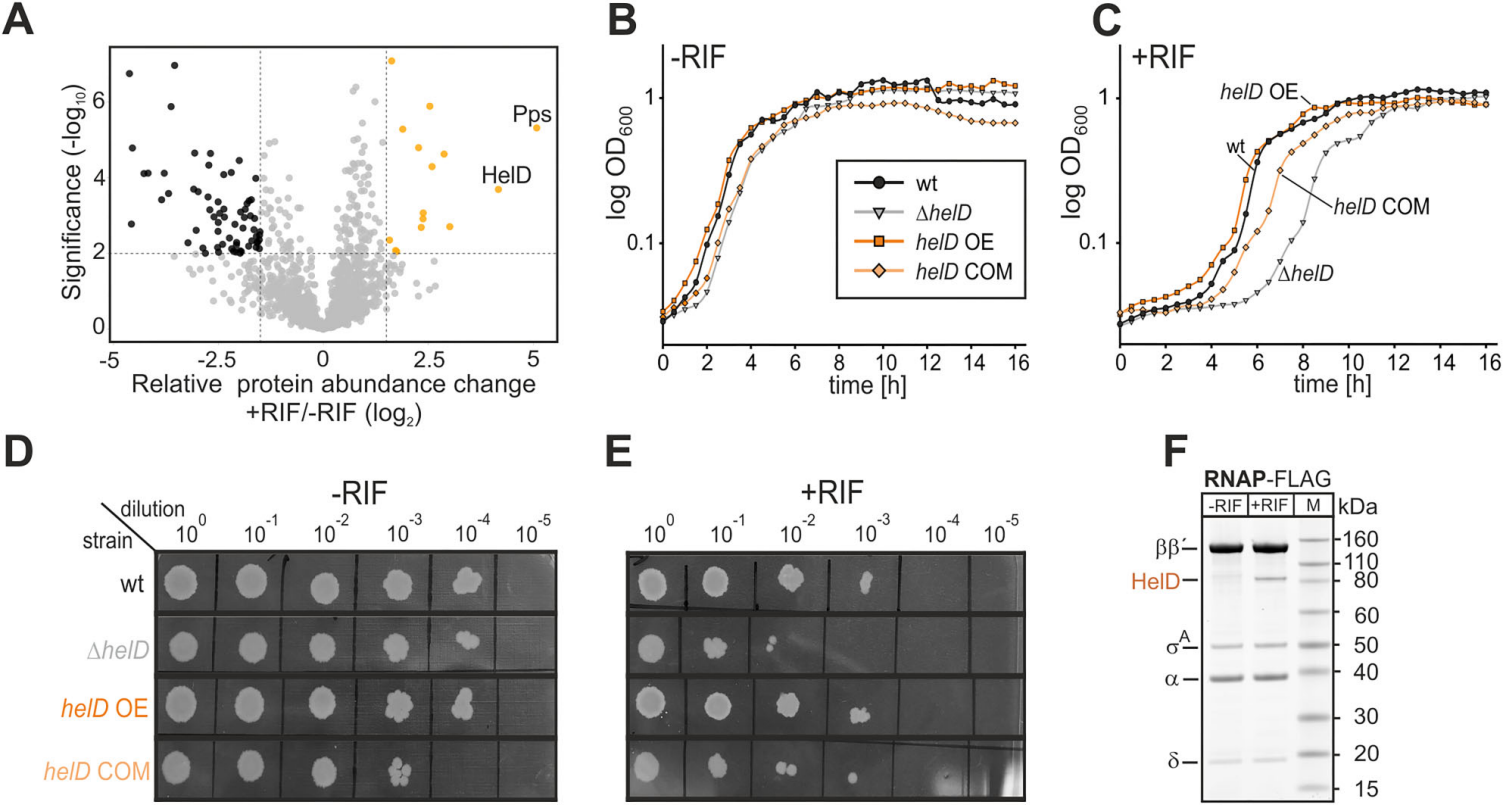
**Effect of HelD on RIF resistance of *B. subtilis*. (A)** Quantitative mass spectrometry analysis of *B. subtilis* (*Bsu*) wt strain (LK2711) in the presence vs absence of sub-MIC concentration (0.03 μg/ml) of RIF. The analysis was done from six biological replicates. The abundance of individual proteins was compared by a two-tailed Student’s *t*-test. The permutation-based FDR was used as an adjustment of the *p*-value. The enrichment is shown with a volcano plot (−log_10_  *p*-value > 2 on the *y*-axis, protein enrichments > 1.5 on the *x*-axis). Significantly enriched (upregulated) proteins are shown on the right hand side as light orange dots, significantly downregulated proteins on the left hand side as black dots. The identity of the two most enriched proteins is indicated (HelD, Pps); for the full list, see Supplementary Table S5. **(B), (C)** Growth of strains in liquid LB medium in the absence (-RIF) or presence (+RIF) of sub-MIC concentration (0.03 μg/ml) of RIF. The strains were inoculated in a 24-well plate, and the OD_600_ was measured every 30 min for ∼16 h. A representative result is shown from a total of three independent growth experiments. Black dots, wt strain (LK2711); grey triangles, Δ*helD* strain (LK2840); orange squares, *helD* overexpression strain (LK2934); light orange diamonds, *helD* complementation strain (LK2935). **(D), (E)** Growth of strains on solid LB medium in the absence (-RIF) or presence (+RIF) of sub-MIC concentration (0.03 μg/ml) of RIF. A representative result is shown; the experiment was repeated three times with identical results. **(F)** A representative SDS-PAGE of pull-down of FLAG-tagged RNAP (LK3132) in the absence (-RIF) or presence (+RIF) of sub-MIC concentration (0.03 μg/ml) of RIF. The identity of HelD was determined by mass spectrometry. The experiment was performed in three biological replicates.

The initial experiments (proteome analysis, Fig. 1A), phenotypic experiments (Fig. 1D and E), pull down (Fig. 1F), treatment of cells for Northern blot analysis and RT-qPCRs (Figs. 3A and 4E and F), RACE (Fig. 3B) were performed with EREMFAT i.v. 600 mg rifampicin [Riemsere, Arzneimittel AG] at 0.03 µg/ml, diluted in ethanol and freshly prepared prior to every experiment. During the project, however, we ran out of our EREMFAT rifampicin stock. As the same product was not commercially available anymore, we purchased rifampicin from Sigma (Sigma R3501). With EREMFAT rifampicin, the highest induction of the P*helD* FULL construct (LK3005) in β-galactosidase assays was at 0.036 µg/ml rifampicin (this value was due to serial dilution of the antibiotic). With Sigma R3501 rifampicin, however, the highest induction of the P*helD* FULL construct (LK3005) was observed at 0.004 µg/ml rifampicin. A side-by-side comparison of the two rifampicins in β-galactosidase assays with the LK3005 construct is shown in (Supplementary Fig. S2). At this point, we repeated β-galactosidase assays with P*helD* FULL (LK3005), CORE (LK2970), UP (LK3038), and DOWN (LK3004) constructs with 0.004 µg/ml Sigma R3501 rifampicin (we already had these constructs tested with EREMFAT rifampicin) and obtained the same results as previously with 0.036 µg/ml EREMFAT rifampicin (Supplementary Fig. S2). With Sigma R3501 rifampicin, the following experiments were performed: all β-galactosidase assays in the main text and supplements (diluted in ethanol), all transcriptions *in vitro* and determination of minimum inhibitory concentrations (diluted in DMSO).

### Whole proteome analysis – preparation of cell lysates

Wt *B. subtilis* cells (LK2711, BsB1) were grown into the exponential phase (OD_600_ ∼ 0.5) in 100 ml LB. Then the culture was divided into two Erlenmeyer flasks, and one was treated with sub-MIC RIF (0.03 μg/ml) and the other was untreated. After 1 h, 25 ml of cells were collected by centrifugation (10 min; 5 251 g; 4°C) from each flask, washed with 1 ml of Lysis Buffer (20 mM Tris-HCl, pH 8.0, 150 mM KCl, 1 mM MgCl_2_; this is the only type of Lysis Buffer used in this study) and stored frozen at −80°C. Pellets were then resuspended in 150 μl Lysis Buffer with 0.5 mM DTT, 0.5 mM PMSF, and protease inhibitor cocktail (5 μl/ml, Sigma, P8849). Cells were then disrupted using sonication (Hielscher UP200S) for 5 × 20 s (1 mm tip diameter probe, amplitude 50%) on ice with 1 min pauses in between. Samples were centrifuged (10 min; 20 627 g; 4°C) to remove debris, and the concentration of proteins from the supernatant was determined using the Bradford assay [20]. The experiment was repeated six times, each replicate on a different day.

### Mass spectrometry analysis

#### Sample preparation

Proteins were digested with 0.1 μg of trypsin dissolved in 50 mM ammonium bicarbonate at 37 °C for 16 h. The resulting peptides were separated on an UltiMate 3000 RSLCnano system (Thermo Fisher Scientific) coupled to an Orbitrap Fusion Lumos mass spectrometer (Thermo Fisher Scientific). The peptides were trapped and desalted with 2% acetonitrile in 0.1% formic acid at a flow rate of 30 μl/min on an Acclaim PepMap100 column [5 μm, 5 mm by 300-μm internal diameter (ID); Thermo Fisher Scientific]. The eluted peptides were separated using an Acclaim PepMap100 analytical column (2 μm, 50 cm by 75 μm ID, Thermo Fisher Scientific). The 125-min elution gradient at a constant flow rate of 300 nl/min was set to 5% of phase B (0.1% of formic acid in 99.9% of acetonitrile) and 95% of phase A (0.1% of formic acid) for 1 min, after which the content of acetonitrile was gradually increased. The Orbitrap mass range was set from m/z 350 to 2000 in the MS mode, and the instrument in Data dependent acquisition (DDA) mode acquired HCD fragmentation spectra for ions of m/z 100–2000.

#### Protein identification and quantification

MaxQuant with Andromeda search engine (version 1.6.3.4; Max–Planck-Institute of Biochemistry, Planegg, Germany) was utilized for peptide and protein identification and identification with databases of the *Bacillus subtilis* proteome and common contaminants. The following settings were used: Fixed modification: carbamidomethyl (C); variable modifications: oxidation (M), acetyl (protein N-term); enzyme: Trypsin/P, 2 missed cleavages allowed; match between runs was enabled. Mass tolerance for the peptide first and main search was set as 20 and 4.5 ppm, respectively. Minimal peptide length was set as 7. FDR level was set as 0.01 for both peptides and proteins. The dataset of samples treated with rifampicin was searched together with the dataset without rifampicin. Perseus software (version 1.6.2.3; Max–Planck-Institute of Biochemistry) was used for the label-free quantification of proteins from a wild-type *Bacillus subtilis* strain in the presence of rifampicin compared to a negative control, the same strain in the absence of rifampicin. The identified proteins were filtered for contaminants and reverse hits. Proteins detected in the data were filtered to be quantified in at least three of the six replicates. The data were processed to compare the abundance of individual proteins by statistical tests in the form of Student’s *t*-test and resulting in a volcano plot comparing the statistical significance (two-sided *p*-value) and protein-abundance difference (fold change).

The mass spectrometry proteomics data have been deposited to the ProteomeXchange Consortium via the PRIDE [21] partner repository with the dataset identifier PXD066046 and 10.6019/PXD066046.

### Phenotypic experiments – liquid media

On day 1, tested strains were streaked on LB agar plates supplemented with the respective antibiotics (used to create the deletions) or without (wt) and allowed to grow overnight. On day 2, a colony from each plate was inoculated into 10 ml of LB (no antibiotics) and let grow for ∼ 6 h. Then, each strain was inoculated into 1 ml of LB to an OD_600_ of 0.03 without or with sub-MIC of rifampicin (0.03 μg/ml) in a 24-well Costar 24 Flat Transparent Plate (NUNCLON^TM^ Δ Surface, NUNC^TM^, Thermo Fisher Scientific). The growth was monitored in a TECAN THE SPARK® MULTIMODE MICROPLATE READER, using a humidity cassette. The OD_600_ was recorded every 30 min for 16 h. The experiments were performed at least three times on different days.

### Phenotypic experiments – solid media

On day 1, tested strains were inoculated into 10 ml of LB with the respective antibiotics (used to create the deletions) or without (wt) and let grow overnight. On day 2, the strains were inoculated into 10 ml of LB (no antibiotics) and let grow into the early exponential phase (OD_600_ ∼ 0.3). The cells were then serially diluted into LB without antibiotics (down to 10^−5^), and 2 μl per dilution was spotted onto LB agar plates without or with sub-MIC of RIF (0.03 μg/ml). Plates were then incubated at 37°C for 48 h. The experiments were performed at least three times on different days.

### Minimum Inhibitory Concentration

Minimum Inhibitory Concentration (MIC) of rifampicin against wt BsB1 (LK2711), Δ*helD* (LK2840), *helD* COM (LK2935), *helD* OE (LK2934), HelDΔN (LK3772), HelDmutTIP (LK3784), Δ*pps* (LK3449), and Δ*helD*Δ*pps* (LK3451) were determined by the broth microdilution method. All eight strains were streaked into appropriate LB agar plates with or without antibiotics. Single colonies were inoculated into 10 ml LB containing appropriate antibiotics and grown overnight at 37°C with constant shaking at 180 rpm (Ø25 mm, Multitron Standard, Infors HT, Biotrade). The next day, the cultures were inoculated into 10 ml LB without antibiotics to a final OD_600_ of 0.03. All cultures were then grown till OD_600_ reached 1.0. The cultures were further diluted 1000 times in fresh LB, and 50 µl was added to each well containing 50 µl of varying concentrations of rifampicin-containing LB in a 96-well microtiter plate (Thermo Fisher Scientific, Nunc™ Edge™ 96-Well, 167 425). Rifampicin gradients were prepared by two-fold serial dilution with 0.25 µg/ml as the highest concentration and 0.004 µg/ml as the lowest. Rifampicin solution was prepared by dissolving in 100% DMSO. The plates were incubated at 37°C for 24 h with constant shaking at 120 rpm (Ø20 mm, IKA^®^ HS 260 Basic). Cell viability was determined by the Resazurin Microtiter Plate Assay. 1 mg/ml resazurin (Sigma, R7017-1G) solution was prepared in sterile water, and 10 µl was added to each well after 24 h of incubation. The plates were further incubated for 3–4 h, and MIC was determined as the lowest concentration in which the well remained blue (pink is viable and blue is not viable). After the resazurin assay, the plates were scanned with an Epson Perfection V850 Pro scanner.

### Pull down

The day before, the strains were inoculated into 10 ml of LB with respective antibiotics and allowed to grow overnight. The next day, the strains were inoculated into fresh LB media (without antibiotics) and allowed to grow into the exponential phase (OD_600_ ∼ 0.5) in 500 ml LB. The culture was then equally divided into two parts; one part was treated with sub-MIC of rifampicin (0.03 μg/ml) and the other was left untreated. The cells were then incubated for another 1 h at 37°C with vigorous shaking. 100 ml of cells were then pelleted by centrifugation (10 min; 10 000 g; 4°C), the pellets were resuspended in Lysis Buffer and pelleted again. The pellets were stored at –20°C. Pellets were resuspended in 3 ml of Lysis Buffer supplemented with 1 mM DTT, 0.5 mM PMSF and protease inhibitor cocktail (5 μl/ml, Sigma, P8849), sonicated 15 × 10 s (Hielscher UP200S, 1 mm tip diameter probe, 50% amplitude) with 1 min pauses on ice and centrifuged (10 min; 20 627 g; 4°C). Concentration of proteins from the supernatant was determined using the Bradford assay [20]. Around 5 mg of total protein (in 1.6 ml volume of Lysis Buffer) was incubated on a rotator (Multi Bio RS-24, Biosan, 20 orbital rpm) with 40 μl of ANTI-FLAG® M2 Affinity Agarose gel (Sigma, A2220) overnight at 4°C. Agarose beads with the captured protein complexes were then 4x washed with 0.5 ml of Lysis Buffer (composition as used above; after each washing, the beads were spun down, the supernatant discarded). FLAG-tagged proteins in complexes were eluted from beads by 40 μl of 3x FLAG ® Peptide (Sigma, F4799) [diluted in Tris-buffered saline (TBS, pH 7.6) to a final concentration of 150 µg/ml]. Proteins were analyzed on SDS-PAGE and selected bands were identified by mass spectrometry.

### Trypsin digestion and MALDI FT-ICR mass spectrometry

CBB-stained protein bands were cut out from gels, chopped into small pieces, and destained using 50 mM 4-ethylmorpholine acetate (pH 8.1) in 50% acetonitrile (MeCN). After washing with water and MeCN, the proteins were digested overnight at 37°C using sequencing-grade trypsin (100 ng; Promega) in a buffer containing 25 mM 4-ethylmorpholine acetate and 5% MeCN. The resulting peptides were extracted with 40% MeCN/0.2% TFA (trifluoroacetic acid). For MALDI MS analysis, 0.5 µl of each peptide mixture was deposited on the MALDI plate, air-dried at room temperature, and overlaid with 0.5 µl of the matrix solution (α-cyano-4-hydroxycinnamic acid in 50% acetonitrile/0.1% TFA; 5 mg/ml, Sigma). Peptide mass maps were measured using a 15T Solarix XR FT-ICR mass spectrometer (Bruker Daltonics) and calibrated internally using peptide masses of *B. subtilis* RpoB and RpoC proteins. The peak lists generated using the DataAnalysis 5.0 program were searched against the UniProtKB database using the in-house MASCOT engine v.2.7 with the following settings: taxonomy *B. subtilis*, peptide tolerance of 3 ppm, missed cleavage site set to two, and variable oxidation of methionine.

### RT-qPCR

Cells were streaked out on LB agar plates, grown overnight, diluted to an OD_600_ of 0.03 in fresh LB, and grown to an OD_600_ ∼ 0.5 in 100 ml of LB; the culture was then divided into two equal parts, and one part was treated with sub-MIC rifampicin (see Media and growth conditions for specification), and the other was left untreated. After 1 h of further cultivation, 2 ml of cells were withdrawn and treated with RNAprotect® Bacteria reagent (QIAGEN, 76 506) according to the manufacturer’s instructions, pelleted, immediately frozen, and stored at −80°C. RNA was isolated with RNeasy® Mini Kit (QIAGEN, 74 104), and recovery marker RNA (RM RNA) was added (5 μl, 1 ng/μl per reaction) at the time of extraction to control for differences in degradation and pipetting errors during extraction. RM RNA was a fragment of 16S rRNA from *M. smegmatis* prepared by *in vitro* T7 RNAP (NEB)-dependent transcription from a DNA fragment prepared by PCR with primers #1281 and #1282 and *M. smegmatis mc^2^155* chromosomal DNA purified with Mini Bacterial Kit (Invitrogen) from LK2980. Finally, RNA was DNase-treated (TURBO DNA-*free*^TM^ Kit, Invitrogen, AM1907) according to the manufacturer’s instructions. Total RNA was reverse transcribed to cDNA with reverse transcriptase (SuperScript™ III Reverse Transcriptase, Invitrogen, 18080–044), using random hexamer primers, and this was followed by qPCR in a LightCycler 480 System (Roche Applied Science) containing LightCycler® 480 SYBR Green I Master (Roche, Cat No. 04 887 352 001) and 0.5 μM primers (each). The ΔCt method was used to determine the relative quantities of cDNAs [22]. The final data were normalized to RM amounts and cell density (OD_600_).

### Rapid Amplification of cDNA Ends (5′RACE)

The transcription start site (TSS) of the *helD* gene was determined by 5′RACE, following the protocol from [23] with a few modifications. Briefly, wt BsB1 cells (LK2711) were grown in LB. Three ml of cells from the exponential (OD_600_ ∼ 0.5) and 2 ml from the stationary (2 h after cessation of exponential growth) phases were withdrawn and treated with RNAprotect® Bacteria reagent (QIAGEN) according to the manufacturer’s instructions, pelleted, and immediately frozen (−80°C). RNA was isolated with RNeasy® Mini Kit (QIAGEN) and DNase-treated (TURBO DNA-free Kit, Ambion). Two μg of RNA was treated with RPPH (NEB) to remove the native 5′ triphosphates of primary transcripts or left untreated. Samples were purified using RNA Clean & Concentrator™-25 kit (Zymo) according to the manufacturer’s instructions. Then, an adapter oligo SSS1016 [23] was ligated to the RNA 5′end using T4 RNA ligase (NEB) at 16°C overnight. Samples were again purified with RNA Clean & Concentrator™-25 kit, and reverse transcribed to cDNA with reverse transcriptase (SuperScript™ III Reverse Transcriptase, Invitrogen) using a *helD* gene-specific primer #3699. Reactions in the absence of reverse transcriptase were performed to control for genomic DNA contamination. This was followed by the PCR amplification, using SSS1017 and #3698 primers. For the PCR reaction, a touchdown protocol was used as in [23]. Products of PCR were run on agarose gel, cut out, purified with QIAquick® Gel Extraction Kit (QIAGEN, 28 706), and then sequenced using the #3698 primer.

The same 5′ end of *helD* RNA was obtained from the exponential and transition phase, and also from the RNA isolated from the cells treated with the sub-MIC of RIF.

### β-galactosidase assay

The strains were streaked out onto LB plates supplemented with Cm. The next day, the cells were transferred to 5 ml of liquid LB and grown overnight. The next day, the cells were diluted into 100 ml fresh LB to an OD_600_ ∼ 0.03. The strains were then grown in LB to the exponential phase (OD_600_ ∼ 0.5; time zero). 1 ml of cells was collected at this time point. The culture was then divided into two equal parts (10 ml each), and one part was treated with sub-MIC of rifampicin (see Media and growth conditions for specification) while the other part was left untreated. The cells were allowed to grow for another 1 h, 1 ml of cells was collected from each part, centrifuged (10 min; 12 000 g; 4°C), and frozen at – 20°C. Pellets were resuspended in 500 µl of Z-Buffer (60 mM Na_2_HPO_4_, 40 mM NaH_2_PO_4_, 10 mM KCl, 1 mM MgSO_4_, pH 7.0) supplemented with 40 mM β-mercapthoethanol. Cells were then disrupted either mechanically by sonication for 3 × 20 s (Hielscher UP200S, 1 mm tip diameter probe, amplitude 50%) on ice with 1-min pauses in between or enzymatically with 10 µl of lysozyme (10 mg/ml, Serva) and 10 µl of 10% TritonX-100. The two approaches resulted in identical results with the same strains. 100 µl to 350 µl of sonicate/lysate (depending on the activity of the construct) was adjusted to 500 µl with Z-Buffer and pre-incubated for 5 min at 37°C. Reactions were started by the addition of 100 μl of orthonitrophenyl-β-D-galactopyranoside (ONPG; 4 mg/ml in Z-buffer, Sigma, N1127). Depending on the activity of the construct and development of the yellow color (no longer than 30 min), the reaction was stopped with 250 μl of 1 M Na_2_CO_3_. OD_420_ and OD_550_ values were measured, and β-galactosidase activity was calculated with the formula: activity$\ = \ 1000 \times ( {{\mathrm{OD}}420 - 1.75 \times {\mathrm{OD}}550} )/( {{\mathrm{V}} \times {\mathrm{T}} \times {\mathrm{c}}} )$, where V is sample volume [ml], T is time of the reaction [min], and c is protein concentration [mg/ml] measured by Bradford protein assay. Activity of an “empty” (*i.e*. promoterless) pDG3661 integrated into the BsB1 genome at *amyE* was used to determine the background. This background value was subtracted from the activities of the promoter constructs. Results from time zero are shown as Activity, results after 1 h of growth with or without RIF are shown as RIF-inducibility (RIF-treated/untreated). The experiments with each construct were conducted 2–5 x on different days. The exact numbers of replicates are indicated for each experiment.

### Protein purification

*Bacillus subtilis* RNAP containing a His_10_-tagged β’ [24] subunit was purified from LK1723 (wt strain) and LK1272 (strain without *helD*) as described [24]. Briefly, cells were grown to an OD_600_ ∼ 1.0 in 2 litres of LB media. Cells were then harvested by centrifugation (10 min; 6000 g; 4°C), and the pellet was resuspended in 1xP Buffer (300 mM NaCl, 50 mM Na_2_HPO_4_, 5% glycerol, 3 mM β-mercapthoethanol, pH 8.0). The cells were then disrupted by sonication for 20 × 10 s on ice with 1-min pauses (Hielscher UP200S, 14 mm tip diameter probe, amplitude 50%). The protein was purified from the soluble fraction by affinity chromatography using Ni-NTA Agarose (QIAGEN, 30 210). After elution with 400 mM imidazole in 1xP Buffer, fractions containing RNAP were pooled and dialyzed against storage buffer (50 mM Tris-HCl, pH 8.0, 100 mM NaCl, 3 mM β-mercapthoethanol, 50% glycerol), and stored at −20°C.

The σ^A^ subunit of RNAP (LK22) was overproduced and purified from inclusion bodies as described [25]. Briefly, cells were grown to OD_600_ ∼ 0.8 in 2 litres LB media and at this point expression of σ^A^ was induced with 0.8 mM IPTG for 3 h at room temperature. Cells were harvested by centrifugation (10 min; 6000 g; 4°C), and the pellet was resuspended in 10 ml of Lysis Buffer (40 mM Tris-HCl, pH 8.0, 300 mM KCl, 10 mM EDTA), and an additional 100 μg/ml of lysozyme was added to the lysate. The cells were then disrupted by sonication for 15 × 10 s on ice with 1-min pauses (Hielscher UP200S, 14 mm tip diameter probe, amplitude 50%), spun down at 4°C, and the pellet was resuspended in 10 ml Lysis Buffer with 2% sodium deoxycholate (Na-DOC) and again sonicated 4 × 30 s on ice with 1-min pauses. This was followed by centrifugation and resuspension in deionized water (2x). For solubilization of inclusion bodies, the pellet was resuspended in 10 ml of 6 M guanidine-HCl and allowed to sit for 30 min at 4°C. To refold the protein, Buffer A (50 mM Tris-HCl, 0.5 mM EDTA, 0.1 mM DTT, 5% glycerol) was added in two-fold dilution steps (up to 64x dilution; each step 30 min) at 4°C. The protein was then mixed with 1 g of Whatman DE52 cellulose dissolved in 10 ml of Buffer A. The mixture was mixed for 5 h at 4°C, and then the cellulose was allowed to gravity-sediment overnight. The excess buffer was removed, and the layer with cellulose was applied onto a Poly-Prep Chromatography column (BioRad) primed with a prior elution with Buffer A. The liquid was allowed to elute by gravity flow, and the column was then washed with 35 ml of Buffer A. The protein was eluted with an NaCl gradient (from 100 mM to 600 mM NaCl in 100 mM steps; each step was 3 × 1.5 ml) in Buffer A. The fractions were analyzed for protein content by the Bradford assay and checked by SDS-PAGE for protein purity. Fractions containing σ^A^ were then pooled and dialyzed against storage buffer (the same as for RNAP).

HelD (LK800) was prepared and purified as described [6]. Briefly, expression of HelD-His6 was induced with 1 mM IPTG for 2 h at room temperature. Cells were then harvested, and protein was purified by affinity chromatography as described for RNAP.

HelDΔN was prepared and purified as described in [26]. Briefly, HelDΔN expression was induced with 1 mM IPTG in *E. coli* Lemo21 (DE3) cells (New England Biolabs, Ipswich, MA, USA) grown in Power broth (Molecular Dimensions, Newmarket, UK). HelDΔN was then purified using Ni-NTA affinity chromatography (see RNAP purification) and size-exclusion chromatography.

Purity of all proteins was checked on SDS-PAGE (NuPAGE^TM^ 4–12% Bis-Tris Gel, Invitrogen, NP0321) with a Novex Sharp prestained protein standard as a marker (Invitrogen, LC5800).

### Preparation of DNA templates for *in vitro* transcription marker

Several templates were prepared to generate the marker: plasmid LK1834 bearing a *B. subtilis rrnJ* promoter fragment (PCR amplified from wt genomic DNA BsB1 (LK2711) with #2037 and #2039 cloned into pRLG770 via *EcoRI* and *HindIII*). Transcription (σ^A^-dependent) from this plasmid yields transcripts of 238 nt (from *rrnJ* P1), 153 nt (from *rrnJ* P2), and 108 nt (from P-RNA1, an integral part of the plasmid backbone required for its replication control). Furthermore, two fragments of the promoter (σ^A^-dependent) region of different lengths of the *B. subtilis hrc* gene were amplified from wt genomic DNA BsB1 (LK2711) with primers #2088+#2131 (transcript 80 nt) and #2088+#2089 (transcript 91 nt) by Expand High Fidelity PCR System (Roche, Cat No. 11 732 650 001). Purification of plasmids was performed with Promega Wizard Plus Midiprep DNA Purification System (A7640). The plasmids were then phenol-chloroform extracted and ethanol precipitated. PCR products were purified with QIAquick Gel Extraction Kit (QIAGEN, 28 706).

### Transcriptions *in vitro*

Transcription experiments were performed with the *B. subtilis* RNAP core (purified from LK1723) reconstituted with a saturating concentration of σ^A^ (LK22) (core:σ^A^ ratio 1:5) in storage buffer (50 mM Tris-HCl, pH 8.0, 0.1 M NaCl, 50% glycerol) for 30 min at 37°C. Multiple round transcription reactions were carried out in 10 μl reaction volumes in 1x transcription buffer contained 40 mM Tris-HCl, pH 8.0, 10 mM MgCl_2_, 1 mM dithiothreitol (DTT), 0.01 mg/ml BSA and 150 mM KCl (300 mM in the case of Fig. 4I, 4J), and all four NTPs (ATP, CTP, GTP – 200 μM each (for K_GTP_ constant determination, GTP was varied from 20 to 2000 μM), UTP – 10 μM plus 0.1 µl radiolabeled [α-^32^P]UTP (3 000 Ci/mmol, 10 mCi/ml, Hartmann Analytic, SRP-210). As a template, 30 ng supercoiled plasmid per 10 μl reaction was used unless stated otherwise. Rifampicin was included at specified concentrations where indicated. Transcriptions were initiated with 1 μl of 300 nM RNAP holoenzyme in most experiments. 1 μl of 3 μM RNAP holoenzyme was used for experiments shown in Fig. 4G-4J. For RNAP affinity for promoter DNA (closed complex formation), a serially diluted plasmid DNA containing the studied promoters (a range from 0.083 nM to 21.5 nM, final concentrations) was used. Transcriptions were allowed to proceed for 15 min at 37°C and then stopped with equal volumes of formamide stop solution (95% formamide, 20 mM EDTA, pH 8.0, 0.03% bromophenol blue, 0.03% xylene cyanol).

For *in vitro* transcriptions with HelD, HelDΔN and rifampicin, first RNAPΔHelD (purified from LK1272) and σ^A^ were reconstituted (core:σ^A^ ratio 1:5) in storage buffer (to yield a final holoenzyme concentration in 10 μl reaction 30 nM). These reconstitutions were allowed to proceed for 30 min at 37°C. Following this incubation, 30 ng of supercoiled plasmid DNA (LK1177) per 10 μl reaction was added to the reconstituted proteins and incubated for another 10 min at 37°C. Then, rifampicin was added at final concentrations of 0.023 μg/ml, 0.046 μg/ml, or no rifampicin (Sigma R3501, diluted in DMSO), and the reaction mixture was incubated for 10 min at 37°C. Finally, HelD or HelDΔN was added or not at a final concentration of 480 nM in the 10 μl reaction volume, followed by another 10 min incubation at 37°C. The volume of each sample at this point was 4 μl. A separate mixture (6 μl per reaction) was prepared containing transcription buffer, BSA, and KCl (1x, 0.01 mg/ml, and 150 mM in 10 μl) plus NTPs (concentrations as in multiple rounds of transcriptions). This mixture was then allowed to equilibrate at 37°C for 5 min. Transcriptions were then initiated by adding 6 μl of the NTP mixture to 4 μl of the reconstituted proteins and DNA template ± rifampicin (0.023 and 0.046 μg/ml final concentration, respectively), allowed to proceed for 15 min at 37°C, and terminated with equal amounts of stop solution.

Single-round transcriptions were performed to study promoter escape. Reactions were carried out in 10 μl: 30 ng of supercoiled DNA templates (LK2997, LK3610, LK1177), transcription buffer with the same composition as described before. First, RNAP and σ^A^ were reconstituted in storage buffer (final concentration 30 nM RNAP, 150 nM σ^A^) at 37°C, 10 min. Transcriptions were started by the addition of NTPs (concentration of NTPs was the same as in multiple rounds of *in vitro* transcription assays) together with 600 nM double-stranded (dsDNA) competitor that allows only one round of transcription by sequestering free RNAP [13]. Stock dsDNA competitor was prepared by annealing equimolar amounts of complementary primers (#923 + #924) in 100 mM Tris-HCl, pH 8.0, 10 mM EDTA, 500 mM KCl. From the master reaction, aliquots were withdrawn at the indicated time points and added to the stop solution. In competitor test controls (to establish that the amount of the competitor used is sufficient to block transcription if added prior to RNAP), the whole reaction mix with plasmid DNA and competitor dsDNA was started with RNAP.

To resolve the products of transcriptions, aliquots (10 μl) from transcription reactions were loaded on 7 M urea 7% polyacrylamide 1xTBE gels and electrophoresed in 1xTBE (90 mM Tris-Cl, pH 8.0, 90 mM boric acid, 2 mM EDTA, pH 8.0). Gels were run for 105 min at 180 V. Gels were dried for 1 h at 80°C, cooled down, and exposed overnight on BAS storage phosphor screen (Fujifilm). Subsequently, the screen was scanned using Amersham™ Typhoon™ 5 Biomolecular Imager (Cytiva) with a phosphor imaging emission filter 390BP. The signal was quantified with the QuantityOne (Bio-Rad, version 4.6.3) software, the background was subtracted, and the resulting values were plotted using SigmaPlot (version 8.0). Statistical calculations were done in Microsoft Excel (Office 365, version 24–25).

For relative RNAP affinity for promoter DNA (closed complex formation), the data were fitted to the equation $f = a \times [ {1 - {\mathrm{exp}}( { - b \times x} )} ]$ (where f is the relative transcription, x is the concentration, and a and b are constants) [27].

For RNAP sensitivity to the iGTP (K_GTP_ – an indirect measure of open complex stability), the data were fitted to the equation $f = a \times [ {1 - {\mathrm{exp}}( { - b \times x} )} ]$ (where f is the relative transcription, x is the concentration, and a and b are constants) [27].

To determine the average time of promoter escape, the data were fitted to the equation $f = a \times [ {1 - {\mathrm{exp}}( { - b \times x} )} ]$ (where f is the relative transcription, x is the concentration, and a and b are constants).

To generate the molecular size RNA marker, the same conditions were used as described above, with the following modifications. For transcription, *E. coli* RNAPσ^70^ holoenzyme (efficiently recognizes *B. subtilis* promoters; [28]) was used (New England BioLabs, M0551), and the templates were 60 ng DNA fragments of the *hrc* gene and 100 ng of plasmid. These templates were combined in one reaction and yielded a ladder of 80, 91, 108, 153, and 238 nt (see Supplementary Fig. S3 for an example).

### Preparation of radiolabeled RNA probes for the detection of helD/ashelD

Probes for Northern blots used in this study were generated by *in vitro* transcription using T7 RNAP (Thermo Fisher Scientific). DNA templates containing Class I T7 promoter were produced by PCR [primers #5239+#5240 for anti-*helD* probe, gDNA from BsB1 (LK2711) was used as the template; primers #5285+#5286 for anti-as*helD* probe, synthetic oligonucleotide (LK5284) was used as the template]. The PCR products were purified by PCR purification kit (Qiagen) according to the manufacturer’s instructions. *In vitro* transcription reactions were carried out in 100 μl reactions in T7 transcription buffer containing 40 mM Tris–HCl (pH 7.9), 10 mM MgCl_2_ and 5 mM DTT, 1 mM each of ATP, CTP, GTP, 0.1 mM UTP complemented with 3 μl of α[^32^P] UTP (3 000 Ci/mmol, 10 mCi/ml, Hartmann Analytic, SRP-210), 5 μl T7 RNA polymerase and 1 μg of DNA template. RNA was DNAsed by DNAse I (TURBO DNA-free Kit, Ambion) and purified using Micro Bio-Spin™*P* -6 Gel Columns (Biorad) according to the manufacturer’s instructions. The purified RNA was then analyzed on a denaturing polyacrylamide gel to confirm the expected size of the RNA products. The probe was denatured (95°C for 5 min) before use.

### Northern blotting for detection of helD/ashelD

5 μg of DNAsed RNA samples (the same as used for RT-qPCR, see above) were precipitated, resuspended in 6 μl of 1x RNA loading dye (identical with stop solution used in *in vitro* transcriptions) and denatured (95°C for 5 min). To detect the full-length helD mRNA, samples were run on a 1.5% agarose gel containing 1.8% formaldehyde in 1x MOPS buffer (20 mM 3-(N-morpholino) propanesulfonic acid, pH 7.0, 5 mM sodium acetate, 1 mM EDTA). To detect as*helD*, the samples were run on 7% PAA gels in 7.5 M urea, 1xTBE buffer. RNA from both types of gels was transferred onto a BrightStar™-Plus Positively Charged Nylon Membrane (Invitrogen) using a semidry transfer apparatus at 15 V for 1 h (Bio-Rad). After crosslinking by UV, the membrane was stained by Methylene Blue (0.04% Methylene Blue in 0.5 M sodium acetate, pH 5.2) for nucleic acid visualization. The membrane was then prehybridized in HYBE buffer [1% SDS, 6x SSPE (Invitrogen), 10x Denhardt’s Solution (Invitrogen), 50% formamide, 0.2 mg /ml salmon sperm DNA (Invitrogen)] for 1 h at 55°C and hybridized with a respective radiolabeled RNA probe overnight at 55°C. The membrane was washed in three steps at 65°C: (i) 2x SSC buffer (Thermo Fisher Scientific) supplemented with 0.1% SDS, (ii) 2x SSC buffer supplemented with 1% SDS, and finally (iii) in 0.1x SSC. The membrane was exposed to a storage phosphor screen (Fujifilm) and scanned by a radio imaging system (AmershamTyphoon).

### Statistics

Statistical calculations were done in Microsoft Excel (Office 365, version 24–25) with the two-tailed, unpaired *t*-test and the following equation = T.TEST (first data set; second data set; 2; 3) where 2 indicated a two-tailed distribution, and three indicated two-sample unequal variance.

### Bioinformatic search for convergent promoters in *B. subtilis* potentially responsive to rifampicin

To find other candidate two-promoter systems we defined a degenerate promoter motif TTGN(3)N(17,18)[AT](6)N(45,55)[AT](6)N(18,19)N(3)CAA based on the discovered promoter sequences experimentally validated upstream of *helD* and used fuzznuc from EMBOSS [29] package to find the motif in the *B. subtilis* 168 genome (accession NC_000964.3). We further filtered out sites that only occurred in 300 bp upstream regions of genes encoding proteins seen upregulated after treatment with rifampicin.

Data and code for the analysis are deposited at the Zenodo archive [https://doi.org/10.5281/zenodo.15784301] in “held_emboss_search”.

### Two-promoter system occurrence among bacteria

We have obtained complete bacterial assemblies from RefSeq [30] on 23.5.2025 with NCBI datasets [31] excluding atypical assemblies. Based on the known sequences of the two-promoter system in upstream regions of *pps* and *helD* genes, we defined individual -35 and -10 motifs for sense and antisense promoters. These individual motifs were searched for with FIMO [32] in the obtained genome assemblies (*p*-value threshold of 0.05). Only combinations of individual motif sites for + and − promoters -35 and -10 regions that satisfied the following conditions were retained:

1. The distance between the -35 and -10 motif sites was 16–20 bp.
2. The distance between promoters in the + and − strands was 45–100 bp.
3. The sum of scores reported by FIMO for the site combination was greater than or equal to 30.
4. Further, we filtered for such two-promoter systems that were located in a 300 bp upstream region of the annotated region, and that did not overlap with any other annotated region.

The result of the FIMO search and subsequent filtering steps is included in Supplementary Table S3. All FIMO searches were conducted with a background Markov model computed from the corresponding genome assembly with the fasta-get-markov program from MEME suite [33].

To visualize the distribution of the two-promoter system among organisms, we used the pruned NCBI taxonomy [34] tree at the “family” level and added “unassigned” leaf where the assembly family was not assigned. The tree editing was done with the ETE 3 library [35], and the tree was visualized with the iTOL web service [36].

The search revealed a substantial number of two-promoter system sites with associated downstream protein. We clustered the obtained proteins to find out what kind of proteins are mostly represented. The clustering was performed using MMseqs2 [37] easy-cluster pipeline (–min-seq-id 0.1 –cov-mode 0 -c 0.5 –cluster-reassign, Supplementary Table S4).

Data and code for the analysis are deposited at the Zenodo archive [https://doi.org/10.5281/zenodo.15784301].

### Gene ontology analysis

To further explore proteins that were found downstream of two-promoter system sites, we’ve mapped the gene ontology (GO) terms associated with identified proteins to GO slim prokaryote (release 2025–03-16) gene ontology [38] subset with goatools [39]. We then counted the per-protein weighted occurrences of the GO slim terms and plotted the four most commonly seen terms in a pie chart with pandas [40] and matplotlib [41] libraries.

Data and code for the analysis are deposited at the Zenodo archive [https://doi.org/10.5281/zenodo.15784301].

## Results

### HelD and Pps are the most enriched proteins after treatment with sub-MIC RIF

To start defining the RIF resistome and its expression control in *B. subtilis*, we performed proteome analysis of cells exposed to a sub-inhibitory (sub-MIC) concentration of RIF compared to non-treated cells (Fig. 1A). This approach identified >10 significantly upregulated proteins (Supplementary Table S5). The two most enriched proteins were HelD (UniProt No. O32215) and Pps (UniProt No. O34309). These two proteins and the regulation of their expression were selected for the following study. Pps is predicted to be a homolog of rifampicin phosphotransferase (alternative name RPH), which has not been studied in *B. subtilis* yet. However, the 3D structure of RPH (Pps) from *Listeria monocytogenes* (*Lm*) has been solved [42, 43]. RPH-*Lm* phosphorylates RIF at C(21), abolishing its binding to RNAP [42].

### Deletion of *helD* results in increased sensitivity to RIF

As *Bsu*_HelD had been originally believed not to be involved in RIF resistance [7], we primarily focused on this protein to address this discrepancy. We created knock-out, complementation, and merodiploid strains. The merodiploid overexpressed *helD* approximately two-fold (Supplementary Fig. S4). In the absence of RIF, the strains displayed about the same growth as wt, both on solid and in liquid media (Fig. 1B, 1D). In the presence of RIF, the Δ*helD* strain displayed dose-dependent growth [44] in liquid media, manifested by prolonged lag-phase, and slow growth phenotype on solid media (Fig. 1C, 1E). This phenotype was partially reverted in the complementation strain, likely due to a sub-wt level (∼60%) of the *helD* transcript in this strain (Supplementary Fig. S4). The merodiploid strain then behaved as wt. We obtained similar results when determining the MIC. The knock-out strain had increased sensitivity to rifampicin (Supplementary Fig. S5). Taken together, the absence of the *helD* gene renders the bacterium more sensitive to RIF.

Finally, pull-down experiments revealed that after the sub-MIC RIF treatment, not only did the HelD protein level increase (Fig. 1A), but also its association with RNAP increased (Fig. 1F).

### HelD N-terminal domain mediates RIF resistance

**Figure 2.**
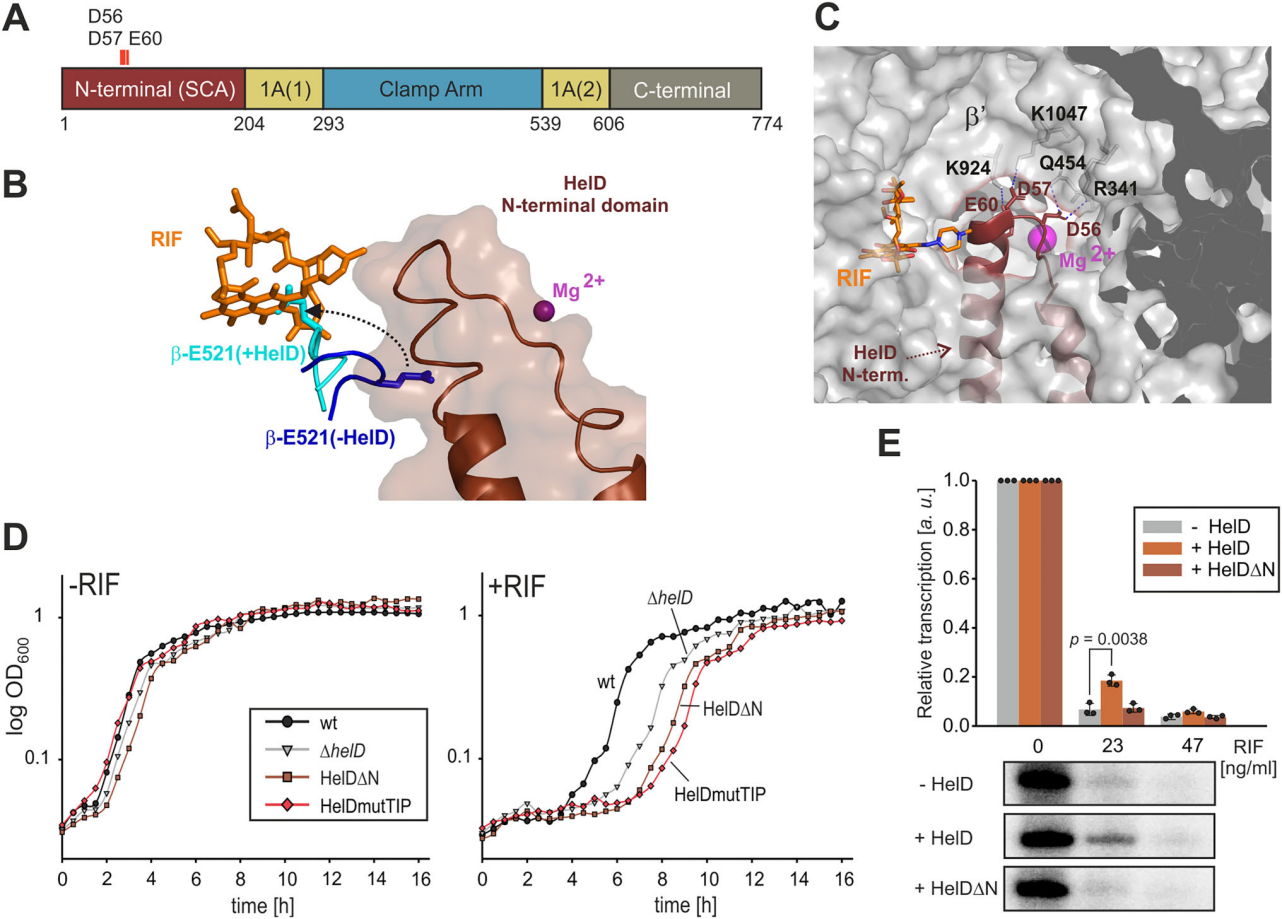
**Role of**  ***Bsu_***  **HelD N-terminal domain in RIF resistance. (A)** A scheme of *Bsu_*HelD domain organization with acidic aa in the N-terminal domain is indicated. The N-terminal domain [also known as secondary channel arm (SCA)] is indicated and colored ruby-brown. Amino acid residues at the domain borders are indicated. **(B)** A structural analysis of *B. subtilis* RNAP showing deformation of the expected RIF binding pocket in the presence of HelD. Teal, RNAP β-E521 (+HelD); dark blue, RNAP β-E521 (-HelD); ruby brown, semitransparent surface of the HelD N-terminal domain with the peptide backbone indicated within; orange, RIF; magenta, catalytic Mg^2+^.The black arrow shows repositioning of β-E521 forced by the HelD N-terminal domain. The model is based on the superposition of structures aligned by the β subunit: *Bsu* RNAP elongation complex (PDB ID: 6WVJ,[4]), *Bsu* RNAP with HelD (PDB ID: 6ZFB,[3–5]). The expected position of rifampicin is adopted from the *Mycobacterium tuberculosis* (*Mtb*) RNAP complex (PDB ID: 5UHC,[10]). **(C)** A close-up of the wt *Bsu_*HelD N-terminal domain tip interacting with *Bsu* RNAP (PDB ID: 6WVK,[4]) with the three acidic residues and their interactions with RNAP indicated with blue dashed lines. RNAP is represented as a semitransparent grey surface. The Mg^2+^ ion in the RNAP active site is shown as the magenta sphere. The N-terminal domain of HelD is shown in ruby brown, secondary structure representation with relevant aa residues shown as sticks. RNAP is shown as a grey surface. Their interaction partners (in the case of wt) from the β’ domain are shown as grey sticks and marked. The approximate position of RIF (carbon in orange, represented as sticks and marked) in the active site is adopted from the complex with *Mtb* RNAP (PDB ID: 5UHC,[10]). The graphics were created using PyMOL. **(D)** Growth of indicated strains in liquid LB medium in the absence (-RIF) or presence (+RIF) of sub-MIC concentration (0.03 μg/ml) of RIF. The strains were inoculated in a 24-well plate, and the OD_600_ was measured every 30 min for ∼ 16 h. A representative result from three growth experiments is shown. Black dots, wt (LK2711); grey triangles, Δ*helD* (LK2840); brown squares, HelDΔN (HelD lacking the N-terminal domain; LK3772); red diamonds, HelDmutTIP (D56A, D57A, E60A; LK3784). **(E)** Multiple round transcriptions from the P*veg* (LK1177) promoter were performed in the absence or presence of HelD or HelDΔN with increasing amounts of RIF. The 1:16 RNAPΔHelD:HelD or RNAPΔHelD:HelDΔN ratio was used in protein reconstitution. Transcription at zero RIF was set as 1 for both -/+ HelD/HelDΔN to facilitate visualization of the changes. The bars show averages of three independent experiments, the dots are individual experimental data, and the error bars show $ \pm $ SD. *p*-values were calculated using a two-tailed, unpaired *t*-test and indicated in the graph – this is also used in subsequent figures. Primary data are shown below the graph.

Next, we investigated which part of HelD is important for its anti-RIF effect. In a review published recently [45], it was speculated that *Bsu_*HelD might provide some protection against RIF, and this effect might be mediated by the N-terminal domain of *Bsu_*HelD (Fig. 2A) as it reaches close to the vicinity of the RIF binding pocket. Accordingly, structural analysis and superposition of available structures supported this hypothesis when the presence of the N-terminal domain of *Bsu_*HelD deformed the expected RIF binding pocket by β-E521 intruding into the space occupied by RIF (Fig. 2B and Supplementary Fig. S6).

To test this model, we created a strain lacking the N-terminal domain of HelD (HelDΔN), and a strain with three acidic amino acids (aa) at the tip of the N-terminal domain mutated to Ala residues (HelDmutTIP: D56A, D57A, E60A). The acidic aa had been predicted by an *in silico* analysis to stabilize the interaction between the HelD N-terminal tip and positive residues of β’ (Fig. 2C).

We then compared the growth of the two mutants in liquid media with wt and Δ*helD* in the absence/presence of RIF (Fig. 2D). Both new mutant strains displayed the prolonged lag-phase phenotype.

To reveal whether *Bsu_*HelD directly protects RNAP against RIF, we performed transcriptions *in vitro* -RIF/+RIF, using a strong σ^A^-dependent promoter, P*veg* [28]. The transcriptions were done either in the absence of HelD or in the presence of the intact protein or its variant lacking the N-terminal domain (Fig. 2E). The addition of RIF decreased transcription in all cases. However, in the presence of HelD, the decrease was less pronounced, indicating that HelD protects RNAP against RIF, thereby diminishing the antibiotic’s inhibitory effect on the enzyme. HelDΔN had no protective effect. Collectively, these results demonstrate that Class I HelD proteins also provide the cell with a defensive mechanism against RIF.

### HelD expression is induced by RIF transcriptionally

**Figure 3.**
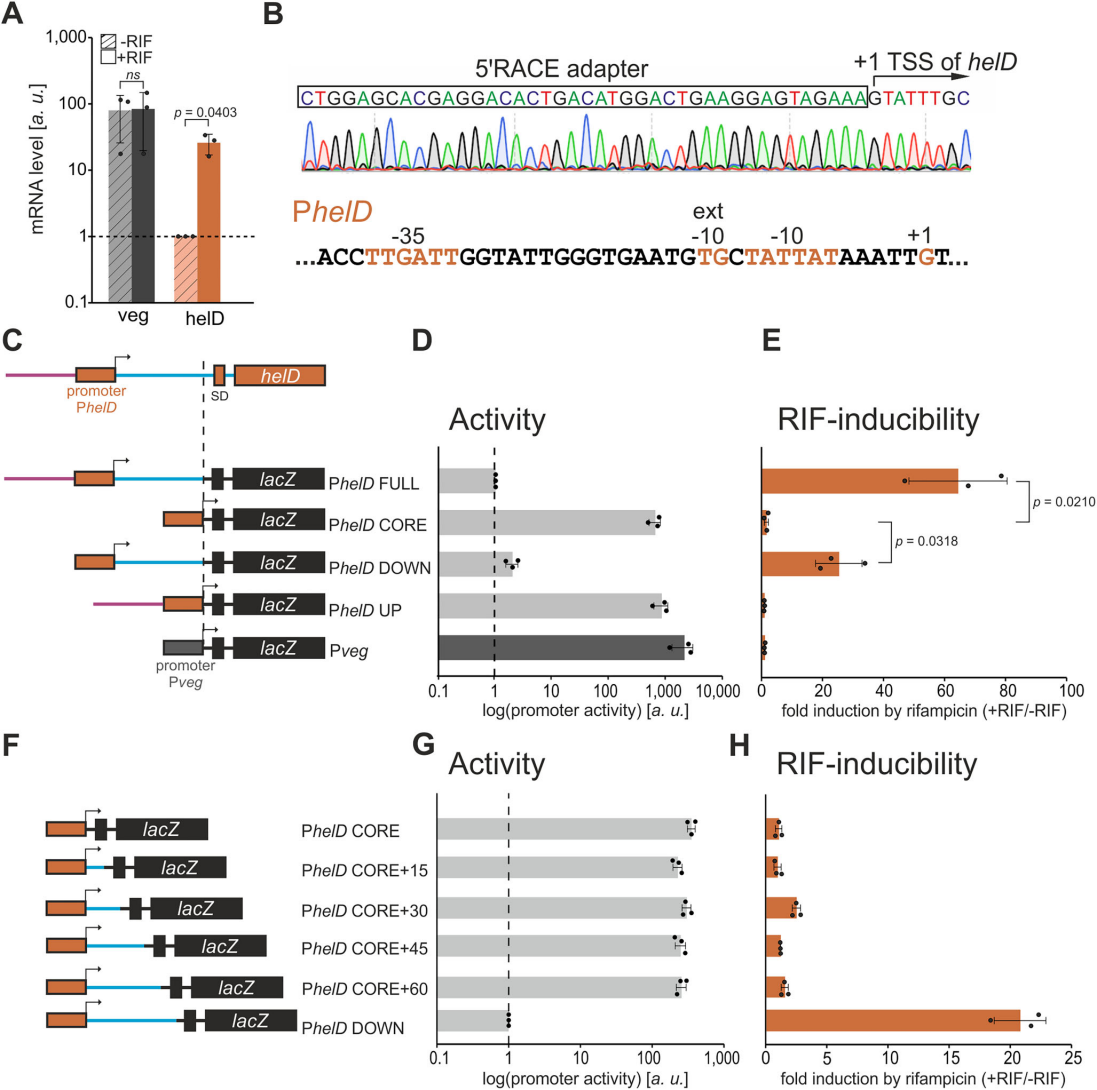
**Regulation of *helD* expression by RIF. (A)** Relative levels of *helD* and *veg* mRNAs determined with RT-qPCR in wt (LK2711). The relative *helD* mRNA level in the absence of RIF (-RIF) was set as 1. The graph (y-axis, log scale) shows averages from three independent experiments, the dots are individual experimental data, the error bars show $ \pm $ SD. “*ns*” indicates non-significance, *p* > 0.05. **(B)** Determination of TSS for the *helD* gene with 5′RACE (top) and the sequence of the *helD* promoter (P*helD*, bottom). **(C)** A scheme of promoter-*lacZ* fusions was created with DNA fragments from the *helD* gene upstream region (shown on top). The dashed line indicates the downstream end of the fusions. P*helD* FULL (LK3005), P*helD* CORE (LK2970), P*helD* DOWN (LK3004), P*helD* UP (LK3038), and control P*veg* CORE (LK3040). For the sequence of these and subsequent constructs, see Supplementary Fig. S8. **(D)** The graph shows relative activities (arbitrary units, *a. u*.) of promoter-*lacZ* fusions [from **(C)**] in exponential phase (OD_600 _= 0.5) in the absence of RIF. The strong P*veg* CORE promoter was used as a control. Activity of P*helD* FULL was set as 1. The bars are averages from three independent experiments, the dots are individual experimental data, and the error bars show $ \pm $ SD. **(E)** The graph shows inducibility of promoter-*lacZ* fusions [from **(C)**] by RIF, expressed as activity in the presence of RIF to activity in its absence (+RIF/-RIF). The activity without RIF was set as 1 for each construct. The bars are averages from three independent experiments, the dots are individual experimental data, and the error bars show $ \pm $ SD. **(F)** A scheme of constructs created by successive extensions of the P*helD* CORE promoter by 15 nt. P*helD* CORE (LK2970), P*helD* CORE+15 (LK3117), P*helD* CORE+30 (LK3118), P*helD* CORE+45 (LK3119), P*helD* CORE+60 (LK3120) and P*helD* DOWN (LK3004). **(G)** The graph shows activities of extended constructs from **(F)**. The bars are averages from three independent experiments (*a. u*.), the dots are individual experimental data, and the error bars show $ \pm $ SD. **(H)** Inducibility of constructs [from **(F)**] after RIF treatment (+RIF/-RIF). The activity without RIF was set as 1 for each construct. The bars are averages from three independent experiments, the dots are individual experimental data, and the error bars show $ \pm $ SD.

The key question then was – how does RIF induce HelD expression? First, we examined whether this induction was primarily at the mRNA or protein level. We cultivated wt *B. subtilis* cells and performed the same treatment (-RIF/+RIF) as for the proteome analysis. We then determined by RT-qPCR the relative levels of *helD* and *veg* (control) mRNAs. Surprisingly, after the sub-MIC RIF treatment, the relative amount of *helD* mRNA increased ∼25-fold while the level of *veg* mRNA remained unchanged (Fig. 3A), revealing that the induction was already at the *helD* mRNA level. Two hypotheses could be postulated to explain this induction: (a) *helD* mRNA synthesis increases in response to RIF; (b) RIF inhibits an RNase that is specific for *helD* mRNA.

### *helD* upstream region contains a RIF-responsive sequence

To distinguish between the two hypotheses and to start unravelling the mechanism of *helD* expression, we first identified the *helD* promoter. Using 5′RACE, we determined the transcription start site (TSS) of *helD*, 98 nucleotides upstream of the translational start (Fig. 3B). The TSS was the same regardless of the presence/absence of RIF (data not shown).

Next, we created a construct containing the full *helD* gene upstream region, named P*helD* FULL, consisting of the P*helD* core promoter (CORE) plus its upstream (UP) and downstream (DOWN) regions. We fused this region to the marker *lacZ* gene (Fig. 3C) and integrated it into the *B. subtilis* chromosome. Expression of *lacZ* then served both as a measure of promoter activity and to distinguish between hypotheses (a) and (b). If there were a specific RNase targeting *helD* mRNA, we would not expect its effects to be reproduced with the *lacZ* mRNA. We then performed β-galactosidase assays to determine promoter activity in the absence and presence of various concentrations of RIF (comparing activity after 60 min ± RIF) to identify the conditions where RIF would have the most pronounced effect. The results revealed that the promoter activity of this construct increased in a RIF-dependent manner more than an order of magnitude and was sensitive to sub-MIC levels of RIF (Supplementary Fig. S7A). This excluded hypothesis (b) and suggested that this region contains regulatory elements for the stimulation of transcription in the presence of RIF.

To identify these elements, we created additional *lacZ* fusions containing either the P*helD* core promoter (CORE) alone or in combination with its UP or DOWN regions. As a control, we used a P*veg-lacZ* fusion (Fig. 3C). Using the RIF concentration that had elicited the highest relative induction of promoter activity (Supplementary Fig. S7A), we determined promoter activities of these constructs. Fig. 3D shows relative promoter activity in the absence of RIF, and Fig. 3E shows RIF inducibility (activity +RIF/activity -RIF).

Consistent with the previous experiment, the FULL construct showed low activity in the absence of RIF and was highly induced by RIF. A similar trend was observed with the DOWN construct. Both CORE and UP constructs were highly active even without RIF, and after the RIF treatment, no induction was observed (Fig. 3D and E). The same results were observed with the same promoter-*lacZ* fusions in a Δ*helD* background. This excluded a role of HelD in the regulation of its own expression (Supplementary Fig. S7B and C).

To identify the minimal sequence in the DOWN region responsible for the RIF-dependent regulation, we created new constructs by sequentially expanding the CORE promoter (CORE+15, CORE+30, CORE+45, and CORE+60; Fig. 3F, for sequences see Supplementary Fig. S8). All the new constructs behaved as CORE – they were highly active and not RIF-inducible. The only inducible construct was DOWN (Fig. 3G, 3H).

### *helD* upstream region contains two convergent promoters

**Figure 4.**
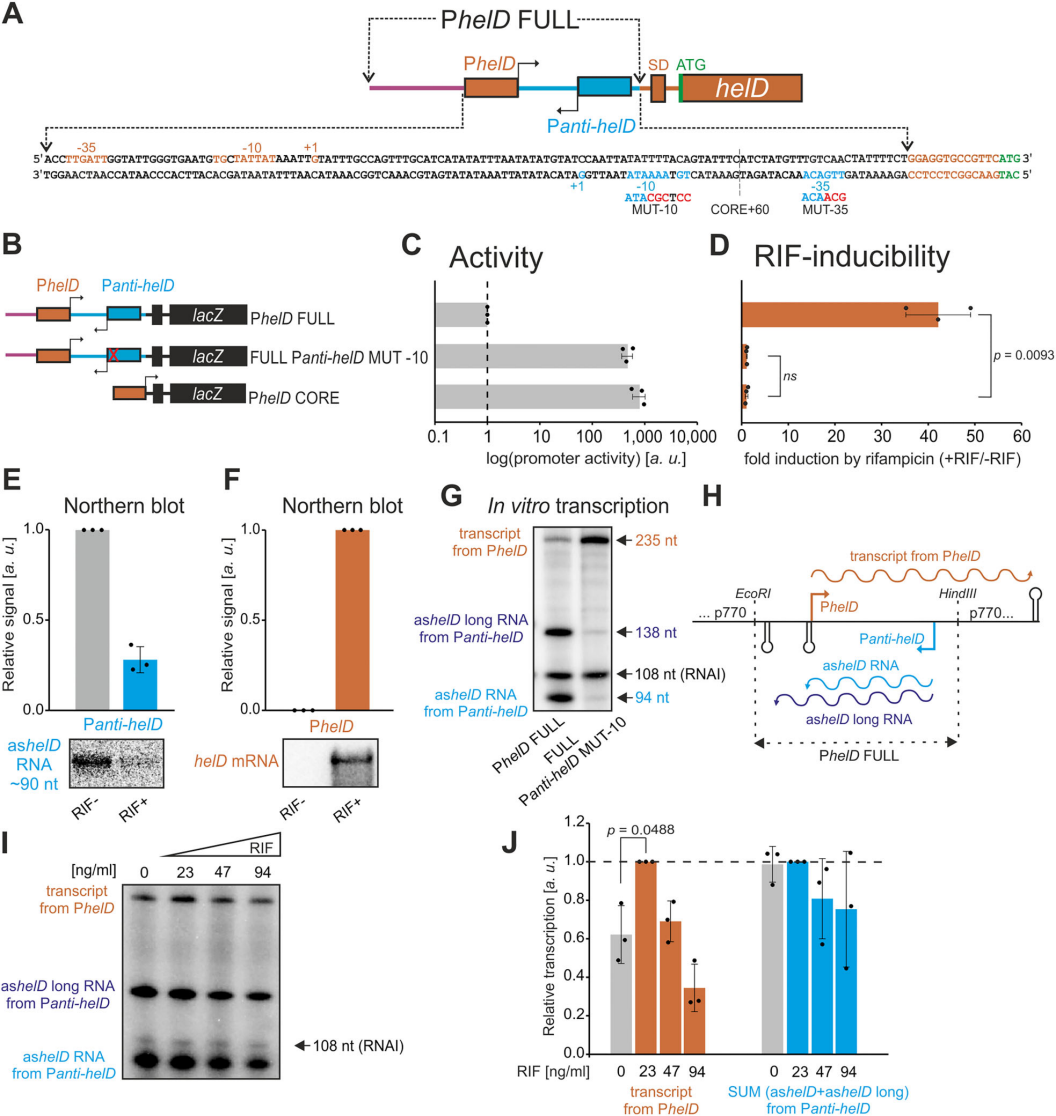
**Interplay between two convergent promoters leads to P*helD* inducibility by RIF. (A)** Sequence of the *helD* upstream region with indicated P*helD* and P*anti-helD* promoters, Shine-Dalgarno (SD, ribosomal binding site), and start codon (ATG, green). Mutations of the P*anti-helD* promoter are also indicated. P*helD* (sense strand) is highlighted with dark orange, P*anti-helD* (antisense strand) in blue, and mutations in -10 and -35 promoter elements are in red. **(B)** A scheme of P*helD* FULL (LK3005), FULL P*anti-helD* MUT -10 (LK3234), and P*helD* CORE (LK2970) promoter-*lacZ* constructs. **(C)** Activities of P*helD* FULL, FULL P*anti-helD* MUT -10 and P*helD* CORE promoter-*lacZ* constructs [from **(B)**] in exponential phase (OD_600 _= 0.5) in the absence of RIF. Activity of P*helD* FULL was set as 1. The data shows averages from three independent experiments; the dots are individual experimental data, and the error bars show $ \pm $ SD. **(D)** Inducibility of constructs from **(B)** (+RIF/-RIF). Activity without RIF for each construct was set as 1. The bars are averages from three independent experiments, the dots are individual experimental data, and the error bars show $ \pm $ SD. **(E)** Northern blot analysis of transcription from P*anti-helD* (as*helD* RNA) in the absence/presence of RIF. The bars are averages from three independent experiments, the dots are individual experimental data, and the error bars show $ \pm $ SD. Representative primary data are below the graph. **(F)** Northern blot analysis of transcription from P*helD* (*helD* mRNA). The bars are averages from three independent experiments, and the dots are individual experimental data. Primary data are shown below the graph. **(G)**  *In vitro* multiple round transcriptions from the P*helD* FULL (LK2994) and FULL P*anti-helD* MUT -10 (LK3229) constructs were performed in the absence of RIF. A representative result is shown from a total of four independent transcription experiments. **(H)** Schematic representation of three different transcripts generated from the P*helD* FULL construct in pRLG770. **(I)**  *In vitro* multiple-round transcriptions from the P*helD* FULL (LK2994) construct were performed with increasing amounts of RIF. A representative gel is shown. The identity of the transcripts is color-coded as in **(G)** and **(H). (J)** Quantitation of results from **(I)** plus two other independent experiments. Transcription with 23 ng/ml RIF was set as 1 for both P*helD* and the combined value of as*helD* long plus as*helD* (SUM *Panti-helD*). The signal was normalized to the number of uridines (bearing the radio-label) in each type of transcript. The bars show averages of three independent experiments, the dots are individual experimental data, and the error bars show $ \pm $ SD.

The difference between CORE+60 and DOWN was 24 bp, where we identified a putative -35 region of an unknown promoter in the opposite, antisense direction. Spaced by 19 bp, a matching -10 region including the extended -10 sequence [5′-TGTAAAATA-3′] was located. We note that an alternative -10 hexamer [5′-TAAAAT-3′] can be found with an 18 bp spacer; in this case, however, the extended -10 element is not appropriately spaced. We named this putative promoter P*anti-helD* (Fig. 4A). To test whether P*anti-helD* is important for the regulation, we mutated the -10 region of P*anti-helD* and compared the activity/RIF-inducibility of FULL P*anti-helD* MUT -10 with P*helD* FULL in β-galactosidase assays (Fig. 4B). Indeed, as the P*anti-helD* was abolished, the FULL P*anti-helD* MUT -10 construct became highly active even without RIF induction; RIF-inducibility was lost (Fig. 4C, 4D). Similar, but less prominent results we observed also with the mutation of the -35 region of P*anti-helD* (FULL P*anti-helD* MUT -35) (Supplementary Fig. S7D-S7F). Database mining then revealed that the existence of an antisense promoter was consistent with a study by Forrest *et al.* [46] where they had mapped TSS in *B. subtilis* and where a TSS in the *helD* 5′ untranslated region (5′ UTR) had been found in the antisense orientation.

We then characterized the antisense transcript from P*anti-helD* and its accumulation in dependence on ± RIF by Northern blotting. We detected a single antisense transcript (as*helD* RNA) of ∼90 nt in the absence of RIF (Fig. 4E, for the full gel see Supplementary Fig. S9). The level of this transcript decreased in the presence of RIF. The opposite behavior was observed for the *helD* mRNA (Fig. 4F, for the full gel see Supplementary Fig. S10), a single gene transcription unit [47].

Subsequently, *in vitro* transcriptions then revealed that the P*helD* FULL construct yielded a transcript originating from P*helD* (∼235 nt) and two transcripts of different lengths [∼94 nt (as*helD* RNA) and ∼138 nt (as*helD* long RNA), respectively] from P*anti-helD* that were mostly abolished when FULL P*anti-helD* MUT -10 was the template (Fig. 4G). The shorter of the two antisense transcripts was of the same length as the transcript detected *in vivo* (Supplementary Fig. S9). An analysis of the region downstream of P*anti-helD* revealed two putative intrinsic terminators (Supplementary Fig. S11). The absence of the longer transcript from P*anti-helD in vivo* then suggested that termination at the more proximal terminator was already sufficiently effective in the cell, not allowing detectable amounts of RNAP to reach the second terminator. This P*anti-helD* more distal terminator likely functions as the terminator from the opposite direction for the *helD* upstream gene, *yvgT*, which encodes a potential TRIC transporter [48]. A scheme showing the *in vitro* transcripts and their orientation is shown in Fig. 4H.

Finally, the -10 mutation in P*anti-helD* increased transcription from P*helD* also *in vitro* (Fig. 4G).

### P*helD* and P*anti-helD* react differently to RIF

To gain insights into the mechanistic aspects of the regulation by RIF, we performed transcriptions *in vitro* -RIF/+RIF using the P*helD* FULL construct and quantified the amounts of transcripts from individual promoters. Fig. 4I, 4J shows that RIF, in a concentration-dependent manner, increased transcription from P*helD* while transcription from P*anti-helD* did not display such an increase. Although the RIF-inducibility of the P*helD* transcript was not as pronounced as *in vivo*, the experiment proved that the key elements required for the regulation were present in the minimal *in vitro* system. Moreover, a control experiment using P*helD* CORE and P*anti-helD* CORE showed that these two promoters exhibited no differential sensitivity to RIF when tested separately (Supplementary Fig. S12, for sequences see Supplementary Fig. S13).

### RIF-inducibility depends on the interplay between convergent promoters with different properties

**Figure 5.**
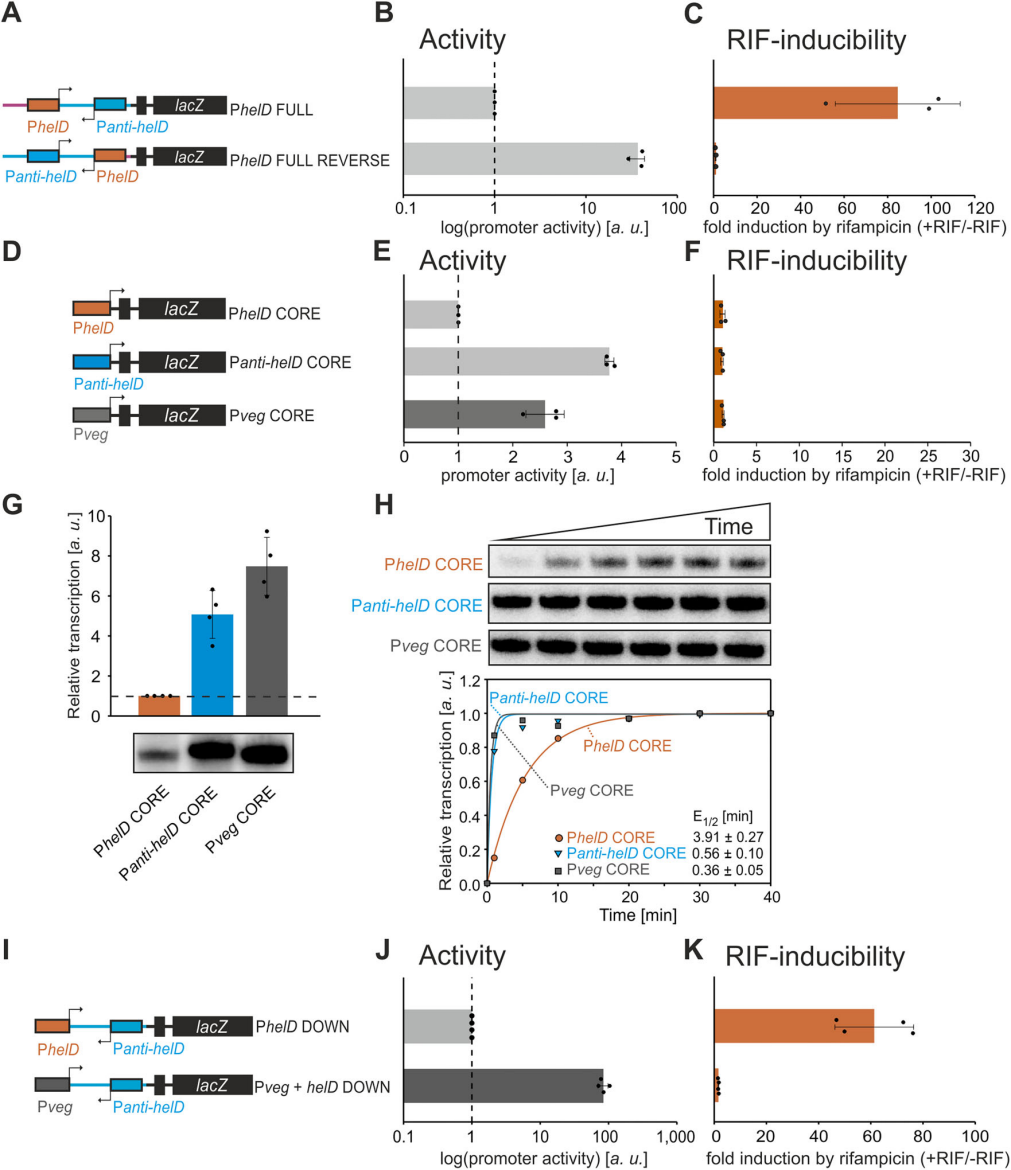
**Properties of P*helD* and P*anti-helD* determine expression control of *helD* by RIF. (A)** A scheme of P*helD* FULL (LK3005) and P*helD* FULL REVERSE (LK3826) promoter-*lacZ* constructs (in the latter construct, the positions of P*helD* and P*anti-helD* were swapped). **(B)** Activities of P*helD* FULL and P*helD* FULL REVERSE promoter-*lacZ* constructs [from **(A)**] in the exponential phase (OD_600 _= 0.5) in the absence of RIF. Activity of P*helD* FULL was set as 1. The bars are averages from three independent experiments, the dots are individual experimental data, and the error bars show $ \pm $ SD. **(C)** Inducibility of constructs from **(A)** (+RIF/-RIF). Activity without RIF for each construct was set as 1. The bars are averages from three independent experiments, the dots are individual experimental data, and the error bars show $ \pm $ SD. **(D)** A scheme of the CORE promoter-*lacZ* constructs P*helD* (LK2970), P*anti-helD* (LK3614), and P*veg* (LK3040). **(E)** Activities of P*helD* CORE, P*anti-helD* CORE, P*veg* CORE promoter-*lacZ* constructs [from **(D)**] in exponential phase (OD_600 _= 0.5) in the absence of RIF. Activity of P*helD* CORE was set as 1. The bars are averages from three independent experiments, the dots are individual experimental data, and the error bars show $ \pm $ SD. **(F)** Inducibility of constructs from **(D)** (+RIF/-RIF). Activity without RIF for each construct was set as 1. The bars are averages from three independent experiments, the dots are individual experimental data, and the error bars show $ \pm $ SD. **(G)**  *In vitro* multiple round transcriptions from P*helD* CORE (LK2977), P*anti-helD* CORE (LK3610), and P*veg* CORE (LK1177) promoters. Transcription of P*helD* CORE was set as 1. The bars show averages of four independent experiments, the dots are individual experimental data, and the error bars show $ \pm $ SD. Primary data are shown below the graph. **(H)** Single round transcription assay for determination of the promoter escape half-life (E_1/2_) from the P*helD* CORE (LK2977), P*anti-helD* CORE (LK3610), and P*veg* CORE (LK1177) promoters. Transcriptions were done four times; representative results are shown. Primary data are shown above the graph. The maximum signal was set as 1. Averages of E_1/2_ values ± SD for promoters are specified inside the graph. **(I)** A scheme of the P*helD* DOWN (LK3004) and P*veg *+ *helD* DOWN (LK3789) promoter-*lacZ* constructs (in the latter construct, P*helD* was replaced with P*veg*). **(J)** Activities of P*helD* DOWN and P*veg + helD* DOWN promoter-*lacZ* constructs [from **(I)**] in exponential phase (OD_600 _= 0.5) in the absence of RIF. Activity of P*helD* DOWN was set as 1. The bars are averages from four independent experiments, the dots are individual experimental data, and the error bars show $ \pm $ SD. **(K)** Inducibility of constructs from **(I)** (+RIF/-RIF). Activity without RIF for each construct was set as 1. The bars are averages from four independent experiments, the dots are individual experimental data, and the error bars show $ \pm $ SD.

To explore further the properties of the two promoters and their interplay, we created a construct termed P*helD* FULL REVERSE, in which the positions of P*helD* and P*anti-helD* were swapped (Fig. 5A). The P*helD* FULL REVERSE construct was highly active and not induced by RIF in β-galactosidase assays (Fig. 5B and C), illustrating that the order of the promoters mattered.

We then compared the strengths of minimal promoter versions P*helD* CORE and P*anti-helD* CORE *in vivo* and *in vitro* (Fig. 5D-5G, for sequences see Supplementary Fig. S13). In both systems, P*anti-helD* CORE was the stronger promoter by several-fold, similarly active as the strong P*veg* CORE promoter. None of the CORE promoters was inducible by RIF on its own.

To dissect the properties of the two promoters in more detail, we evaluated three key steps in transcription initiation: (i) initial binding (closed complex formation); (ii) affinity of RNAP for the initiating NTP (iNTP; GTP for both promoters) – this reflects the ability of RNAP to form the transcription bubble, the so called open complex [49, 50]; and (iii) promoter escape.

Both promoters displayed similar affinities for RNAP (Supplementary Fig. S14A), and P*anti-helD* CORE required more iGTP for half-maximal transcription (Supplementary Fig. S14B), similar to *e.g. rrnB* P1 [13, 28], suggesting that it might be regulated by changes in the GTP concentration *in vivo*. Neither of these two results, however, offered an explanation for why P*helD* CORE was the weaker promoter and why the specific arrangement of the two promoters resulted in increased transcription from P*helD* in the presence of RIF.

Finally, promoter escape experiments revealed that RNAP was slow to escape from P*helD* CORE (promoter escape half-life, E_1/2_: 3.9 ± 0.27 min) compared to P*anti-helD* CORE (E_1/2_: 0.56 ± 0.10 min) and control promoter P*veg* CORE that behaved comparably to P*anti-helD* CORE (E_1/2_: 0.36 ± 0.05 min) (Fig. 5H). Therefore, a chimeric construct where P*helD* was replaced with P*veg* (P*veg *+ *helD* DOWN; Fig. 5I) should behave similarly to P*helD* FULL REVERSE. Indeed, this was the case: high activity even in the absence of RIF and no RIF-inducibility (Fig. 5J, 5K).

### Model of the RIF-dependent regulation of *helD* expression

**Figure 6.**
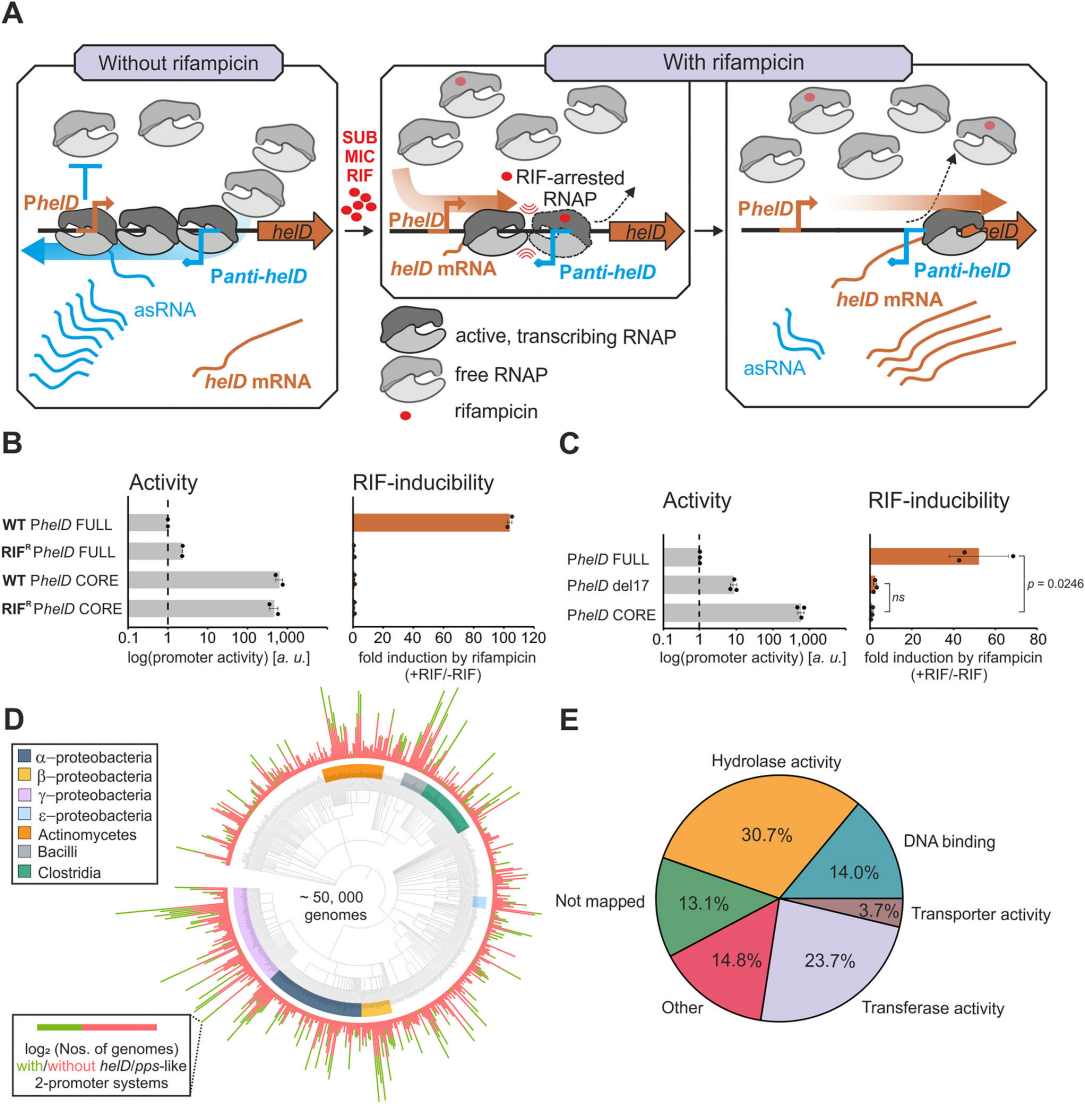
**Model of regulation of**  ***helD*****expression by RIF and bioinformatically identified dual promoter arrangements (2-promoter systems) across bacteria. (A)** Model of regulation of *helD* expression by RIF. In the absence of RIF (left), transcription predominantly occurs from the stronger P*anti-helD* promoter, which interferes with the weaker P*helD* promoter, allowing only occasional transcription from P*helD*. When RIF at sub-MIC levels is present, it primarily arrests RNAP at P*anti-helD* and stops the interference. RNAP (without RIF) from P*helD* has then sufficient time to bind and escape from the promoter. Once initiated, the elongating RNAP upon collision dislodges the arrested RNAP from P*anti-helD* (middle) and transcribes the *helD* gene (right). For more details, see the text. **(B)** Activity and inducibility (activity +RIF/-RIF) of P*helD* FULL and P*helD* CORE promoter-*lacZ* constructs in wt [P*helD* FULL (LK3005), P*helD* CORE (LK2970)] and RIF-resistant [RIF^R^, *rpoB* H482Y; P*helD* FULL (LK4439), PhelD CORE (LK4437)] genetic backgrounds in the exponential phase. Activity of P*helD* FULL wt in the absence of RIF was set as 1. The bars are averages from two independent experiments, the dots are individual experimental data, and the error bars show the range. **(C)** Activity and inducibility (activity +RIF/-RIF) of P*helD* FULL (LK3005), P*helD* CORE (LK2970), and P*helD* del17 (LK3114) promoter-*lacZ* constructs in the exponential phase. Activity of P*helD* FULL wt in the absence of RIF was set as 1. The bars are averages from three independent experiments, the dots are individual experimental data, and the error bars show ± SD. **(D)** Taxonomy tree overview of bacterial families in which two-promoter combinations, sequentially similar to those identified upstream of *B. subtilis helD* and *pps*, were identified. The NCBI taxonomy tree was trimmed to include only families that were present in the used RefSeq dataset (see Zenodo repository). The bars associated with individual leaves show the number of genome assemblies (log_2_) in a particular leaf (family) in which the two-promoter system was found (outward part of the bar, light green sector) or not (inward part of the bar, light red sector). The larger the bars, the more assemblies within that taxonomic unit. The tree leaf labels are colored if they occur in the top seven most occurring taxonomic classes by genome assembly count in our dataset. **(E)** The pie-chart shows the top four GO slim molecular function terms by the sum of per-protein weighted occurrence of the term in the proteins found downstream of candidate two-promoter system sites. Two additional fractions are also shown: (i) GO terms that were not mapped to any GO slim term used (labeled “not mapped”) and (ii) GO slim terms different from the top four terms (labeled “other”).

Based on the experimental data thus far obtained, we proposed a model of regulation of *helD* by RIF (Fig. 6A). Both promoters seem to attract RNAP equally well (Supplementary Fig. S14A). However, it takes longer for RNAP to clear P*helD* (Fig. 5H). As a result, P*anti-helD* is the dominant promoter in such conditions. In the absence of RIF, RNAPs coming from P*anti-helD* interfere with transcription from P*helD*, allowing RNAP only occasionally to escape from P*helD* (typically it is displaced by the oncoming RNAP) and transcribe the *helD* gene, explaining the relatively low levels of *helD* mRNA in the absence of the antibiotic.

In the presence of low levels of RIF (*e.g*. 1 in 100 RNAP molecules is RIF-bound), it is P*anti-helD* where a RIF-bound RNAP is more likely to be arrested due to the higher “traffic” of RNAPs passing through this promoter. This allows another RNAP molecule to interact with P*helD* (99% probability that it will be RIF-free) and, in the absence of transcription from P*anti-helD*, to escape from the promoter. This RNAP molecule then starts transcribing towards the *helD* gene, and we propose that upon collision, it dislodges the arrested RNAP from P*anti-helD*. After dissociation of the RIF-arrested RNAP, the RNAP molecule in the sense direction transcribes the *helD* gene, and the system is reset for another round. The *helD* mRNA is then translated, and HelD helps the cell combat RIF.

This model predicts that if RNAP cannot be arrested at the P*anti-helD* promoter by RIF, this regulation should not function. Such a situation would occur if RNAP contained mutation(s) preventing RIF binding. Therefore, we created a strain with the H482Y mutation in *rpoB* that is known to confer RIF resistance [18] and measured activity and RIF-inducibility of the P*helD* FULL-*lacZ* fusion in this genetic background compared to wt. Consistent with the prediction, the P*helD* FULL activity was as low as in wt, and there was no inducibility by RIF due to the continuous interference (Fig. 6B).

Another prediction of the model is that shortening the distance (39 bp including both TSS positions) between the two promoters by 17 bp would also abolish the regulation as the binding of RNAPs at either promoter would sterically interfere with RNAP binding at the other promoter (Supplementary Fig. S15). To test this, we created the respective P*helD* del17-*lacZ* fusion and compared its activity/inducibility with P*helD* FULL and P*helD* CORE (Fig. 6C). Consistently, no inducibility by RIF was observed.

### The two-promoter RIF-responsive arrangement is found upstream of other genes and in other species

To determine whether the two convergent promoter RIF-responsive regulatory setups could be found upstream of other genes, we searched the *B. subtilis* genome bioinformatically for a similar promoter arrangement (see Zenodo held_emboss_search). We then compared the results with upregulated proteins (genes) after RIF treatment (Fig. 1A, Supplementary Table S5). The overlap was two genes: *helD* (*BSU_33 450*) and *pps* (*BSU_18 830*) (Fig. 1A).

We identified two TSS in the *pps* upstream region, one in the sense direction (P*pps*) and one in the opposite direction (P*anti-pps*) by analysis of cappable-seq data from Forrest *et al.* [46]. We then experimentally examined *pps -* its effect on RIF resistance and regulation of its expression. Subsequent phenotypic analysis (Supplementary Fig. S16A, S16B), RT-qPCR using RNA purified from wt cells with or without RIF (Supplementary Fig. S16C), and β-galactosidase experiments (Supplementary Fig. S17) revealed the same type of regulation for *pps* by RIF involving two convergent promoters as already described for *helD* (Supplementary Fig. S17B-S17J).

Finally, we searched ∼50 000 bacterial genomes available in RefSeq [51] for the two convergent promoter arrangements occurring in intergenic regions close (<300 bp) to a downstream gene. The search revealed that sequences similar to the *helD* and *pps* regulatory elements were found upstream of 18 798 genes in 11 591 genome assemblies (Fig. 6D, Supplementary Table S3). We sorted the identified genes based on molecular function: we mapped known gene ontology (GO) terms to GO slim categories (Fig. 6E).

The main categories identified were hydrolases (potential cleavage of the *ansa* ring), transferase activity (*e.g*. glycosyltransferases – potential modification of RIF), transcription factors (potential regulation of RIF resistance genes), and transporter activity (*e.g*. ABC transporters – potential efflux). The search also returned both *helD* and *pps*, validating the approach (found in the Other category in Fig. 6E, see also Supplementary Table S3).

## Discussion

This study demonstrates that Class I HelD contributes to resistance of *B. subtilis* against RIF. Most notably, it uncovers a previously unrecognized RIF-responsive regulatory mechanism that relies on a pair of convergent promoters (one sense and one antisense) and their properties. This allows expression of the downstream positioned gene(s) to be induced by RIF at the transcriptional level. This promoter arrangement was experimentally validated for *helD* and *pps* in *B. subtilis*, and bioinformatic analyses revealed similar configurations upstream of these, as well as other genes, across more than a thousand bacterial species, suggesting the presence of potentially novel and widespread mechanisms of RIF resistance.

As shown in this study, *Bsu_*HelD increases the resistance of *B. subtilis* to RIF by protecting RNAP. This protective effect is primarily mediated by its coiled-coil N-terminal domain that reaches the vicinity of the RIF-binding pocket and likely induces a structural deformation that impairs RIF binding. This effect was lost in the absence of the domain or with mutations of three acidic amino acids at the tip of the domain. Interestingly, the N-terminal domain of HelD shares structural similarity with proteins such as Gre factors, which also feature a coiled-coil domain and two acidic aa at its tip. These aa participate in an essential interaction with the Mg^2+^ ion in the RNAP catalytic center [52]. This assists RNAP in the hydrolytic removal of the 3′‐proximal segment of nascent RNA in backtracked transcription elongation complexes [53]. In contrast, the corresponding acidic residues in Class I HelD are positioned further inside RNAP and do not interact directly with the catalytic Mg²⁺ ion (Supplementary Fig. S18). However, when these residues are substituted with less bulky alanines, it likely reduces the domain’s steric interference near the RIF-binding site, thereby allowing RIF to bind more effectively (Fig. 2B).

To date, structures of two classes of HelD proteins have been determined experimentally [3–5]; Class III HelD proteins were identified bioinformatically [54]. It is now evident that both Class I and II HelD proteins confer protection to respective RNAPs against RIF, though they achieve it via distinct mechanisms: Class I HelDs utilize their N-terminal domain, whereas Class II HelDs employ the PCh loop. The functional role of Class III HelD proteins in RIF resistance remains uncharacterized, and future experiments will be necessary to determine whether they share similar protective capabilities or employ novel strategies.

HelD is expressed even in the absence of RIF, albeit at a lower level, and it dissociates stalled elongation complexes [6]. However, after RIF exposure, HelD expression increases, and so does the percentage of RNAPs associated with HelD. This upregulation is dependent on a RIF concentration that is well below the MIC. This allows the cell to detect the presence of the antibiotic early and initiate a protective response.

This upregulation occurs at the transcriptional level. Although promoter-driven increases in response to RIF (including the region upstream of *helD* in *B. subtilis*) were observed previously in various bacterial species, the underlying regulatory mechanisms have remained unclear. Previous studies often attributed this to “differential promoter sensitivity” without offering a mechanistic explanation [55–58]. Here, we provide a mechanistic model in which the regulation of *helD* and *pps* expression is governed by transcriptional interference between two convergent promoters located upstream of the gene. The same regulation of the *B. subtilis helD* and *pps* genes is also reflected in their similar response to other stimuli ([47]; Supplementary Fig. S19).

Convergent promoters and their regulation by transcriptional interference are well-recognized phenomena in gene expression control, found from phages to eukaryotes. This interference is typically asymmetric, where a strong promoter suppresses activity from a weak, opposing promoter [59]. Examples of this effect are the convergent promoters of the lysis-lysogeny switch in the bacteriophage 186 [59] or similar regulatory architecture that contributes to the control of the virulence gene *icsA* of *Shigella flexneri* [60]. In eukaryotes, convergent promoters are estimated to comprise about a quarter of all active transcript start sites with consequences for gene expression regulation [61]. This regulatory feature is also of interest in synthetic biology as a tool to achieve tunable gene expression [62]. Involvement of convergent promoters in bacterial antibiotic resistance, however, has not been recognized until now.

The current study reveals how the two convergent promoter setups detect RIF with high sensitivity that relies on the interaction between the two promoters rather than on differential sensitivity to RIF. As shown in detail for *helD* expression, the sense (P*helD*) promoter is weaker with slower promoter escape. We note that both P*anti-helD* and P*anti-pps* contain unusually long 19 bp long spacers (consensus 17 bp; [63]); it is tempting to speculate that this feature, as well as the lower stability of the open complex (indirectly determined by iNTP requirements for half-maximal transcription; Supplementary Fig. S14B) may be important for the dissociation of the RIF-arrested RNAP by the incoming RNAP from P*helD*. We note that the upregulation of P*helD* by RIF is more pronounced *in vivo* than *in vitro*, but the key regulatory characteristics are already evident in the minimal *in vitro* system. Nevertheless, it is possible that additional factor(s) may amplify the effect in the cell. Finally, the sensitivity of P*anti-helD* to a wider range of GTP concentrations is consistent with increased expression of *helD* during sporulation [47], where the GTP concentration (a key regulatory metabolite in *B. subtilis*, [64]) is lower relative to the exponential phase [65]. This may decrease the activity of P*anti-helD* and allow increased transcription from P*helD*. The biological reasons underlying the increased demand for HelD during sporulation remain to be elucidated, though.

This RIF-sensing dual-promoter regulatory system was found upstream of genes encoding HelD and Pps in numerous species. Furthermore, our bioinformatic search of available bacterial genomes found two convergent promoters upstream of 18 798 genes, with many genes found in multiple genomes. Such genes encode proteins that may mediate RIF resistance in respective species. Examples of these proteins with a potential to play a role in RIF resistance include various membrane-associated exporters/efflux pumps (*e.g*. encoded by BURCE16_RS07800) from *Burkholderia* and HGI30_RS05345 from *Paenibacillus* or potential RIF modifiers such as acyltransferase in *Serratia marcescens* (FG172_RS10935) and *Xylophilus* sp. (R9 × 41_RS08475), acetyltransferase in *Lawsonibacter asaccharolyticus* (L9O85_RS00885), methyltransferase in *Achromobacter xylosoxidans* (AL509_RS24335), and glycosyltransferase in *Acetobacter pasteurianus* (S1001342_RS06745).

We also clustered the identified proteins (encoded downstream of the two-promoter regulatory setup) based on aa sequence similarity (Supplementary Table S4). Based on this criterion, Pps, TetR/AcrR family transcriptional regulator, and glycosyltransferase family A protein were among the most commonly identified proteins. Additionally, proteins such as SpeB (*e.g*. encoded by AB8K35_RS08165) that are involved in spermidine biosynthesis were near the top. Interestingly, spermidine was shown to increase efflux in *Acinetobacter baumanii* [66], and it is tempting to speculate that it may contribute to RIF resistance. Finally, the search also yielded proteins of unknown function that may represent principally novel mechanisms of RIF resistance.

The two convergent promoters thus appear to represent a widespread regulatory mechanism that allows bacteria to survive the presence of RIF, at least through HelD and Pps. The other proteins identified by the bioinformatic search need to be tested experimentally. It would also be of interest to investigate whether this mechanism can sense other bacterial RNAP-targeting compounds, such as fidaxomicin, which is used clinically to treat *Clostridium difficile* infections [67].

Not all *helD* genes, however, are regulated by two convergent promoters. In *Actinobacteria*, genes encoding proteins involved in protection of the cell against RIF (including HelD) feature the so-called “rifampicin-associated element” (RAE) in their 5′ UTR [68]. This 19 bp long palindrome is possibly bound by an unknown transcription factor responsive to RIF. Interestingly, this RAE overlaps with the more downstream of the two sense-oriented promoters located in the 5′ UTR [45]. Despite these observations, the precise regulatory mechanism involving the RAE and its potential transcriptional regulator remains to be elucidated.

### Concluding remarks

Challenging bacteria with sub-MIC amounts of antibiotics is a powerful approach to detect evolutionarily conserved, but previously unrecognized, mechanisms of antibiotic resistance [1, 8]. In addition to HelD and Pps, our initial search presented here identified other proteins in *B. subtilis* (Supplementary Table S5) that represent the RIF resistome. These proteins may directly or indirectly protect the cell against RIF. Research addressing this issue is in progress.

Finally, we envisage that data mining available studies that used this approach and combining this information with newly designed experiments involving a broader range of species/antibiotics is likely to uncover other naturally evolved antibiotic resistance strategies. This will enhance our understanding of the diversity and complexity of antibiotic resistance mechanisms and offer valuable insights into potential diagnostic and therapeutic applications to help combat the escalating threat of antibiotic-resistant infections.

## Supplementary Material

gkaf1407_Supplemental_Files

## Data Availability

The mass spectrometry proteomics data have been deposited to the ProteomeXchange Consortium via the PRIDE [21] partner repository with the dataset identifier PXD066046 and 10.6019/PXD066046. Data and code for the analysis are deposited at Zenodo archive [https://doi.org/10.5281/zenodo.15784301].
