# Supplementary material for: Bacteria sense the antibiotic rifampicin through a widespread dual-promoter based alarm system
Source: Nucleic Acids Res. 2026 Jan 14;54(2):gkaf1407. doi: 10.1093/nar/gkaf1407 (PMC12802891; doi:10.1093/nar/gkaf1407)
Supplement: gkaf1407_Supplemental_Files [file gkaf1407_supplemental_files.zip › Sudzinova-Balgova_Supplementary information_NAR.pdf]

Supplementary information

Bacteria sense the antibiotic rifampicin through a widespread dual-promoter based alarm system

Petra Sudzinová[1,†], Tamara Knežová Balgová[1,2,†], Marek Schwarz[1,3], Klára Juříková Mikesková[1], Karolína Hegrová[1], Dragana Vítovská[1], Priyanka Rawat[1], Šárka Bobková[1], Veronika Kočárková[1,2], Saran Natarajan[1] Debora Pospíšilová[1], Alena Křenková[4], Martin Hubálek[4], Petr Halada[5], Ivan Barvík[6], Tomáš Kovaľ[7], Jan Dohnálek[7], Jana Wiedermannová[1], Hana Šanderová[1], Libor Krásný[1,*]

[1]*Laboratory of Microbial Genetics and Gene Expression, Institute of Microbiology of the Czech Academy of Sciences, Vídeňská 1083, 142 20 Prague, Czech Republic*

[2]*Department of Genetics and Microbiology, Faculty of Science, Charles University, Viničná 5, 128 44 Prague, Czech Republic*

[3]*Laboratory of Bioinformatics, Institute of Microbiology of the Czech Academy of Sciences, Vídeňská 1083, 142 20 Prague, Czech Republic*

[4]*Department of Mass Spectrometry of Biopolymers, Institute of Organic Chemistry and Biochemistry of the Czech Academy of Sciences, Flemingovo náměstí 542/2, 160 00 Prague, Czech Republic*

[5]*Laboratory of Structural Biology and Cell Signaling, Institute of Microbiology of the Czech Academy of Sciences, Vídeňská 1083, 142 20 Prague, Czech Republic*

[6]*Institute of Physics, Faculty of Mathematics and Physics, Charles University, Ke Karlovu 5, 121 16 Prague 2, Czech Republic*

[7]*Laboratory of Structure and Function of Biomolecules, Institute of Biotechnology of the Czech Academy of Sciences, Průmyslová 595, 252 50 Vestec, Czech Republic*

*To whom correspondence should be addressed. Email: krasny@biomed.cas.cz, Phone: +420 296443208

†The first two authors contributed equally to this work

This file contains:

Supplementary Figures S1 – S19

Supplementary Tables S1 – S5

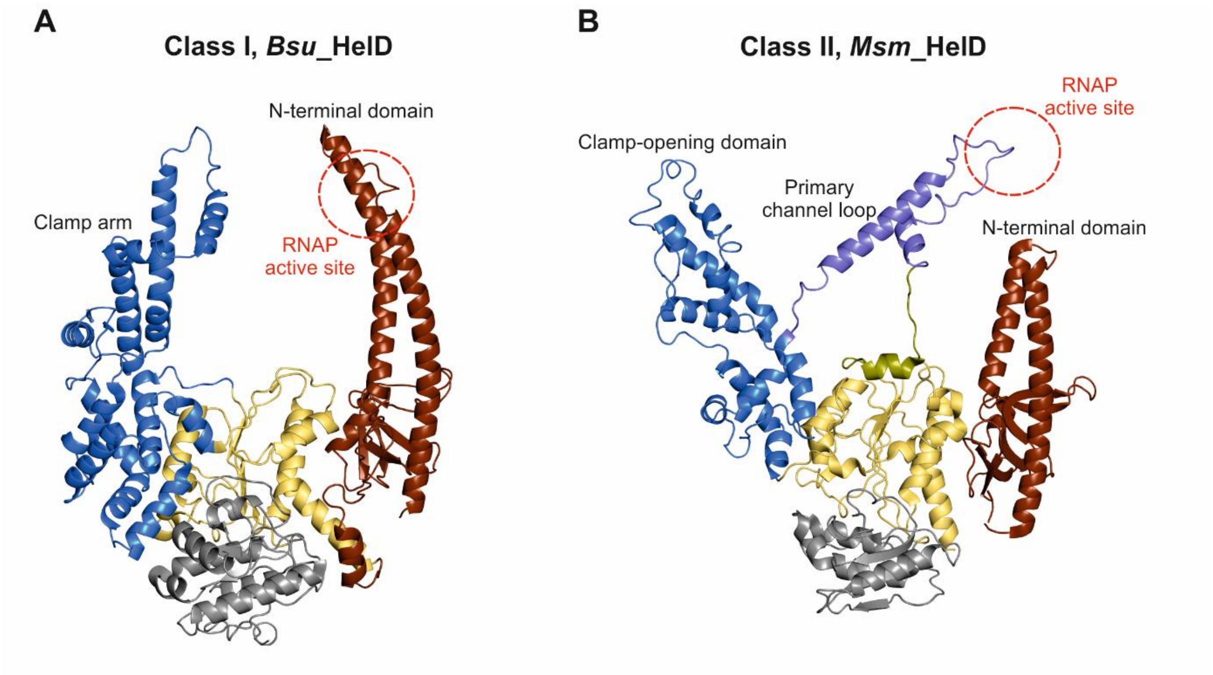

Supplementary Fig. S1: Comparison of Class I and Class II HelD

(A) Secondary structure representation of HelD Class I from *Bacillus subtilis,* PDB ID: 6ZFB, color coded according to **Figure 2A**, N-terminal domain – chocolate brown, ATPase domains 1A-1 and 1A-2 – yellow, Clamp arm – blue, ATPase C-terminal domain – grey.
(B) The same representation of the structure of HelD Class II from *Mycobacterium smegmatis*, PDB ID: 6YYS, color coded similarly as in panel **(A)**: N-terminal domain – chocolate brown, ATPase domains 1A-1 and 1A-2 – yellow, Clamp-opening domain – blue, Primary channel loop – violet, 1A-extension – olive, ATPase C-terminal domain – grey. Orientation of HelD in panel **(B)** corresponds to that in panel **(A)**, based on superposition of RNA polymerase complexes with focus on the β subunit and polymerase active site. Notice the differences in arrangement, size, and build-up of the N-terminal and Clamp arm/Clamp-opening domain, which enable the Class I to reach the RNAP active site (marked by circle) via its N-terminal domain, while Class II achieves this via the Primary channel loop.

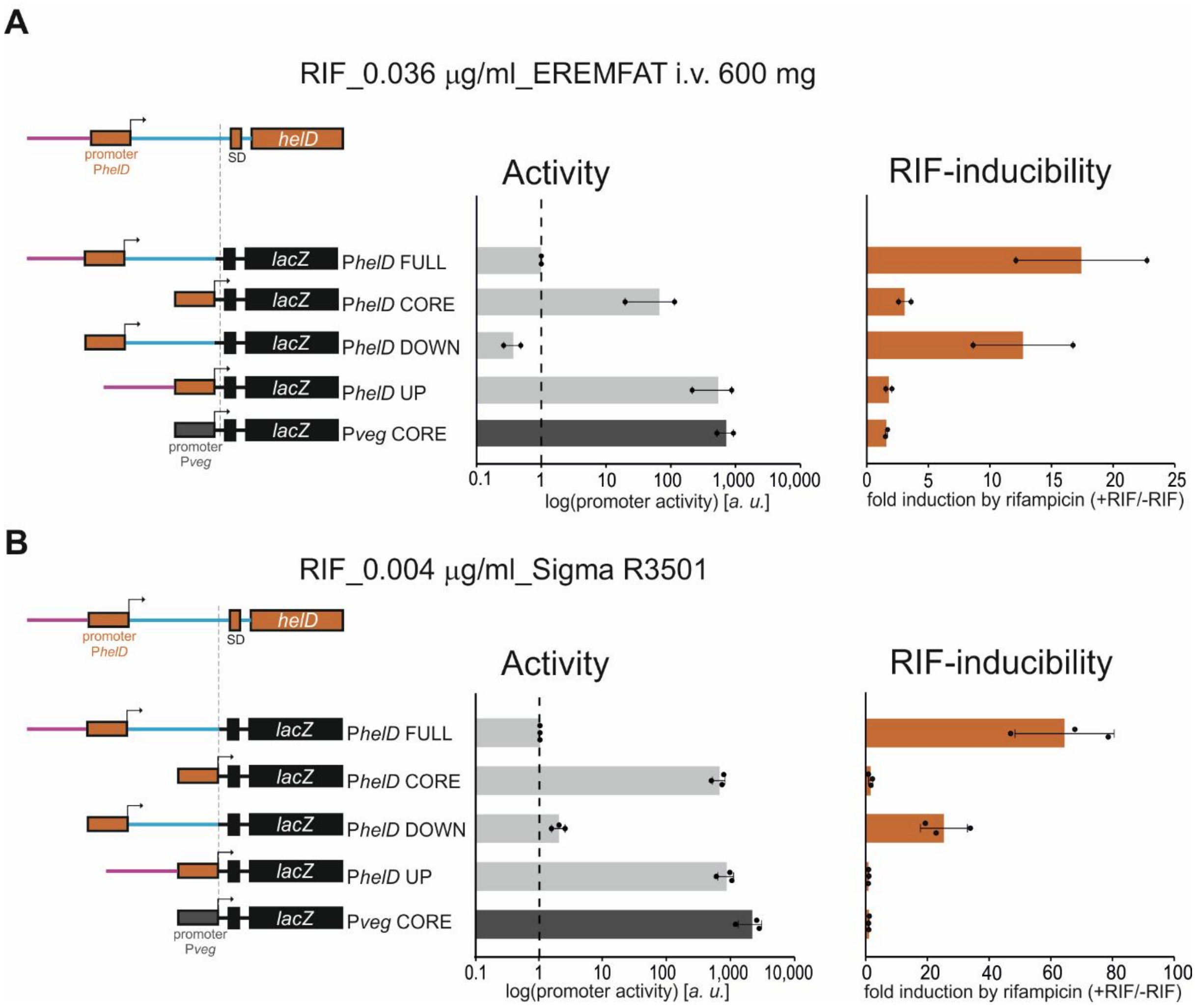

Supplementary Figure S2. Comparison of rifampicin from two different manufactures and its effect on activity of P*helD* FULL, CORE, DOWN, UP, and P*veg* CORE

(A) β-galactosidase assay with a rifampicin EREMFAT (i.v. 600 mg) in a concentration of 0.036 µg/ml. A scheme of P*helD* FULL (LK3005), CORE (LK2970), DOWN (LK3004), UP (LK3038), P*veg* CORE (LK3040) promoter-*lacZ* constructs. **Left panel** – a scheme of the promoters. **Middle panel** – activities of promoter-*lacZ* constructs in exponential phase (OD_{600} = 0.5) in the absence of RIF. Activity of P*helD* FULL was set as 1. The data shows averages from two independent experiments, the dots are individual experimental data, the error bars show the range. **Right panel** – inducibility of constructs (+RIF/-RIF). Activity without rifampicin for each construct were set as 1. The bars are averages from two independent experiments, the dots are individual experimental data, the error bars show the range.
(B) β-galactosidase assay with a rifampicin Sigma R3501 in a concentration of 0.004 µg/ml. **Left panel** – a scheme of P*helD* FULL, CORE, DOWN, UP, P*veg* CORE promoter-*lacZ* constructs. **Middle panel** – activities of promoter-*lacZ* constructs in exponential phase (OD_{600} = 0.5) in the absence of RIF. Activity of P*helD* FULL was set as 1. The data shows averages from three independent experiments, the dots are individual experimental data, the error bars show ± SD. **Right panel** – inducibility of constructs (+RIF/-RIF). Activity without rifampicin for each construct were set as 1. The data shows averages from three independent experiments, the dots are individual experimental data, the error bars show ±SD.

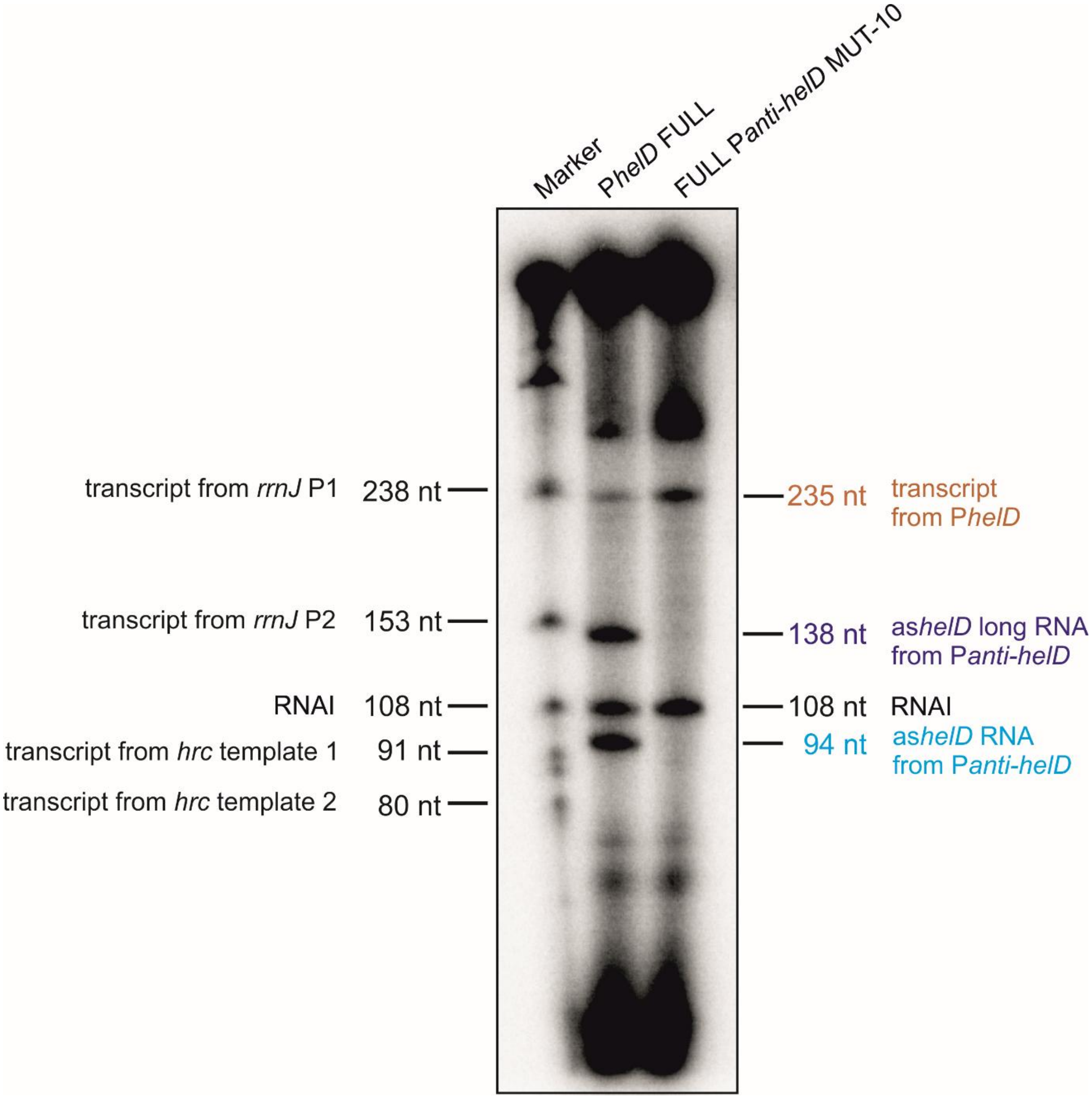

Supplementary Figure S3. Transcripts from the P*helD* FULL and FULL P*anti-helD* MUT-10 constructs alongside with a molecular size marker

In vitro multiple round transcriptions from the P*helD* FULL (LK2994) and FULL P*anti-helD* MUT -10 (LK3229) constructs were performed in the absence of RIF. **Molecular size marker:** transcription (σ^A-dependent) from LK1834 plasmid (bearing a *B. subtilis rrnJ* promoter fragment) yields transcripts of 238 nt (from *rrnJ* P1), 153 nt (from *rrnJ* P2) and 108 nt (from P-RNA1, an integral part of the plasmid backbone required for its replication control). Furthermore, two fragments of the promoter (σ^A-dependent) region of different lengths of the *B. subtilis hrc* gene yields transcripts of 80 nt (from *hrc* template 1) and 91 nt (from *hrc* template 2).

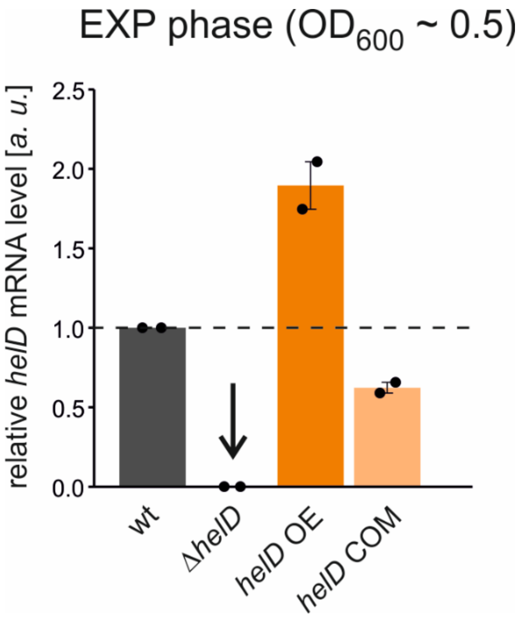

Supplementary Figure S4. Levels of *helD* mRNA in the selected strains

Relative levels of *helD* mRNAs determined with RT-qPCR in wt (LK2711), Δ*helD* (LK2840), *helD* overexpression (LK2934) and *helD* complementation (LK2935) strains in LB medium in mid-exponential phase. The relative *helD* mRNA level in the wt strain was set as 1. The graph (y-axis log scale) shows averages from two independent experiments, the dots are individual experimental data, the error bars show the range.

A

MIC values of rifampicin for selected strains

**Name**	**Genotype**	**MIC [µg/ml]**
LK2711	wt	0.063
LK2840	Δ*helD*	0.031
LK2934	*helD* OE	0.125
LK2935	*helD* COM	0.063
LK3449	Δ*pps*	0.016
LK3451	Δ*helD*Δ*pps*	0.008
LK3772	HelDΔN	0.031
LK3784	HelDtipMUT	0.031

B

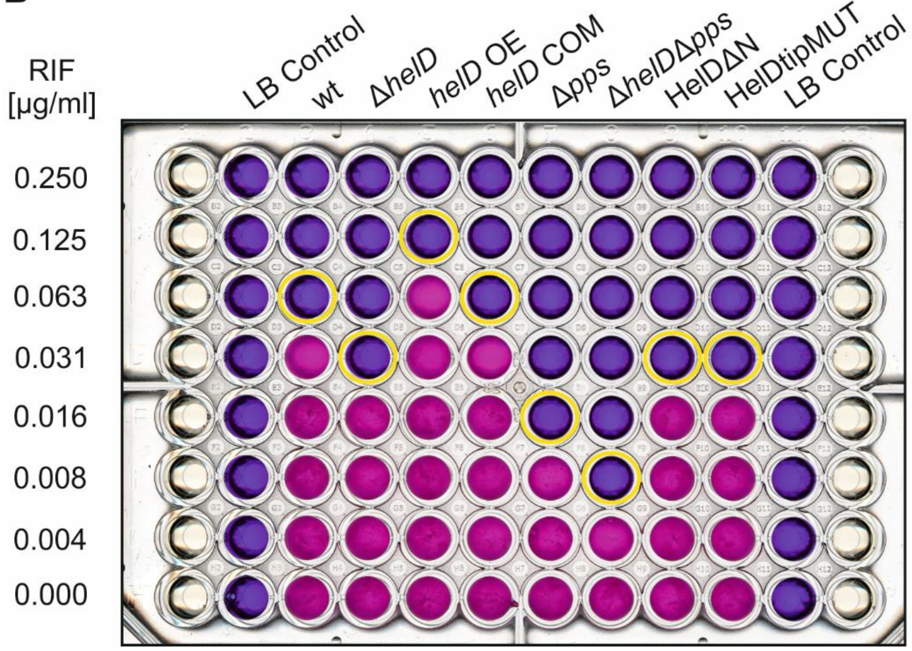

Supplementary Figure S5: Determination of minimum inhibitory concentrations (MIC) of rifampicin for selected *B. subtilis* strains

(A) Table of MIC values of selected strains determined by broth microdilution method. The values represent the lowest concentration of RIF in which there was no visible growth (well remained blue).

(B) A representative result from **(A)**. To determine cell viability, resazurin was added to the cells. Pink wells indicate viable cells, and blue wells indicate absence of viable cells. The yellow circles indicate the MIC values for individual strains. The experiment was repeated four times with identical results.

A B

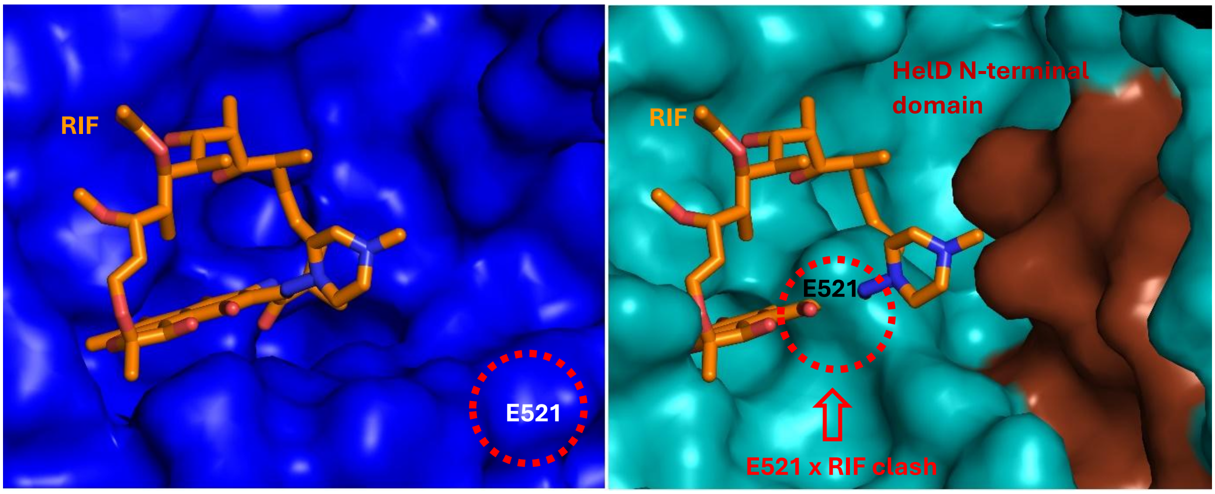

Supplementary Figure S6: Deformation of the expected rifampicin binding pocket in the presence of HelD

(A) Surface representation of the expected rifampicin binding pocket in the RNAP elongation complex from *B. subtilis* [PDB ID: 6WVJ, (Newing *et al.*, 2020)]. The pocket is undeformed, and binding of rifampicin is possible. β-E521 is indicated with the red dashed circle.
(B) Surface representation of the same expected rifampicin binding pocket in the RNAP-HelD complex [PDB ID: 6ZFB, (Pei *et al.*, 2020)]. The presence of the HelD N-terminal domain (shown in brown surface representation and marked) alters the position of β-E521 (red dashed circle and arrow), creating a clash with the putative rifampicin position. The putative position of rifampicin (RIF, shown as sticks with carbon in orange) in both panels is adopted from the *M. tuberculosis* RNAP complex [PDB ID: 5UHC, (Lin *et al.*, 2017)]. The graphics were created using PyMOL.

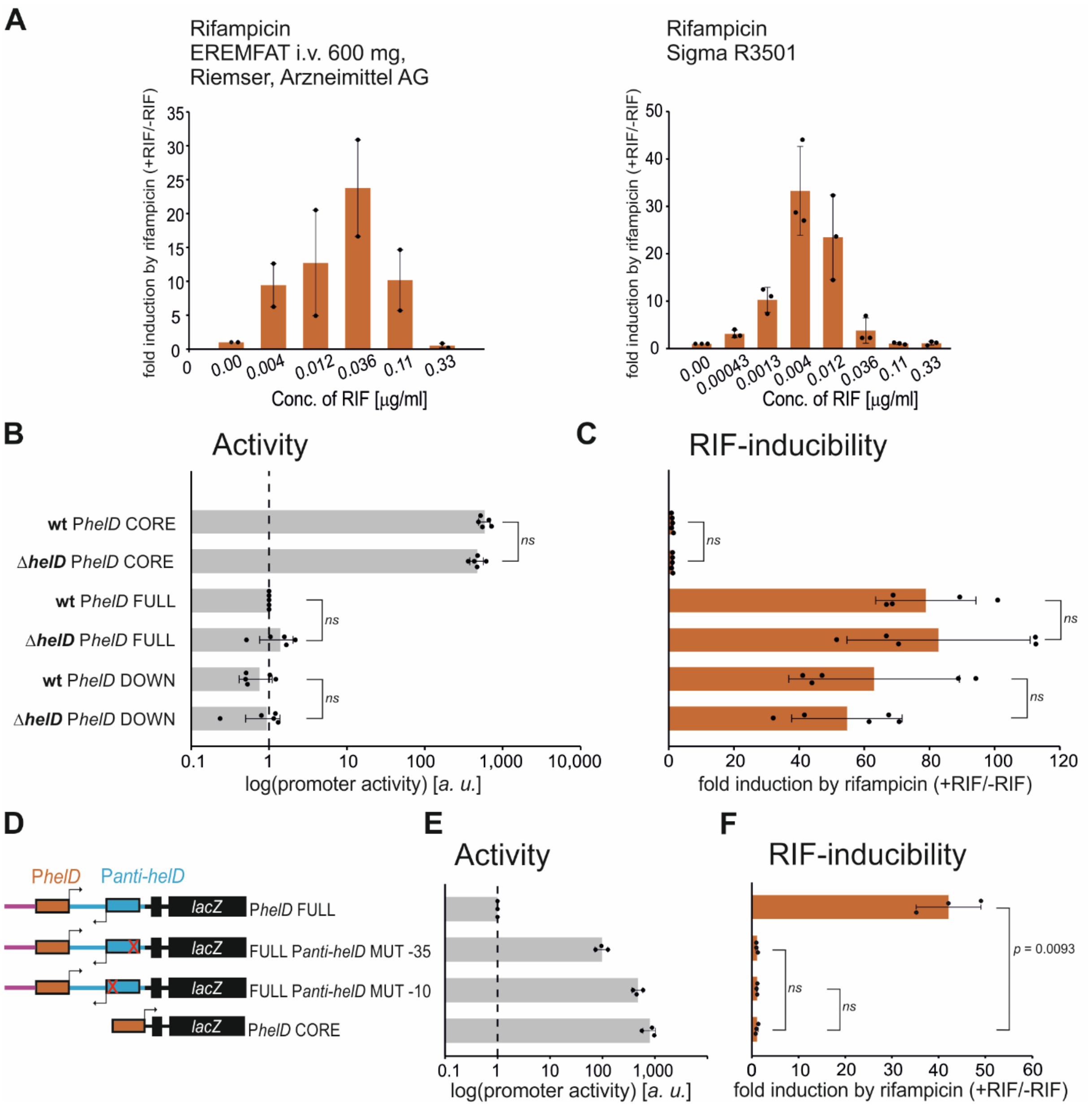

Supplementary Figure S7. Regulation of expression of *helD* by rifampicin

(A) Promoter activity of P*helD* FULL (LK3005) in the absence and presence of various concentrations rifampicin from two manufacturers (EREMFAT i.v. 600; Sigma R3501). **Left panel** – the graph shows inducibility of the P*helD* FULL-*lacZ* fusion by rifampicin EREMFAT i.v. 600, expressed as activity in the presence of rifampicin to activity in its absence (+RIF/-RIF). The activity without RIF was set as 1. The bars are averages from two independent experiments, the dots are individual experimental data, the error bars show the range. The highest induction of the P*helD* FULL construct in that case was at 0.036 µg/ml rifampicin. **Right panel** – the graph shows inducibility of P*helD* FULL-*lacZ* fusion by rifampicin Sigma R3501, expressed as activity in the presence of rifampicin to activity in its absence (+RIF/-RIF). The activity without RIF was set as 1. The bars are averages from three independent experiments, the dots are individual experimental data, the error bars show ± SD. The highest induction of the P*helD* FULL construct in that case was at 0.004 µg/ml rifampicin.

(B) Activities of P*helD* CORE, P*helD* FULL and P*helD* DOWN promoter-*lacZ* constructs in a *wt* [P*helD* CORE (LK2970), P*helD* FULL (LK3005), P*helD* DOWN (LK3004)] and Δ*helD* background [(P*helD* CORE (LK2973), P*helD* FULL (LK3007) and P*helD* DOWN (LK3006)]; [exponential phase (OD_{600} = 0.5)] in the absence of RIF. Activity of P*helD* FULL was set as 1. The data shows averages from five independent experiments, the dots are individual experimental data, the error bars show ± SD. *p*-values were calculated using a two-tailed, unpaired *t*-test; "*ns*" indicates non-significance, $p > 0.05$.

(C) Inducibility of P*helD* CORE, P*helD* FULL and P*helD* DOWN promoter-*lacZ* constructs in a *wt* and Δ*helD* background (+RIF/-RIF). Activity without rifampicin for each construct were set as 1. The bars are averages from five independent experiments, the dots are individual experimental data, the error bars show ± SD. *p*-values were calculated using a two-tailed, unpaired *t*-test; "*ns*" indicates non-significance, $p > 0.05$.

(D) A scheme of P*helD* FULL (LK3005), FULL P*anti-helD* MUT -35 (LK3233), FULL P*anti-helD* MUT -10 (LK3234) and P*helD* CORE (LK2970) promoter-*lacZ* constructs.

(E) Activities of promoter-*lacZ* constructs [from **(D)**] in exponential phase (OD_{600} = 0.5) in the absence of RIF. Activity of P*helD* FULL was set as 1. The data shows averages from three independent experiments, the dots are individual experimental data, the error bars show ± SD.

(F) Inducibility of constructs from **(D)** (+RIF/-RIF). Activity without rifampicin for each construct were set as 1. The bars are averages from three independent experiments, the dots are individual experimental data, the error bars show ± SD. *p*-values were calculated using a two-tailed, unpaired *t*-test and indicated in the graph; "*ns*" indicates non-significance, $p > 0.05$.

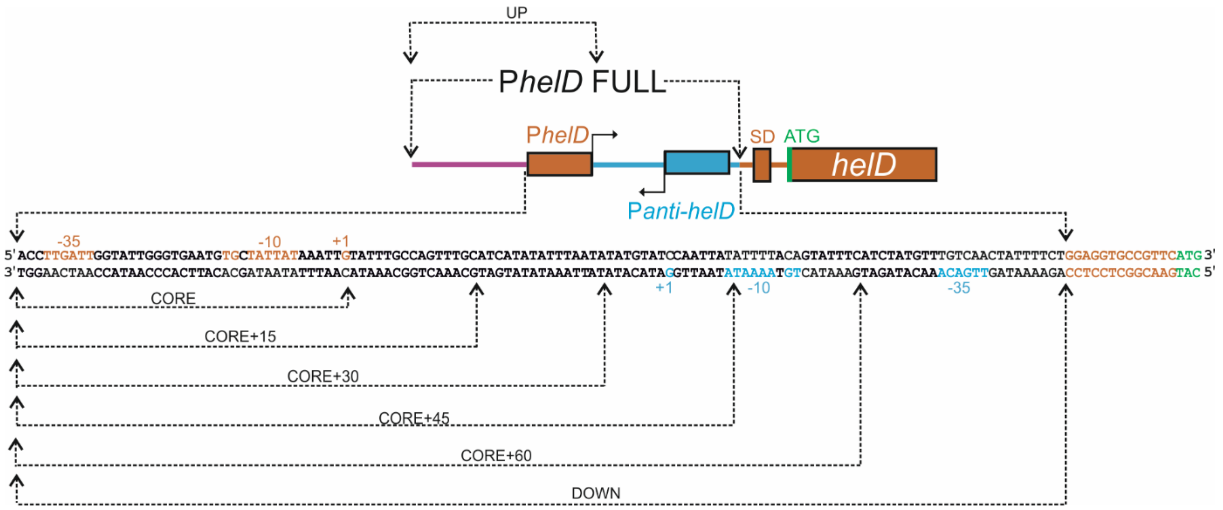

Supplementary Figure S8. A scheme and nucleotide sequence of the *helD* gene upstream region, showing the constructs employed in this study

The scheme contains P*helD* and P*anti-helD* promoters, Shine-Dalgarno (SD, ribosomal binding site) and start codon (ATG, green). P*helD* is highlighted with dark orange, P*anti-helD* with blue. Constructs P*helD* FULL (LK3005), UP (LK3038), CORE (LK2970) and DOWN (LK3004) were used for initial β-galactosidase experiments (see **Figures 3C-3E** for activity/inducibility). To identify sequence(s) responsible for the RIF-dependent regulation we created the following deletion series: CORE+15 (LK3117), CORE+30 (LK3118), CORE+45 (LK3119) and CORE+60 (LK3120) (see **Figures 3F-3H** for activity/inducibility).

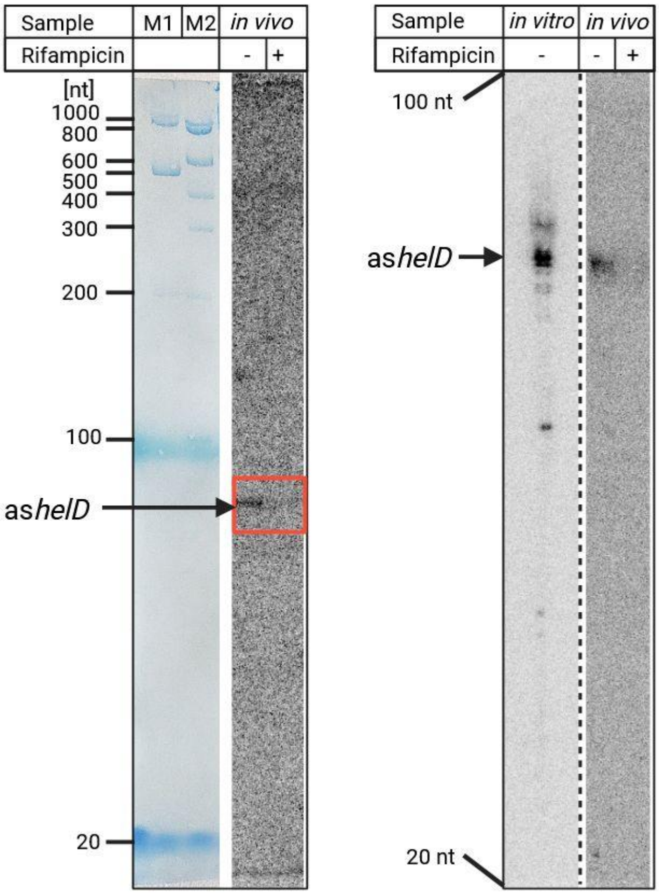

Supplementary Figure S9. Northern blot detection of as*helD* RNA

Left panel: Exponentially growing cells were/were not (+/-) treated with a subinhibitory concentration of rifampicin (0.03 µg/ml) and RNA was isolated. 5 µg of total RNA was separated on 7% PAA gel (50 min) and as*helD* RNA was detected by radiolabeled Northern blotting. For the size determination (nt, nucleotides), the membrane is superimposed next to the methylene-stained picture of the same membrane. M1- RiboRuler High Range RNA Ladder (Thermo Scientific), M2- RiboRuler Low Range RNA Ladder (Thermo Scientific). Approximate migration (nt) of Bromophenol blue and Xylene cyanol dyes on 7% PAA are indicated. The red rectangle specifies the area shown in **Figure 4E** in the main text. **Right panel:** The same samples were run for 90 min and the part of gel between Bromophenol blue and Xylene cyanol dyes was used for Northern blotting. *In vitro* transcribed RNA [produced by *B. subtilis* RNAP from P*helD* FULL (LK2994)] was used as a size marker. The experiment was performed in three biological replicates; representative gels are shown.

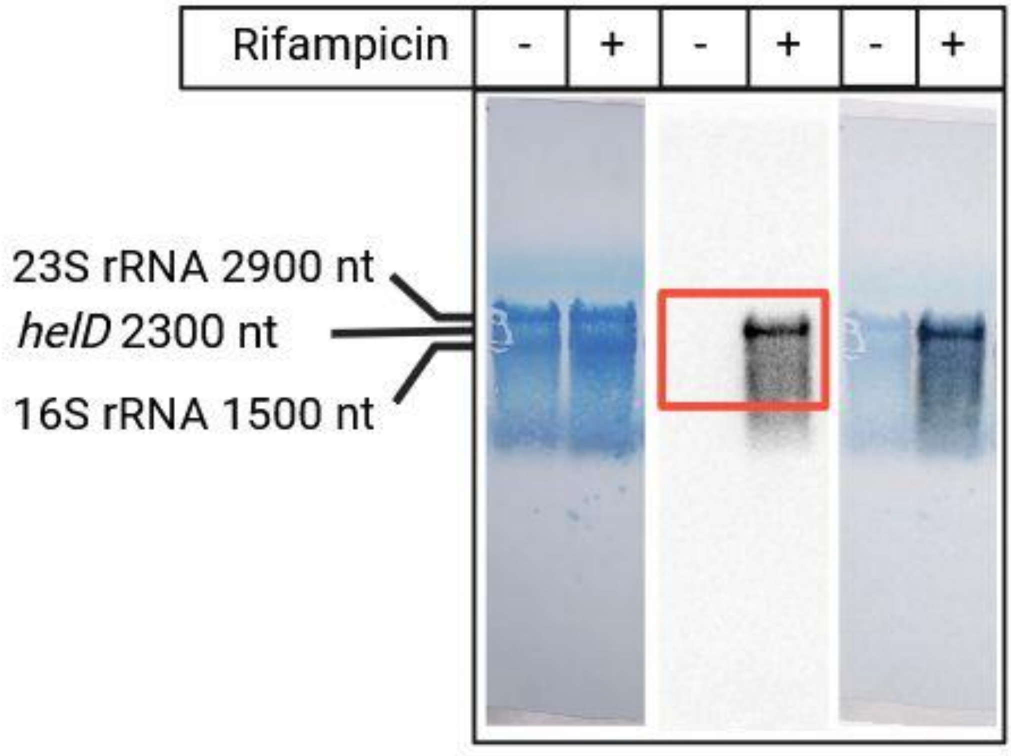

Supplementary Figure S10. Northern blot detection of *helD* mRNA

RNA (the same samples as used for detection of as*helD* in **Supplementary Figure S9**) was run on 1.5% agarose gel and *helD* mRNA was detected by Northern blotting. **Left panel:** Methylene Blue stained membrane. rRNAs with respective sizes (nt) are indicated. **Middle panel:** Northern blot detection of *helD* mRNA by radiolabeled probe. **Right panel:** Overlay of the left and middle panels. The red rectangle specifies the area shown in **Figure 4F** in the main text. The experiment was performed in three biological replicates; representative gels are shown.

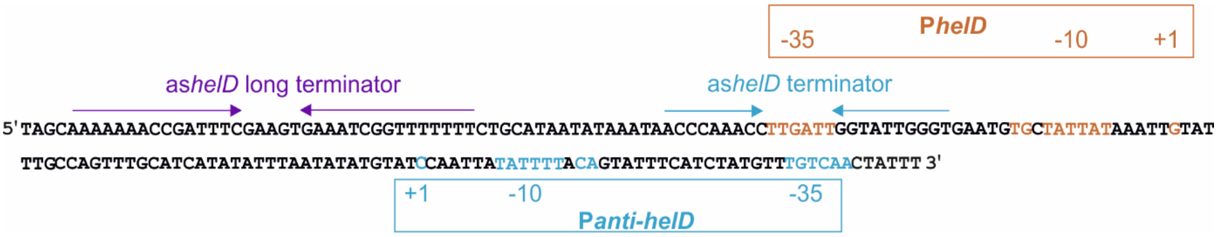

Supplementary Figure S11. Position of two putative intrinsic terminators (for transcription from P*anti-helD*) within the P*helD* FULL construct

The scheme shows the nontemplate strand (5'→3'). P*helD* is highlighted with dark orange, P*anti-helD* with blue. Terminator palindromes are indicated with arrows. The P*anti-helD* proximal terminator, the as*helD* terminator, is highlighted with blue. Transcription from P*anti-helD* only partially terminates here *in vitro* but this is the dominant terminator *in vivo*. The P*anti-helD* distal terminator, the as*helD* long terminator, is highlighted with purple. Transcription *in vitro* from P*anti-helD* that reads through the more proximal promoter terminates here.

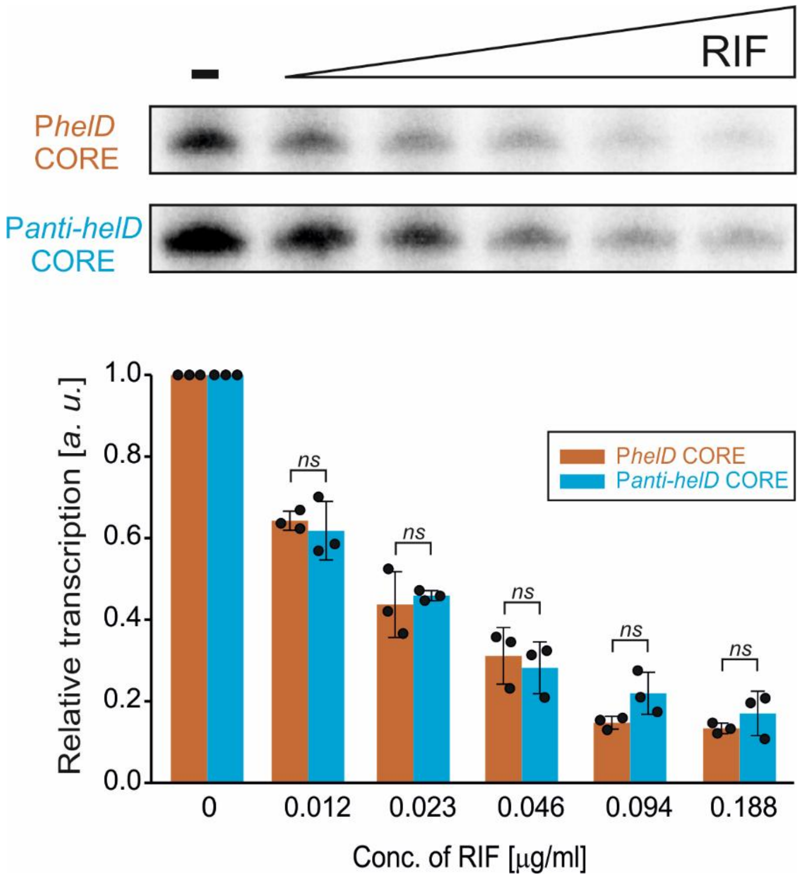

Supplementary Figure S12. Sensitivity of P*helD* and P*anti-helD* to rifampicin

In vitro multiple round transcriptions from the P*helD* CORE (LK2977) and P*anti-helD* CORE (LK3610) were performed with increasing amounts of RIF (indicated below the graph). Representative primary data are shown above the graph. Transcription in the absence of RIF was normalized to 1 to facilitate visualization of promoter sensitivity to RIF. The bars show averages of three independent experiments, the dots are individual experimental data, the error bars show ± SD.

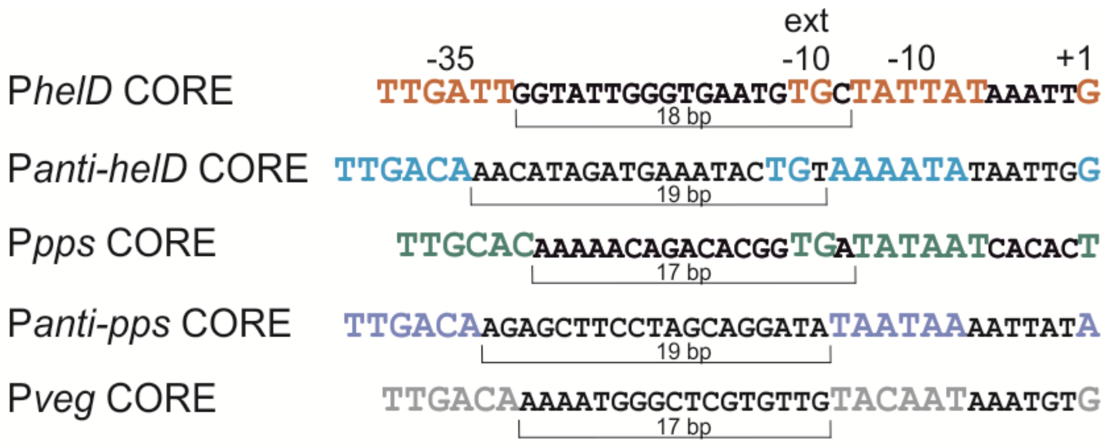

Supplementary Figure S13. Nucleotide sequences of the CORE promoter variants

Sequences (nontemplate strands, 5'→3') of P*helD* CORE (LK2970), P*anti-helD* CORE (LK3610), P*veg* CORE (LK3040) P*pps* CORE (LK3481) and P*anti-pps* CORE (LK3614) are shown. In the sequences, the -35 region (-35), -10 region (-10), extended -10 promoter element (ext -10), and transcription start site (+1) are indicated. The spacer length between the -35 and -10 regions is also indicated.

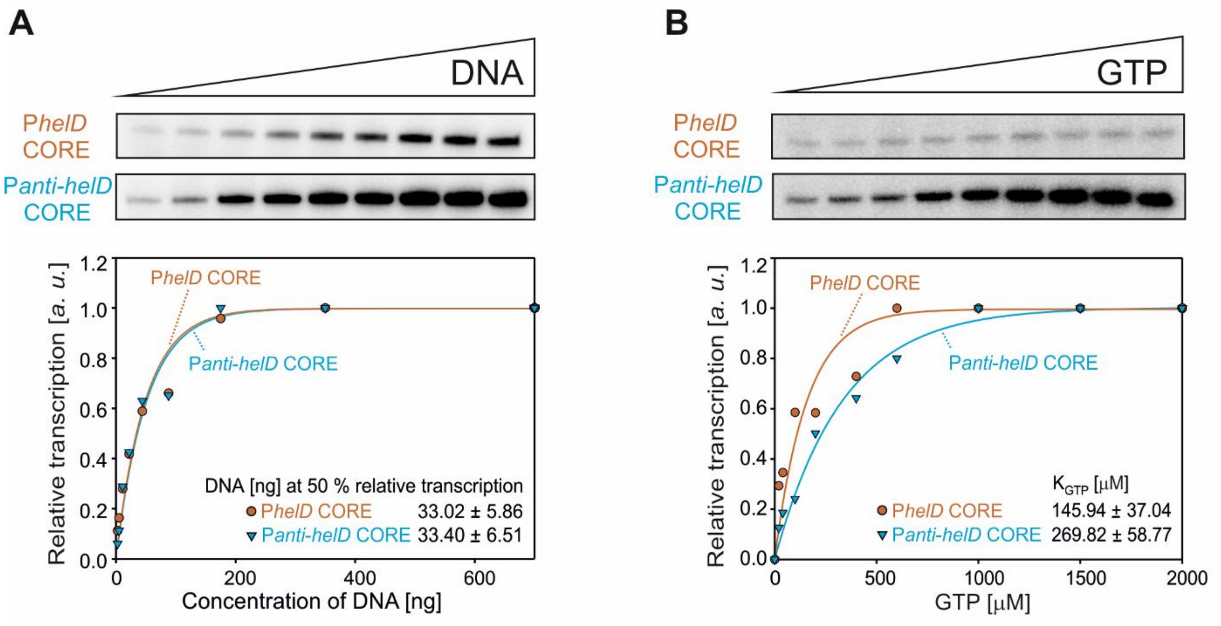

Supplementary Figure S14. Properties of P*helD* and P*anti-helD* in transcription initiation

(A) Affinity of RNAP for promoter DNA (closed complex formation). Multiple round transcriptions with increasing amounts of the supercoiled DNA template were used to determine the initial binding of RNAP to the P*helD* CORE (LK2977) and P*anti-helD* CORE (LK3610) promoters. Transcriptions were done four times; representative results are shown. Primary data are shown above the graph. Maximum signal for each construct was set as 1. Averages of DNA (promoter) concentrations to achieve 50% relative transcription ± SD are specified inside the graph.

(B) Affinity of RNAP for the initiating NTP (open complex formation). Multiple round transcription assays were performed to determine the iGTP (iNTP) concentration needed for half-maximal transcription from the P*helD* CORE (LK2977) and P*anti-helD* CORE (LK3610) promoters. Transcriptions were done three times; representative results are shown. Primary data are shown above the graph. Maximum signal for each construct was set as 1. Averages of K_{GTP} values ±SD for promoters are specified inside the graph.

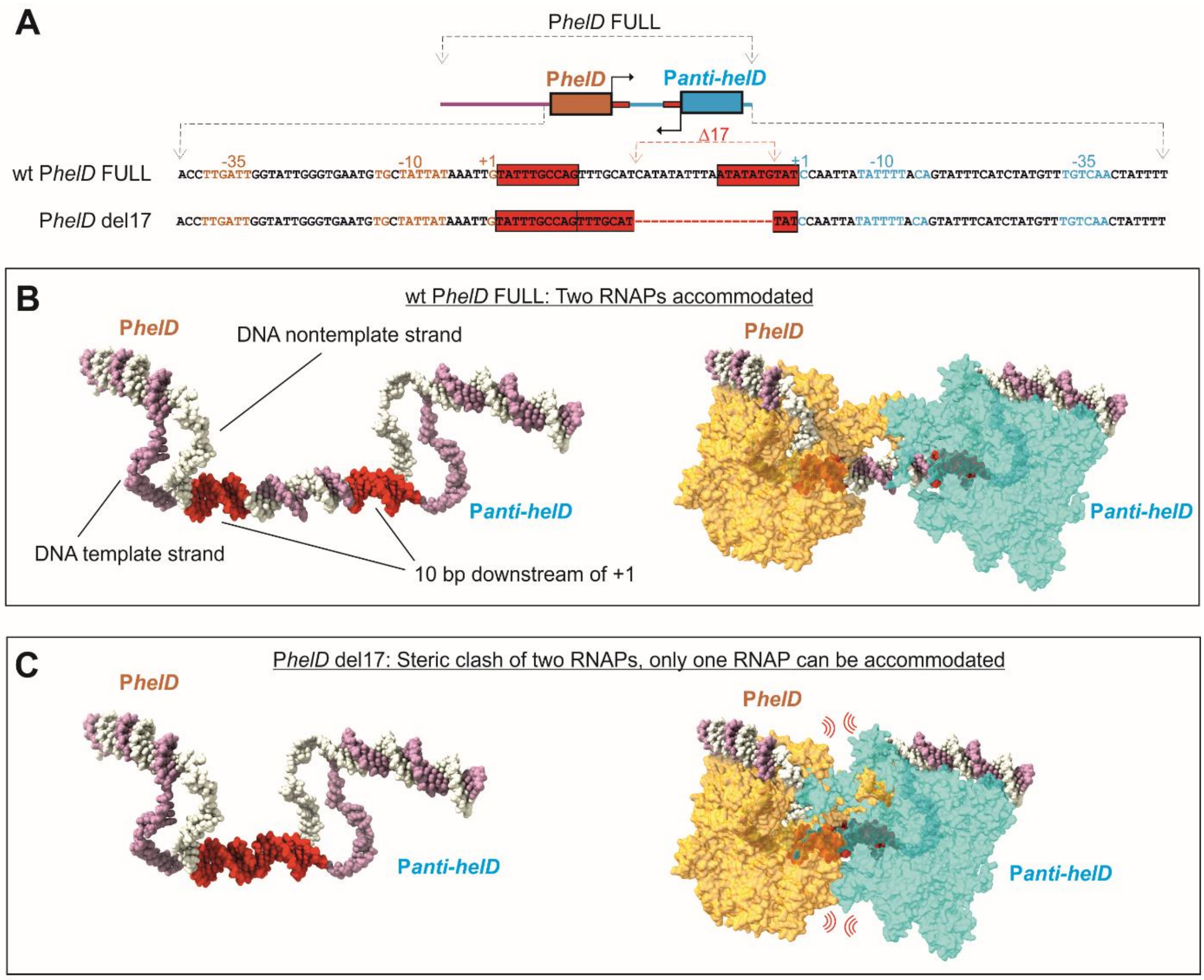

Supplementary Figure S15. RNAP cannot be simultaneously accommodated on P*helD* and P*anti-helD* when the distance between the two promoters is shortened

(A) A scheme and a sequence of the relevant part of the wt P*helD* FULL construct (top sequence). The bottom sequence (P*helD* del17) is shortened by 17 bp between P*helD* and P*anti-helD* as indicated with the red dashed line. The red rectangles are used as rulers to facilitate visualization and solely indicate 10 bp downstream of each promoter.
(B) A model of a DNA fragment containing wt P*helD* and P*anti-helD* (left) and the same DNA fragment with RNAPs accommodated at the two promoters (right). With the distance of 37 bp between the two promoters, contact between both RNAPs can occur only through the terminal σ^A 1.1 domains. These domains are on flexible linkers and their positioning can be appropriately adapted.
(C) A model of a shortened DNA fragment (del17) containing P*helD* and P*anti-helD* (left) and the same DNA fragment with RNAPs accommodated at the two promoters (right). The two RNAPs encroach on each other and the presence of one RNAP on one promoter is incompatible with the simultaneous presence of another RNAP on the other promoter – the Figure was drawn to illustrate the overlap; the two RNAPs could not be simultaneously accommodated on this DNA. The models were created with the AlphaFold3 web server (Abramson *et al.*, 2024) and visualized with ChimeraX (Meng *et al.*, 2023).

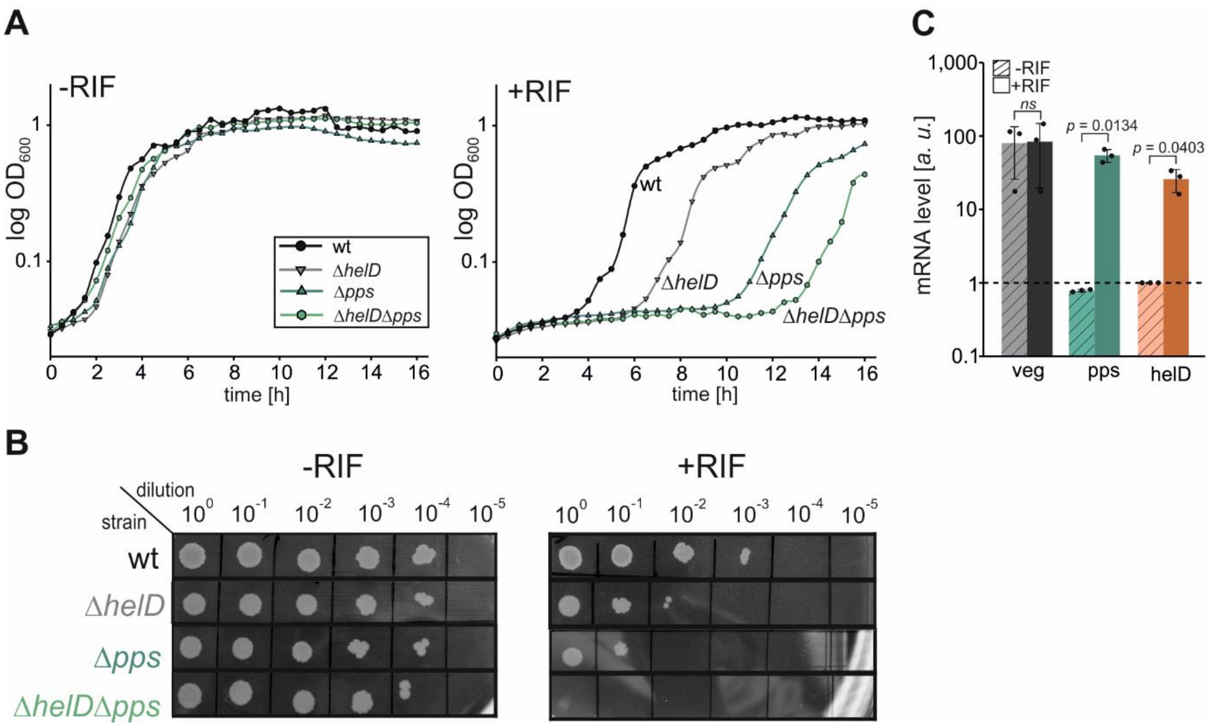

Supplementary Figure S16. Effect of *pps* on RIF resistance of *B. subtilis*

(A) Growth of strains in liquid LB medium in the absence (-RIF) or presence (+RIF) of sub-MIC concentration (0.03 µg/ml) of RIF. The strains were inoculated in a 24-well plate and the OD_{600} was measured every 30 minutes for ~ 16 hours. A representative result is shown from a total of three independent growth experiments. Black dots, wt strain (LK2711); grey triangles, Δ*helD* strain (LK2840); dark green triangles, Δ*pps* strain (LK3449); light green hexagons, Δ*helD*Δ*pps* strain (LK3451).

(B) Growth of strains on solid LB medium in the absence (-RIF) or presence (+RIF) of sub-MIC concentration (0.03 µg/ml) of RIF. A representative result is shown; the experiment was repeated three times with identical results.

(C) Relative levels of *pps*, *helD* and *veg* mRNAs determined with RT-qPCR from wt (LK2711) -/+RIF. The *helD* mRNA level in the absence of RIF (-RIF) was set as 1. The graph (y-axis log scale) shows averages from three independent experiments, the dots are individual experimental data, the error bars show ± SD. *p*-values were calculated using a two-tailed, unpaired *t*-test and indicated in the graph; "*ns*" indicates non-significance, $p > 0.05$.

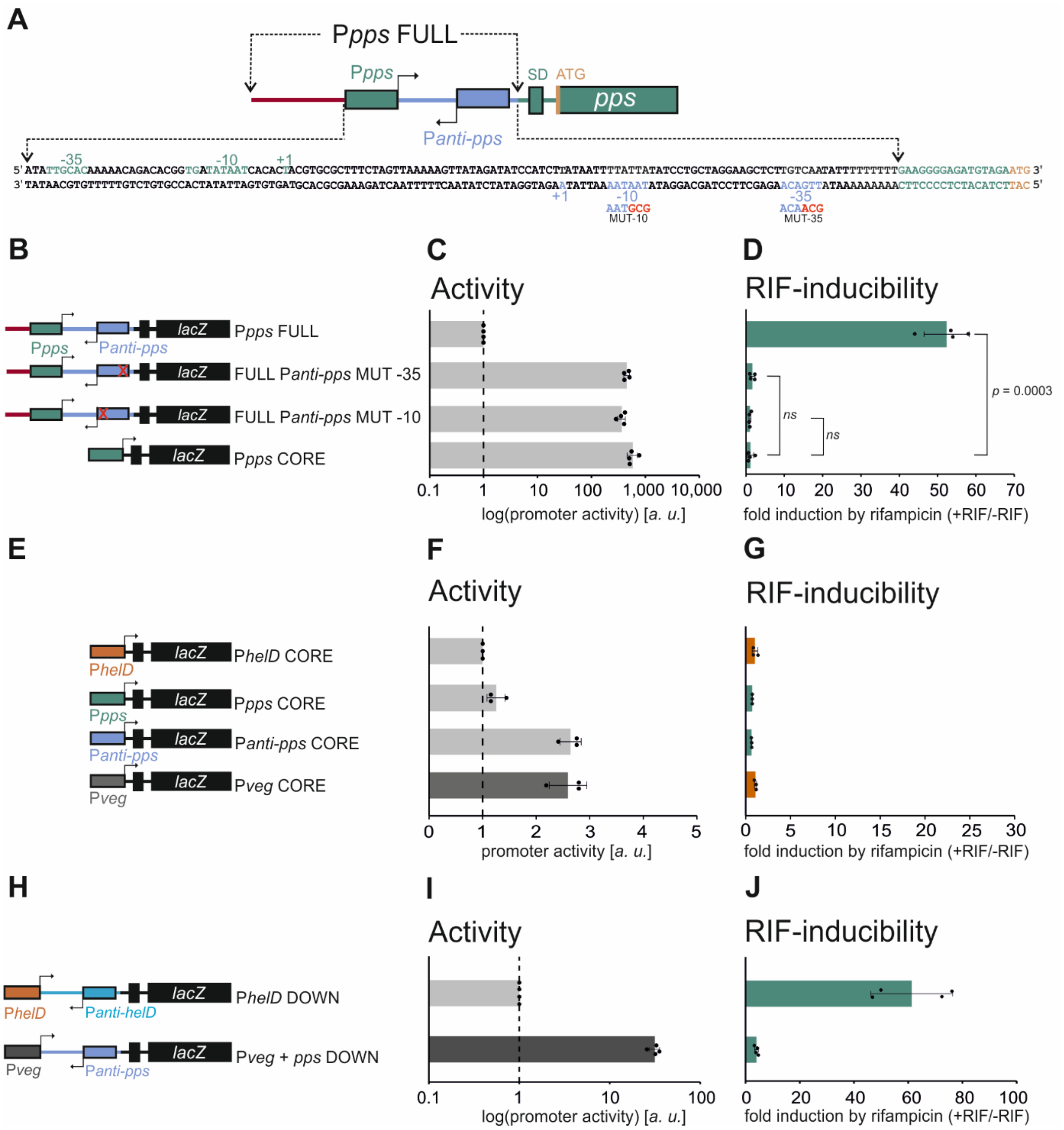

Supplementary Figure S17. Regulation of *pps* expression in the absence/presence of RIF

(A) A schematic representation (top) and relevant part of the *pps* upstream region sequence including the P*pps* and P*anti-pps* promoters (bottom). The P*pps* promoter is highlighted in green, the P*anti-pps* promoter in purple and the mutations of -10 and -35 promoter regions of P*anti-pps* are indicated with red.
(B) A scheme of promoter-*lacZ* fusions created with DNA fragments from the *pps* gene upstream region. P*pps* FULL (LK3482), FULL P*anti-pps* MUT -35 (LK3736), FULL P*anti-pps* MUT -10 (LK3735), P*pps* CORE (LK3481).
(C) Activities of promoter-*lacZ* fusions [from **(B)**] in exponential phase (OD_{600} = 0.5) in the absence of RIF. The strong P*veg* promoter was used as a control. Activity of P*pps* FULL was set as 1. The graph represents data from four independent experiments, the dots are individual experimental data, the error bars show ± SD.

(D) Inducibility of promoter-*lacZ* fusions [from **(B)**] after RIF treatment (activity in the presence of RIF to activity in its absence; +RIF/-RIF; 0.004 µg/ml). Activity without RIF for each construct were set as 1. The graph represents data from four independent experiments, the dots are individual experimental data, the error bars show ± SD. *p*-values were calculated using a two-tailed, unpaired *t*-test and indicated in the graph; "*ns*" indicates non-significance, $p > 0.05$.

(E) A scheme of the CORE promoters P*helD* (LK2970), P*pps* (LK3481), P*anti-pps* (LK3614), P*veg* (LK3040).

(F) Activities of promoter-*lacZ* fusions [from **(E)**] in exponential phase (OD_{600} = 0.5) in the absence of RIF. Activity of P*helD* CORE was set as 1. The graph represents data from three independent experiments, the dots are individual experimental data, the error bars show ± SD.

(G) Inducibility of promoter-*lacZ* fusions [from **(E)**] after RIF treatment (activity in the presence of RIF to activity in its absence; +RIF/-RIF). Activity without RIF for each construct were set as 1. The bars are averages from three independent experiments, the dots are individual experimental data, the error bars show ± SD.

(H) A scheme of the P*helD* DOWN (LK3004) and chimeric construct (P*veg* + *pps* DOWN (LK3957); in this construct, P*helD* was replaced with P*veg*).

(I) Activities of promoter-*lacZ* fusions [from **(H)**] in exponential phase (OD_{600} = 0.5) in the absence of RIF. Activity of P*helD* DOWN was set as 1. The bars show averages from four independent experiments, the dots are individual experimental data, the error bars show ± SD.

(J) Inducibility of promoter-*lacZ* fusions [from **(H)**] after RIF treatment (activity in the presence of RIF to activity in its absence; +RIF/-RIF). Activity without RIF for each construct were set as 1. The bars are averages from four independent experiments, the dots are individual experimental data, the error bars show ± SD.

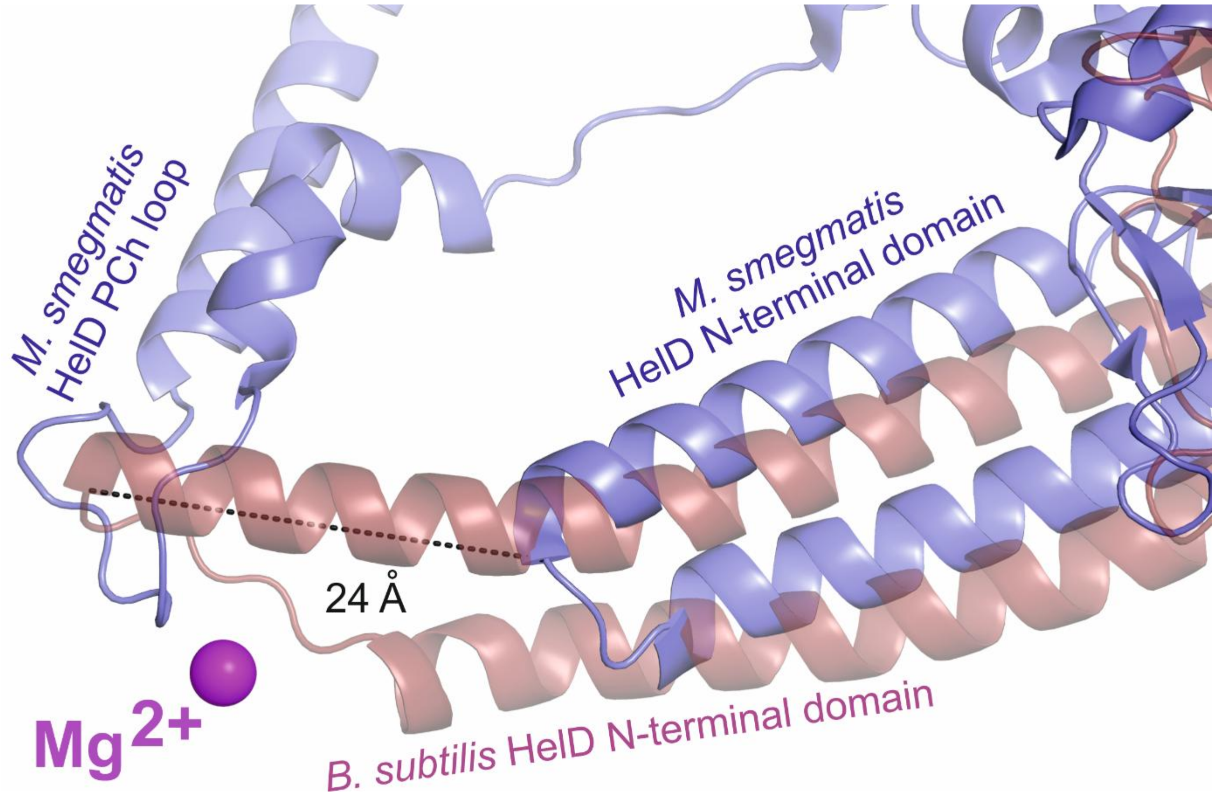

Supplementary Figure S18. Comparison of binding of N-terminal domain of Class I (*Bsu*) and PCh loop of Class II (*Msm*) HelDs into the active site of RNAP

The N-terminal domain of Class I HelD from *B. subtilis* (colored ruby-brown) reaches about 24 Å “deeper” into the RNAP than the N-terminal domain of Class II HelD from *M. smegmatis* (colored pale violet). The Class I HelD (*Bsu*) reaches beyond the RNAP active site (marked with the catalytic Mg^{2+} ion) with its N-terminal domain. The Class II HelD (*Msm*) reaches a similar position with its PCh loop. The distance between “tips” of the corresponding N-terminal domains (between *Bsu* Asp57 Cα and *Msm* Thr45 Cα) is indicated in Å and shown as black dashed line. Structures of the *B. subtilis* RNAP in complex with HelD [PDB ID: 6WVK, (Newing *et al.*, 2020)] and *M. smegmatis* RNAP in complex with HelD [PDB ID: 8QN8, (Kovaľ *et al.*, 2024)] are superimposed using the main chain atoms of the residues coordinating Mg^{2+} ion (magenta sphere) in the active site of RNAP. The graphic was created using PyMOL (The PyMOL Molecular Graphics System, Version 1.2r3pre, Schrödinger, LLC).

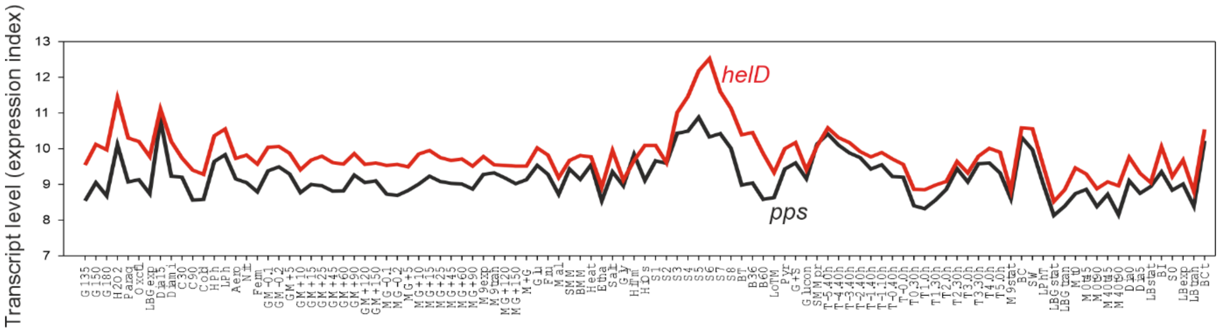

Supplementary Figure S19. Expression profiles of *helD* and *pps* genes

Data were obtained from (Nicolas *et al.*, 2012) where the authors had determined *B. subtilis* transcriptomes from >100 different conditions.

Supplementary Table S1: Bacterial strains and plasmids

Name/Original name	Construct	Description	Antibiotic resistance	Reference
[a]*E. coli*				
LK13	DH5α	General cloning strain		Laboratory strain
LK22	*Bsu sigA*	pCD2 with *rpoA* (*Bsu sigA*), BL21(DE3)	[c]Amp	(Chang and Doi, 1990)
LK25/RLG6924	pDG3661	Integrative plasmid (*amyE* site of *B. subtilis*), for β-galactosidase assays, DH5α	Amp	(Krásný and Gourse, 2004)
LK135/RLG7555	P*veg* CORE	pDG3661 with P*veg*; -38/+1, +1G, DH5α	Amp	(Krásný and Gourse, 2004)
LK180	pRLG770	Plasmid for *in vitro* transcription, DH5α	Amp	(Ross *et al.*, 1990)
LK222	pUC18	Cloning vector, DH5α	Amp	(Norrander, Kempe and Messing, 1983)
LK459	pDR110	Cloning vector, DH5α	Amp	(Meijer and Miguel-Arribas, 2024)
LK625	BL21 (DE3)	Expression strain		Laboratory strain
LK800	*Bsu* HelD	pET151/NT-His (6x), His-TEV cleavage site-HelD, BL21(DE3)	Amp	(Wiedermannová *et al.*, 2014)
LK1177/RLG7558	P*veg* CORE	pRLG770 with P*veg*; -38/+1, +1G, DH5α	Amp	(Sojka *et al.*, 2011)
LK1834	*rrnJ* P1 + P2	pRLG770 with *rrnJ* P1 + P2, DH5α	Amp	This work
LK2931	HelD native	pDG3661 with HelD under native promoter (whole upstream region); for complementation or over-expression; DH5α	Amp	This work
LK2977	P*helD* CORE	pRLG770 with P*helD*; -38/+1, +1G, DH5α	Amp	This work
LK2982	*rpoC* (β')-1xFLAG	pUC18 with *rpoC* (β')-1xFLAG construct, DH5α	Amp	This work
LK2991	P*helD* CORE	pDG3661 with P*helD*; -38/+1, +1G, DH5α	Amp	This work
LK2994	P*helD* FULL	pRLG770 with P*helD* FULL, DH5α	Amp	This work

LK3008	P*helD* FULL	pDG3661 with P*helD* FULL, DH5α	Amp	This work
LK3009	P*helD* UP	pDG3661 with P*helD* UP, DH5α	Amp	This work
LK3010	P*helD* DOWN	pDG3661 with P*helD* DOWN, DH5α	Amp	This work
LK3076	pAJS23	Vector for CRISPR-Cas9 engineering of *B. subtilis*, DH5α	[c]Kan	(Sachla, Alfonso and Helmann, 2021)
LK3082	P*helD* del17	pDG3661 with P*helD* del17, DH5α	Amp	This work
LK3109	P*helD* CORE+15	pDG3661 with P*helD* CORE+15, DH5α	Amp	This work
LK3110	P*helD* CORE+30	pDG3661 with P*helD* CORE+30, DH5α	Amp	This work
LK3111	P*helD* CORE+45	pDG3661 with P*helD* CORE+45, DH5α	Amp	This work
LK3112	P*helD* CORE+60	pDG3661 with P*helD* CORE+60, DH5α	Amp	This work
LK3229	FULL P*anti-helD* MUT-10	pRLG770 with FULL P*anti-helD* MUT-10, DH5α	Amp	This work
LK3231	FULL P*anti-helD* MUT-35	pDG3661 with FULL P*anti-helD* MUT-35, DH5α	Amp	This work
LK3232	FULL P*anti-helD* MUT-10	pDG3661 with FULL P*anti-helD* MUT-10, DH5α	Amp	This work
LK3419	P*pps* FULL	pDG3661 with P*pps* FULL, DH5α	Amp	This work
LK3420	P*pps* CORE	pDG3661 with P*pps*; -37/+4, DH5α	Amp	This work
LK3610	P*anti-helD* CORE	pRLG770 with P*anti-helD*; -40/+4, DH5α	Amp	This work
LK3611	P*anti-helD* CORE	pDG3661 with P*anti-helD*; -40/+4, DH5α	Amp	This work
LK3666	P*veg* + *helD* DOWN	pDG3661 with P*veg* + *helD* DOWN, DH5α	Amp	This work
LK3670	FULL P*anti-pps* MUT-10	pDG3661 with FULL P*anti-pps* MUT-10, DH5α	Amp	This work
LK3671	FULL P*anti-pps* MUT-35	pDG3661 with FULL P*anti-pps* MUT-35, DH5α	Amp	This work
LK3745	HelDΔN	pDG3661 with HelDΔN (HelD 204-706AA), DH5α	Amp	This work
LK3771	HelDmutTIP	pDG3661 with *helD* encoding mutations in 3 acidic AA in the N-terminal domain: D56A, D57A, D60A; including native promoter; DH5α	Amp	This work

LK3823	P*helD* FULL REVERSE	pDG3661 with P*helD* FULL REVERSE, DH5α	Amp	This work
LK3933	P*veg* + *pps* DOWN	pDG3661 with P*veg* + *pps* DOWN, DH5α	Amp	This work
LK4199	P*anti-pps* CORE	pDG3661 with P*anti-pps*; -51/+4, DH5α	Amp	This work
[a]***B.subtilis***				
LK1272	*Bsu* RNAPΔ*helD*	β' with CT-His (10x), BsB1 *helD*::MLS	[c]Cm, [c]MLS	(Wiedermannová *et al.*, 2014)
LK1723/RLG7024	*Bsu* RNAP	β' with CT-His (10x), BsB1	Cm	(Qi and Hulett, 1998)
LK1933	Background	*amyE*::pDG3661, BsB1	Cm	This work
LK2711	Wt	BsB1(BaSysBio)		(Nicolas *et al.*, 2012)
LK2840	Δ*helD*	BsB1 *helD*::MLS	MLS	This work
LK2934	*helD* OE	BsB1 *amyE*::CM *helD*	Cm	This work
LK2935	*helD* COM	BsB1 *helD*::MLS *amyE*::CM *helD*	Cm, MLS	This work
LK2970	P*helD* CORE	*amyE*::P*helD-lacZ*; -38/+1, +1G	Cm	This work
LK2973	P*helD* CORE, Δ*helD* BsB1	*amyE*::P*helD-lacZ*; -38/+1, +1G, BsB1 *helD*::MLS	Cm, MLS	This work
LK2976	Background, Δ*helD* BsB1	*amyE*::pDG3661, BsB1 *helD*::MLS	Cm, MLS	This work
LK3004	P*helD* DOWN	*amyE*:: P*helD*(DOWN)-*lacZ*, BsB1	Cm	This work
LK3005	P*helD* FULL	*amyE*::P*helD*(FULL)-*lacZ*, BsB1	Cm	This work
LK3006	P*helD* DOWN, Δ*helD* BsB1	*amyE*::P*helD*(DOWN)-*lacZ*; BsB1 *helD*::MLS	Cm, MLS	This work
LK3007	P*helD* FULL, Δ*helD* BsB1	*amyE*::P*helD*(FULL)-*lacZ*; BsB1 *helD*::MLS	Cm, MLS	This work
LK3038	P*helD* UP	*amyE*:: P*helD*(UP)-*lacZ,* BsB1	Cm	This work
LK3040	P*veg* CORE	*amyE*::P*veg-lacZ*; -38/+1, +1G*,* BsB1	Cm	This work
LK3114	P*helD* del17	*amyE*::P*helD*(Δ+17/+34)-*lacZ,* BsB1	Cm	This work
LK3117	P*helD* CORE+15	*amyE*::P*helD*(CORE+15)-*lacZ,* BsB1	Cm	This work
LK3118	P*helD* CORE+30	*amyE*::P*helD*(CORE +30)-*lacZ,* BsB1	Cm	This work

LK3119	P*helD* CORE+45	*amyE*::P*helD*(CORE +45)-*lacZ,* BsB1	Cm	This work
LK3120	P*helD* CORE+60	*amyE*::P*helD*(CORE +60)-*lacZ,* BsB1	Cm	This work
LK3132	*Bsu* RNAP-FLAG	β'-FLAG integrated into the wt *B. subtilis* (BsB1) genome	[c]Spc	This work
LK3233	FULL P*anti-helD* MUT-35	*amyE*::P*helD*(FULL P*anti-helD* MUT-35)-*lacZ*, BsB1	Cm	This work
LK3234	FULL P*anti-helD* MUT-10	*amyE*::P*helD*(FULL P*anti-helD* MUT-10)-*lacZ,* BsB1	Cm	This work
LK3449	Δ*pps*	BsB1 *pps*::KAN	Kan	This work
LK3451	Δ*helD*Δ*pps*	BsB1 *helD*::MLS *pps*::KAN	Kan, MLS	This work
LK3481	P*pps* CORE	*amyE*::P*pps-lacZ*; -37/+4, BsB1	Cm	This work
LK3482	P*pps* FULL	*amyE*::P*pps*(FULL)-*lacZ*, BsB1	Cm	This work
LK3614	P*anti-helD* CORE	*amyE*::P*anti-helD-lacZ*; -40/+4, BsB1	Cm	This work
LK3735	FULL P*anti-pps* MUT-10	*amyE*::P*pps*(FULL P*anti-pps* MUT-10)-*lacZ*, BsB1	Cm	This work
LK3736	FULL P*anti-pps* MUT-35	*amyE*::P*pps*(FULL P*anti-pps* MUT-35)-*lacZ*, BsB1	Cm	This work
LK3772	HelDΔN, Δ*helD* BsB1	*amyE*::CM *helD*ΔN, BsB1 *helD*::MLS	Cm, MLS	This work
LK3784	HelDtipMUT, Δ*helD* BsB1	*amyE*::CM *helD*tipMUT, BsB1 *helD*::MLS	Cm, MLS	This work
LK3789	P*veg* + *helD* DOWN	*amyE*::P*veg*(P*helD* DOWN)-*lacZ*, BsB1	Cm	This work
LK3826	P*helD* FULL REVERSE	*amyE*::P*helD*(FULL REVERSE)-*lacZ*, BsB1	Cm	This work
LK3957	P*veg* + *pps* DOWN	*amyE*::P*veg*(P*pps* DOWN)-*lacZ*, BsB1	Cm	This work
LK4200	P*anti-pps* CORE	*amyE*::P*anti-pps-lacZ*; -51/+4, BsB1	Cm	This work
LK4387	RIF resistant RNAP	Mutation in *rpoB* gene (H482Y) leading to RIF resistant RNAP, BsB1	[c]RIF	This work

LK4437	P*helD* CORE, RIF^R RNAP	*amyE*::P*helD-lacZ*; -38/+1, +1G; BsB1	Cm	This work
LK4439	P*helD* FULL, RIF^R RNAP	*amyE*::P*helD*(FULL)-*lacZ*; BsB1	Cm	This work
LK4445	Background, RIF^R RNAP	*amyE*::pDG3661; BsB1	Cm	This work
[a]***M. smegmatis***				
LK2980	Wt	*M. smegmatis* mc^2 155		Laboratory strain

[a] *E. coli – Escherichia coli; B. subtilis – Bacillus subtilis; M. smegmatis – Mycobacterium smegmatis*

[b] *Bsu – Bacillus subtilis*

[c] Amp – ampicillin; Cm – chloramphenicol; Kan – kanamycin; MLS – erythromycin, lincomycin; RIF – rifampicin; Spc - spectinomycin

Supplementary Table S2: List of primers

Primer #/Name	Sequence 5′→ 3′	Description
923/dsDNA_competitor_F	CCGGAATTCAAATATTTGTTGTTAACTCTTGACAAAAGTGTTAAATTG TGCTATACTGTATTGGTTCTCAAGCTTCCG	Primers for preparation of dsDNA competitor for single round transcriptions *in vitro*
924/dsDNA_competitor_R	CGGAAGCTTGAGAACCAATACAGTATAGCACAATTTAACACTTTTGT CAAGAGTTAACAACAAATATTTGAATTCCGG	
1197/*veg*_qPCR_F	GCGAAGACGTTGTCCGATA	Primers for qPCR to determine relative expression levels of the *B. subtilis veg* gene
1198/*veg*_qPCR_R	CTCAGCTAAAATGCCCGAAC	
1281/MS_16SrRNA_IVT_F	TACGTAATACGACTCACTATAGGGAGACAGCTCGTGTCGTGAGAT GT	Primers for preparation of recovery marker (RM) template (fragment of *M. smegmatis* 16S rRNA) for subsequent *in vitro* transcription with T7 RNAP
1282/MS_16SrRNA_IVT_R	CGTTGCTGATCTGCGATTAC	
2037/*rrnJ*_F	GCGAATTCAAGAGCGGTATCCTCCATAG	Primers for cloning of *rrnJ* P1 and P2 promoters into pRLG770 to generate molecular size RNA marker of 238 nt (from P1) and 153 nt (from P2)
2039/*rrnJ* P1+P2_R	GCAAGCTTGACTTTATTATTATAACTCG	
2088/*hrcA*_F	GTCGGACCTGTTAAGCATTAC	Forward primer for preparation of *hrcA* DNA fragments to generate molecular size RNA marker in combination with #2089, #2131
2089/*hrcA*_R_91 nt	CGTTGATTATAACCTGAAGG	Primer for preparation of *hrcA* DNA fragment to generate RNA of 91 nt in combination with #2088

2131/*hrcA*_R_80 nt	ACCTGAAGGATCAGCAGCTGA	Primer for preparation of *hrcA* DNA fragment to generate RNA of 80 nt in combination with #2088
2618/Ms_16SrRNA_F	TCATGTTGCCAGCACGTTAT	Primers for qPCR to determine relative levels of recovery marker (RM)
2619/Ms_16SrRNA_R	AAGGGGCATGATGACTTGAC	
3583/BS_*rpoC*_LA_F	CCTGCAGGTCGACTCTAGAGGGATCCTTCTTAAAGTGAC	Primers for preparation of the left arm of the construct for homology recombination to generate FLAG-tagged RNAP
3584/BS_*rpoC*_LA_R	CTTTGTAGTCTTCAACCGGGACCATATC	
3624/Flag+term_F	CCCGGTTGAAGACTACAAAGACGATGACGACAAGTAACTGATTTAA CTCTGCTGAAAG	Primers for preparation of the FLAG-peptide, STOP codon and natural terminator of the construct for homology recombination to generate FLAG-tagged RNAP
3586/Flag+term_R	AGATCGATCCCATAAATATATCTGCTGAAAGAC	
3587/SpcR_F	TATATTTATGGGATCGATCTGTATAATAAAGAATAATTATTAATC	Primers for preparation of the spectinomycin resistance cassette for the FLAG-tagged RNAP
3588/SpcR_R	GTGACTTTTTCAGCTAATAAAAAAATTTGCTAAAG	
3589/BS_*rpoC*_RA_F	TTATTAGCTGAAAAAGTCACTCTATGAGAAG	Primers for preparation of the right arm of the construct for homology recombination to generate FLAG-tagged RNAP
3590/BS_*rpoC*_RA_R	CAGCTATGACCATGATTACGGAATTCCTTTGAGTTTTCTAC	
3628/HelDnative_R	AAGCTGTCAAACATGAGAATTCGATATAAAACGGCTATGCTTCAT	Primers for complementation/over-expression of *helD* under native promoter for PCR cloning into pDG3661
3673/HelDnative_F2	GTGTATCAACAAGCTGGGGATCCTAGCTGCATAATATAAATAACCC	

3698/HelD_5′RACE	TCGAGCTCCTTCAGCACCTC	Primer for TD PCR (5′RACE), starting +49 bp from ATG (start of translation) of *B. subtilis helD*
3699/P*helD*_REV	GACGTTTCCAGAAACCGCTC	Gene specific primer for reverse transcription; starting +76 from ATG of *helD*
3747/P*helD* CORE_F	AATTCACCTTGATTGGTATTGGGTGAATGTGCTATTATAAATTGTCTA	Primers for P*helD* CORE (-39/+2) cloning into pRLG770/pDG3661
3748/ P*helD* CORE _R	AGCTTAGACAATTTATAATAGCACATTCACCCAATACCAATCAAGGTG	
3800/P*helD* FULL_F	CGCGAATTCTAGCAAAAAAACCGATTTC	Primers for P*helD* FULL cloning into pRLG770/pDG3661
3801/ P*helD* FULL _R	GCGAAGCTTAGAAAATAGTTGACAAAC	
3802/ P*helD* UP_R	GCGAAGCTTAGACAATTTATAATAGCAC	Primer for P*helD* UP cloning, into pDG3661, in combination with #3800
3803/P*helD* DOWN_F	CGCGAATTCACCTTGATTGGTATTGGG	Primers for P*helD* DOWN, cloning into pRLG770/pDG3661 in combination with #3801
3998/P*helD* del17	TTGTATTTGCCAGTTTGCATTATCCAATTATATTTTACAGTATTTCATCTATGTT	Primer for mutagenesis P*helD* del17
4094/P*helD* CORE+15_F	AATTCACCTTGATTGGTATTGGGTGAATGTGCTATTATAAATTGTATTTGCCAGTTTGCA	Primers for P*helD* CORE+15 cloning into pDG3661
4095/P*helD* CORE+15_R	AGCTTGCAAACTGGCAAATACAATTTATAATAGCACATTCACCCAATACCAATCAAGGTG	
4096/P*helD* CORE+30_R	CGCAAGCTTTATTAAATATATGATGCAA	Primer for P*helD* CORE+30 cloning into pDG3661, in combination with #3801
4097/P*helD* CORE+45_R	CGCAAGCTTATAATTGGATACATATATT	Primer for P*helD* CORE+45 cloning into pDG3661, in combination with #3801

4098/P*helD* CORE+60_R	CGCAAGCTTGAAATACTGTAAAATATAA	Primer for P*helD* CORE+60 cloning into pDG3661, in combination with #3801
4295/*helD*_qPCR_F	GTCCATCTACGCGCATACCA	Primers for qPCR to determine relative expression levels of the *helD* gene
4296/*helD*_qPCR_R	GTTTTGACAACGAGCGGCAT	
4363/FULL P*anti-helD* MUT-35_R	CGCAAGCTTAGAAAATAGGCAACAAACATAGATGAAATACTGTAA	Primer for cloning of FULL P*anti*-helD MUT-35 into pRLG770/pDG3661; in combination with #3800
4364/FULL P*anti-helD* MUT-10	ATATATGTATCCAATTATATGCGAGGGTATTTCATCTATGTTTGTC	Primer for FULL P*anti*-helD MUT-10 mutagenesis
4400/*pps*_qPCR_F	CTACGGACGAAAAGAAGGCG	Primers for qPCR determine relative expression levels of the *pps* gene
4401/*pps*_qPCR_R	CGCTTCAGGGATGGGGAATA	
4406/P*pps* CORE_F	AATTCATATTGCACAAAAACAGACACGGTGATATAATCACACTACGA	Primers for cloning of P*pps* CORE into pRLG770/pDG3661
4407/P*pps* CORE _R	AGCTTCGTAGTGTGATTATATCACCGTGTCTGTTTTTGTGCAATATG	
4408/P*pps* FULL_F	CGCGAATTCATATTGCACAAAAACAGAC	Primers for cloning of P*pps* FULL into pRLG770/pDG3661
4409/P*pps* FULL_R	GCGAAGCTTAAAAAAAAATATTGACAAG	
4554/P*anti-helD*_F	AATTCTAGTTGACAAACATAGATGAAATACTGTAAAATATAATTGGATAA	Primers for cloning of P*anti-helD* CORE into pRLG770/pDG3661
4555/ P*anti-helD*_R	GATCAACTGTTTGTATCTACTTTATGACATTTTATATTAACCTATTTCGA	
4730/ FULL P*anti-pps* MUT-35	TCCTGCTAGGAAGCTCTTGTTGCTATTTTTTTTTAAGCTT	Mutagenesis of FULL P*anti-pps* MUT-35 in *pps* 5′ UTR, for for Restriction Free Cloning
4731/FULL P*anti-pps* MUT-10	ATATCCATCTTATAATTTTACGCTATCCTGCTAGGAAGCTCTT	Mutagenesis of FULL P*anti-pps* MUT-10 in *pps* 5′ UTR, for for Restriction Free Cloning

4746/HelDΔN_F	CTATTTTCTGGAGGTGCCGTTCATGACCCAAATGAAAAACATCGTG	HelDΔN (204-706AA) variant (used in combination with #3628), for cloning into pDG3661
4747/HelDmutTIP	GTTAAGGTTAATTTTGCTGCTGCCCATGCAGCGATTGAAACGATG	HelD with D56, D57, E60 aa mutated to alanines, for Restriction Free Cloning
4748/P*helD* FULL REVERSE_F	CGCAAGCTTTAGCAAAAAAACCGATTTC	For P*helD* FULL cloning in reverse direction (*HindIII-EcoRI*)
4749/P*helD* FULL REVERSE _R	GCGGAATTCAGAAAATAGTTGACAAAC	
5062/P*anti-pps* CORE_F	AATTCTTCAAAAAAAAATATTGACAAGAGCTTCCTAGCAGGATATAATAAAATTATAAGAA	Oligonucleotides for P*anti-pps* CORE promoter
5063/P*anti-pps* CORE _R	AGCTTTCTTATAATTTTATTATATCCTGCTAGGAAGCTCTTGTCAATATTTTTTTTTGAAG	
5239/P*helD* T7 RNA probe_F	GATCCGAATAATACGACTCACTATAGGCCGTTTTCAATAAAATCAATCCG	PCR template for T7 *in vitro* transcription of RNA probe anti-P*helD* (Northern blot)
5240/P*helD* T7 RNA probe R	GATAAGGAATGGAAGGAAGAGCAG	
5284/T7 P*anti-helD* template	GATCCGAATAATACGACTCACTATAGCCCAAACCTTGATTGGTATTGGGTGAATGTGCTATTATAAATTGTATTTGCCAGTTTGCATCATATATTTAATATATGTATC	Template used to generating anti-P*anti-helD* probe (Northern blot)
5285/P*anti-helD* T7 RNA probe_F	GATCCGAATAATACGACTC	PCR template for T7 *in vitro* transcription of RNA probe anti-P*anti-helD* (Northern blot)
5286/P*anti-helD* T7 RNA probe_R	GATACATATATTAAATATATGATGCAAAC	
Oligo1_SSS1016	CTGGAGCACGAGGACACTGACATGGACTGAAGGAGTrArGrArArA	5′RACE adapter as in (Martini et al., 2019)
Oligo2_SSS1017	CTGGAGCACGAGGACACTGA	5′RACE oligonucleotide (Martini et al., 2019)
Oligo3_P*veg*+*helD* DOWN_F	CGCGAATTCTATTTGACAAAAATGGGCTCGTGTTGTACAATAAATGTGTATTTGCCAGTTTGCATCATATATTTAATATATGTATCCAATTATATTTTACAGTATTTCATCTATGTTTGTCAACTATTTTCTAAGCTTCGC	Oligonucleotides for the P*veg*+*helD* DOWN chimeric variant for cloning into pDG3661

Oligo4_P*veg*+*helD* DOWN _R	GCGCTTAAGATAAACTGTTTTTACCCGAGCACAACATGTTATTTACACATAAACGGTCAAACGTAGTATATAAATTATATACATAGGTTAATATAAAATGTCATAAAGTAGATACAAACAGTTGATAAAAGATTCGAAGCG	
Oligo5_P*veg*+*pps* DOWN_F	CGCGAATTCTATTTGACAAAAATGGGCTCGTGTTGTACAATAAATGTGACGTGCGCTTTCTAGTTAAAAAGTTATAGATATCCATCTTATAATTTTATTATATCCTGCTAGGAAGCTCTTGTCAATATTTTTTTTTAAGCTTCGC	Oligonucleotides for the P*veg*-*pps* DOWN chimeric variant for cloning into pDG3661
Oligo6_P*veg*+*pps* DOWN_R	GCGAAGCTTAAAAAAAAATATTGACAAGAGCTTCCTAGCAGGATATAATAAAATTATAAGATGGATATCTATAACTTTTTAACTAGAAAGCGCACGTCACATTTATTGTACAACACGAGCCCATTTTTGTCAAATAGAATTCGCG	

F – indicates forward primer

R – indicates reverse primer

Supplementary Table S3: List of the dual-promoter sites found in selected genomes from RefSeq

This Table is due to its size attached as Supplementary Table S3.xlsx.

Supplementary Table S4: List of protein cluster representatives

The proteins are associated with (downstream of) dual-promoter sites listed in Supplementary Table S4.

This Table is due to its size attached as Supplementary Table S4.xlsx.

Supplementary Table S5: Proteome of *Bacillus subtilis* +RIF/-RIF

This Table is due to its size attached as Supplementary Table S5.xlsx.

Supplementary references

Abramson, J. *et al.* (2024) ‘Accurate structure prediction of biomolecular interactions with AlphaFold 3’, *Nature*, 630(8016), pp. 493–500. Available at: https://doi.org/10.1038/S41586-024-07487-W,.

Chang, B.-Y. and Doi, R.H. (1990) ‘Overproduction, Purification, and Characterization of Bacillus subtilis RNA Polymerase SigA Factor’, *JOURNAL OF BACTERIOLOGY*, 172(6), pp. 3257–3263.

Kovaľ, T. *et al.* (2024) ‘Mycobacterial HelD connects RNA polymerase recycling with transcription initiation’, *Nature Communications 2024 15:1*, 15(1), pp. 1–20. Available at: https://doi.org/10.1038/S41467-024-52891-5.

Krásný, L. and Gourse, R.L. (2004) ‘An alternative strategy for bacterial ribosome synthesis: Bacillus subtilis rRNA transcription regulation.’, *The EMBO journal*, 23(22), pp. 4473–4483. Available at: https://doi.org/10.1038/sj.emboj.7600423.

Lin, W. *et al.* (2017) ‘Structural Basis of Mycobacterium tuberculosis Transcription and Transcription Inhibition’, *Molecular Cell*, 66(2), pp. 169-179.e8. Available at: https://doi.org/10.1016/j.molcel.2017.03.001.

Martini, M.C. *et al.* (2019) ‘Defining the transcriptional and post-transcriptional landscapes of mycobacterium smegmatisin aerobic growth and hypoxia’, *Frontiers in Microbiology*, 10(MAR), pp. 1–17. Available at: https://doi.org/10.3389/fmicb.2019.00591.

Meijer, W.J.J. and Miguel-Arribas, A. (2024) ‘Genetic Engineering of Bacillus subtilis Using Competence-Induced Homologous Recombination Techniques’, *Methods in Molecular Biology*, 2819, pp. 241–260. Available at: https://doi.org/10.1007/978-1-0716-3930-6_12,.

Meng, E.C. *et al.* (2023) ‘UCSF ChimeraX: Tools for structure building and analysis’, *Protein Science*, 32(11). Available at: https://doi.org/10.1002/PRO.4792,.

Newing, T.P. *et al.* (2020) ‘Molecular basis for RNA polymerase-dependent transcription complex recycling by the helicase-like motor protein HelD’, *Nature Communications*, 11(1), pp. 1–11. Available at: https://doi.org/10.1038/s41467-020-20157-5.

Nicolas, P. *et al.* (2012) ‘Condition-dependent transcriptome reveals high-level regulatory architecture in Bacillus subtilis’, *Science*, 335(6072), pp. 1103–1106. Available at: https://doi.org/10.1126/science.1206848.

Norrander, J., Kempe, T. and Messing, J. (1983) ‘Construction of improved M13 vectors using oligodeoxynucleotide-directed mutagenesis’, *Gene*, 26(1), pp. 101–106. Available at: https://doi.org/10.1016/0378-1119(83)90040-9,.

Pei, H.H. *et al.* (2020) ‘The δ subunit and NTPase HelD institute a two-pronged mechanism for RNA polymerase recycling’, *Nature communications*, 11(1). Available at: https://doi.org/10.1038/S41467-020-20159-3.

Qi, Y. and Hulett, F.M. (1998) ‘PhoP~P and RNA polymerase sigma(A) holoenzyme are sufficient for transcription of Pho regulon promoters in Bacillus subtilis: PhoP~P activator sites within the coding region stimulate transcription in vitro’, *Molecular Microbiology*, 28(6), pp. 1187–1197. Available at: https://doi.org/10.1046/j.1365-2958.1998.00882.x.

Ross, W. *et al.* (1990) ‘E. coli Fis protein activates ribosomal RNA transcription in vitro and in vivo’, *EMBO Journal*, 9(11), pp. 3733–3742. Available at: https://doi.org/10.1002/j.1460-2075.1990.tb07586.x.

Sachla, A.J., Alfonso, A.J. and Helmann, J.D. (2021) ‘A Simplified Method for CRISPR-Cas9 Engineering of Bacillus subtilis’, *Microbiology Spectrum*, 9(2). Available at: https://doi.org/10.1128/SPECTRUM.00754-21,.

Sojka, L. *et al.* (2011) ‘Rapid changes in gene expression: DNA determinants of promoter regulation by the concentration of the transcription initiating NTP in Bacillus subtilis.’, *Nucleic Acids Research*, 39(11), pp. 4598–611. Available at: https://doi.org/10.1093/nar/gkr032.

Wiedermannová, J. *et al.* (2014) ‘Characterization of HelD, an interacting partner of RNA polymerase from Bacillus subtilis’, *Nucleic Acids Research*, 42(8), pp. 5151–5163. Available at: https://doi.org/10.1093/nar/gku113.
